# A library of new bifunctional alkenes obtained by a highly regiodivergent silylation of 1,5-hexadiene†

**DOI:** 10.1039/d1ra07468g

**Published:** 2021-12-07

**Authors:** Rafał Januszewski, Bartosz Orwat, Jan Merna, Ireneusz Kownacki

**Affiliations:** Faculty of Chemistry, Adam Mickiewicz University in Poznan Uniwersytetu Poznanskiego 8 61-614 Poznan Poland r.janusz@amu.edu.pl; Center for Advanced Technology, Adam Mickiewicz University in Poznan Uniwersytetu Poznanskiego 10 61-614 Poznan Poland; Department of Molecular Physics, Lodz University of Technology Zeromskiego 116 90-924 Lodz Poland; University of Chemistry and Technology in Prague Technická 5 166 28 Prague 6 Czech Republic

## Abstract

An efficient methodology for the synthesis of two groups of silicon-containing alkenes is reported. It includes a highly regioselective functionalization of 1,5-hexadiene through hydrosilylation and dehydrogenative silylation with organofunctional silanes and siloxanes. The established conditions enable selective monofunctionalization of 1,5-hexadiene regardless of the organosilicon modifier used as well as the type of functional group bonded to the silicon-based compound. All products were isolated and fully characterized by NMR spectroscopy and MS techniques.

## Abstract 2

An efficient methodology for the synthesis of two groups of silicon-containing alkenes is reported.

## Graphical abstract

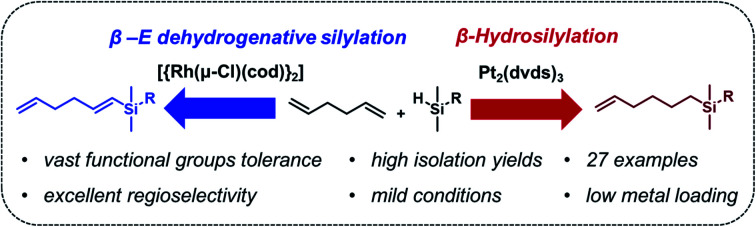

## Introduction

High reactivity of the C=C bonds in a wide gamut of catalytic and stoichiometric transformations makes alkenes an undoubtedly important class of compounds and basic building blocks in organic chemistry.^1^ Therefore, functional olefins are commonly applied as intermediates in the synthetic routes and can be easily transformed into the desired products or may be used as carriers of physicochemical properties.^2–5^ Moreover, the relatively low cost and widespread availability of functional alkenes make them widely used, *inter alia*, in the synthesis of novel monomers,^6^ functional polymers,^7^ and to enhance hydrophobic^8^ or flame retardant properties.^9^ On the other hand, the presence of a silicon atom in the structure provides many benefits such as better solubility in nonpolar solvents, improvement of thermal stability and/or flame retardancy of silicon-containing polymeric materials.^10,11^ Therefore, efficient synthetic protocols enabling a high-yield synthesis of unique alkenyl-functionalized organosilicon derivatives are highly desirable.

Considering the available literature data on suitable protocols leading to this type of compounds, the hydrosilylation of multiple bonds is the most popular synthetic approach enabling an efficient preparation of new molecular and macromolecular organosilicon compounds.^12–16^ Remarkable tolerance of a wide range of functional groups, low catalyst loading, and high conversions of the reactants made hydrosilylation reaction the fundamental process in both, laboratory and industrial applications.^17,18^ The catalytic hydrosilylation can be complicated by side reactions, among which the most disturbing are alkene isomerization and/or dehydrogenative silylation accompanied with hydrogenation of olefin. The above-mentioned side transformations have been mainly reported to occur in the modification of molecular olefins such as styrene and its derivatives.^19–24^ Nevertheless, dehydrogenative silylation has also been observed during the silylfunctionalization of organic and organosilicon polymers.^25,26^ However, due to the possibility of easy activation of the multiple C–C bonds, there has recently been a significant increase in interest in alkenylsilanes and their synthesis. Conventional routes to target compounds involve transformations of vinylsilanes *via* silylative coupling,^27,28^ cross metathesis^29,30^ and silyl-Heck coupling.^31,32^ However, the hydrosilylation is still the most convenient and the most often used synthetic tool allowing direct access to new organosilicon derivatives.^33^ Nevertheless, the direct regioselective dehydrogenative silylation of alkenes seems to be more preferable than the hydrosilylation of alkynes, thanks to their widespread availability and lower cost when compared to alkynes. Therefore, attempts for selective dehydrogenative silylation of alkenes have been undertaken.^34–39^ However, most of the studies reported so far have concerned selective functionalization of styrene derivatives. Moreover, mainly inert alkyl-,^19,20,22,24,38^ alkylaryl-,^19,22,24,36^ trialkoxysilanes^19,22^ as well as alkylsiloxanes^24,34–36^ were applied as silylation agents. Hence, the development of methodologies for the selective functionalization of alkenes with organofunctional silanes and siloxanes is still highly desirable.

Herein, we report studies concerning an efficient synthesis of novel, silicon-containing bifunctional alkenes obtained through highly regioselective mono-hydrosilylation and mono-dehydrogenative silylation of 1,5-hexadiene. The presented approach enables the formation of functional organosilicon compounds containing the desired organic groups with specific properties or/and reactivity, as well as a terminal alkene moiety that can be used in subsequent catalytic transformations.

## Results and discussion

**Scheme 1:**
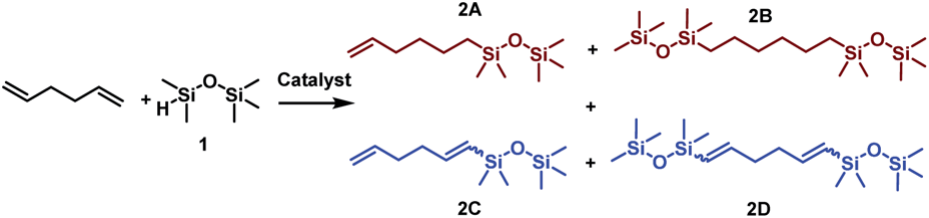
Main products distribution during functionalization of 1,5-hexadiene with pentamethyldisiloxane.

Considering the structure of 1,5-hexadiene, its selective functionalization may be problematic because of the presence of two active, terminal C=C groups. Moreover, the addition of silicon hydrides to alkenes can lead to the formation of two regioisomers, known as Markovnikov and anti-Markovnikov addition products, respectively. As mentioned in the previous paragraph, transition metal complex-catalyzed reactions of silylfunctionalization of olefins may also be accompanied by some undesirable side processes. This means that during the silylation of 1,5-hexadiene, even a dozen of various products may be formed. The four main products, without breakdown by isomerism are presented in Scheme 1. At the beginning of our studies, special attention was paid to the reaction conditions so that to optimize them towards the selective formation of one type of product. To find the best conditions enabling the monofunctionalization of 1,5-hexadiene, a model reaction of a diene with pentamethyldisiloxane (1) in the presence of four commercially available catalysts was performed (Table 1).

**Table 1:** Optimization of the functionalization protocol of 1,5-hexadiene with pentamethyldisiloxane

Entry	Cat.	Conv. of 1 a [%]	Ratio [1] : [diene]	2A/2B b	2C/2D b (*E*/*Z*)^c^
1^d^	C1	96	1 : 1	63/37	—
2^e^		99	1 : 1.5	77/23	—
3^f^		99	1 : 2	82/18	—
4^g^		98	1 : 2	85/15	—
5^h^		99	1 : 3	90/10	—
6^i^		99	1 : 4	93/7	—
7^j^		99	1 : 5	95/5	—
8^k^		99	1 : 10	98/2	—
9^j^	C2	96	1 : 5	93/7	—
10^k^		99	1 : 10	96/4	—
11^j^	C3	98	1 : 5	93/7	—
12^k^		99	1 : 10	97/3	—
13^l^	C4	28	1 : 5	96/1	3
14^m^		96	1 : 2	57/4	36/3 (94/6)
15^n^		98	1 : 3	24/3	72/1 (95/5)
16^o^		99	1 : 4	3/1	95/1 (95/5)
17^p^		99	1 : 5	—	99/1 (95/5)

a Calculated by GC, using solvent as standard.

b Calculated by GC.

c Calculated by ^1^H NMR, C1 – [Pt_2_(dvds)_3_], C2 – H_2_PtCl_6_, C3 – Pt in 1-octanol/1-octanal, C4 – [{Rh(μ-Cl)(cod)}_2_]. Reaction conditions: 50 °C, 2 mL of PhMe, 0.25 g (1.68 mmol) of 1.

d 1.68 mmol of 1,5-hexadiene.

e 2.52 mmol of 1,5-hexadiene.

f 3.36 mmol of 1,5-hexadiene.

g 3.36 mmol of 1,5-hexadiene in 5 mL of PhMe.

h 5.04 mmol of 1,5-hexadiene in 5 mL of PhMe.

i 6.72 mmol of hexadiene in 5 mL of PhMe.

j 8.4 mmol of 1,5-hexadiene in 5 mL of PhMe.

k 16.8 mmol of 1,5-hexadiene, neat, room temperature. ^d–f^ [Pt] : [HSi] = 10^−4^ : 1. ^g–k^ [Pt] : [HSi] = 2 × 10^−5^ : 1. Conditions for C4 catalyzed reactions: [Rh] : [HSi] = 2 × 10^−4^ : 1.

l r.t., 8.4 mmol of 1,5-hexadiene in 2 mL of PhMe. ^m–p^ reaction conditions: 50 °C, 2 mL PhMe, 0.25 g (1.68 mmol) of 1.

m 3.36 mmol of hexadiene.

n 5.04 mmol of 1,5-hexadiene.

o 6.72 mmol of 1,5-hexadiene.

p 8.4 mmol of 1,5-hexadiene.

**Fig. 1:**
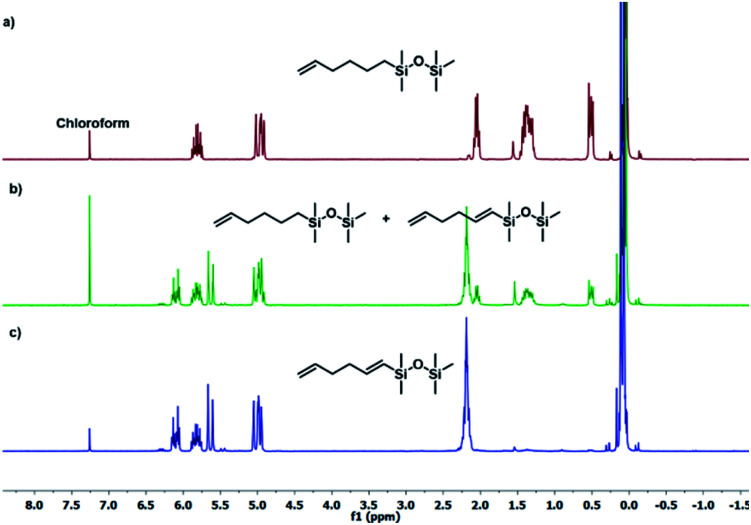
Exemplary ^1^H NMR spectra of substituted hexadiene. (a) Product 2A (Table 1, entry 8), (b) mixture of products 2A + 2C (Table 1, entry 15), (c) product 2C (Table 1, entry 17). Spectra were recorded in CDCl_3_.

**Fig. 2:**
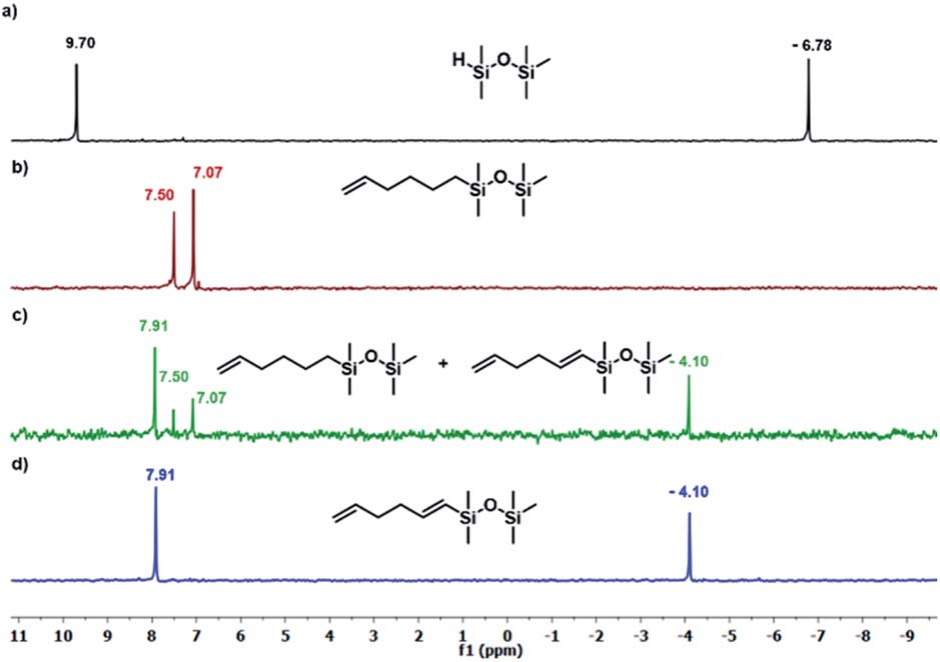
The ^29^Si NMR spectra disiloxane and its derivatives. (a) Pentamethyldisiloxane, (b) product 2A (Table 1, entry 8), (c) mixture of products 2A + 2C (Table 1, entry 15), (c) product 2C (Table 1, entry 17). Spectra were recorded in CDCl_3_.

The results presented in Table 1 clearly demonstrate that the selected Pt-based catalysts proved to be highly active hydrosilylation promotors and enabled high conversion of 1 under mild conditions. Unfortunately, the target product 2A was always accompanied by the unwanted, bissilylated derivative 2B. Moreover, no dehydrogenative silylation products were observed, regardless of the stoichiometric ratios, catalyst loading, and temperature set in the catalytic tests. As 1,5-hexadiene is a cheap reagent when compared to organosilicon compounds and can be easily removed from the postreaction mixture by distillation, thanks to its low boiling point (70 °C), we decided to significantly increase the excess of this alkene, which led to the formation of the desired product 2A with good selectivity under mild conditions (Table 1, entry 8, r.t., 2 × 10^−5^ mol Pt per mol HSi). At this point, it should be emphasized that C1 complex (Karstedt's complex) turned out to be more selective than other Pt-based catalysts. On the other hand, a commercially available rhodium complex, namely, [{Rh(μ-Cl)(cod)}_2_] (C4) showed poor activity at ambient temperature (Table 1, entry 13) and 2A was found as the main product in the postreaction mixture. However, an increase in the reaction temperature resulted in the formation of a significant amount of the dehydrogenative silylation products (2C–2D), even if a slight excess of the olefin was used. It should be strongly underlined that further increase in the olefin amount in the reaction system caused a shift of equilibrium towards the formation of unsaturated silyl products, thereby ensuring the complete conversion of 1 as well as the exclusive formation of the mono-dehydrogenative silylated product 2C. Moreover, detailed NMR analysis of the isolated compounds revealed that the established conditions led to the formation of target molecules with very high regioselectivities. It is noteworthy that the Pt-based complex enabled the selective formation of β-isomeric product. However, the distribution of resonance lines in the olefin region in the ^1^H NMR spectrum of the isolated 2C compound clearly indicated the existence of two isomers. Nevertheless, the vast predominance of the β-*E* product was observed (β-*E*/β-*Z* = 95/5). Besides, the complete transformation of 1 into the target compounds was also confirmed by the ^29^Si NMR analysis. The NMR experiments showed the complete disappearance of the peak located at −6.78 ppm, originating from H-SiMe_2_-moiety in 1, and the appearance of new signals shifted to lower field. However, the shift for 2A was more significant than for 2C. Exemplary ^1^H NMR spectra are presented in Fig. 1, while the ^29^Si spectra are given in Fig. 2.

**Scheme 2:**
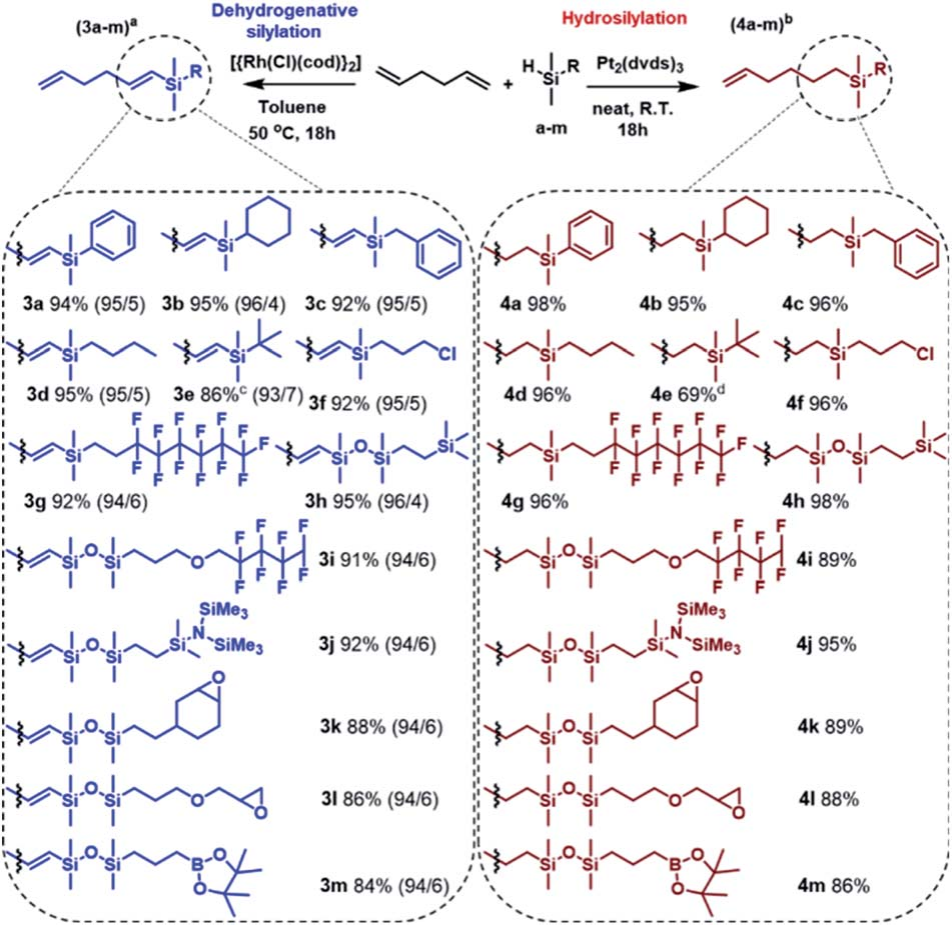
Main products distribution during functionalization of 1,5-hexadiene with pentamethyldisiloxane. Conditions: ^*a*^ 1.68 mmol of hydrosilane a–m, 8.4 mmol of 1,5-hexadiene, 2 mL of PhMe, [Rh] : [HSi] = 2 × 10^−4^ : 1, ^*b*^ 1.68 mmol of hydrosilane a–m, 16.8 mmol of 1,5-hexadiene, [Pt] : [HSi] = 2 × 10^−5^ : 1, ^*c*^ conversion of silane e at 80 °C, ^*d*^ [Pt] : [HSi] = 4 × 10^−5^ : 1. The ratio of β-*E* to β-*Z* for 3a–3m products is given in parentheses. Isolation yields.

Having optimized the conditions, the silylation of 1,5-hexadiene with a wide range of functional silanes and disiloxanes was carried out in two different manners, namely in the presence of the Pt-Karstedt's complex or the rhodium catalyst. It resulted in the formation of a family of monosubstituted 1,5-hexadiene derivatives containing sp^3^- or sp^2^-hybridized carbon atoms directly bonded to the silicon atom (Scheme 2).

The established conditions seem to be versatile because the products distribution was similar to that observed for the model reaction, regardless of the type of functional group present in the silicon modifiers (a–m). However, only for dimethyl(*tert*-butyl)silane (3e, 4e) the reactions did not run as expected. Considering the structure of this compound, it can be explained by the steric hindrance resulting from the presence of the *tert*-butyl substituent. In this specific case, Karstedt's complex enabled only 36% conversion of the silane, so the second portion of the catalyst was added and the reaction was continued for another 18 hours, which resulted in the increase in the silane conversion up to 76%. NMR and GC-MS analyses revealed the formation of the hydrosilylation product only, nevertheless, a significant amount of by-product (9%) was also formed as a result of olefin isomerization (Fig. S55 in ESI†). On the other hand, analysis of the postreaction mixture obtained after completion of the Rh-catalyzed process revealed the lack of the expected product, which should be formed under predetermined conditions. Moreover, the introduction of another portion the catalyst to the reaction system caused no significant change in the conversion of silane. Ultimately, an increase in temperature up to 80 °C resulted in 86% consumption of organosilicon derivative. It should be strongly emphasized that the analysis of the postreaction mixture revealed the presence of a significant amount of undesired disubstituted dehydrogenative silylation product (10%) as well as hydrosilylation products (8%). Thus, the selectivity of 3e formation was lower than that observed for the other products, which means that a high temperature has a very unfavorable influence on the regio- and chemoselectivity of the reaction. Apart from the above-described example, the optimized reaction conditions ensured regioselective and high yield synthesis of a wide range of novel alkenylsilanes equipped with inert or reactive functional groups, which may enable further modification or provide specific physical properties. It is worth noting that the functionalization of terminal dienes with hydrosilanes may also lead to hydrosilylation and subsequent cyclization, which results in the formation of functionalized carbocycles. This unusual pathway has been reported for the transformation of 1,6- and 1,7-dienes and allowed the synthesis of five and six-membered substituted carbocycles, respectively.^40,41^ With regard to the above, the use of 1,5-hexadiene should lead to formation of the substituted cyclobutane derivatives, which is not thermodynamically privileged. Therefore, no cycloalkane-based products were found in the postreaction mixtures. Mild conditions, low metal loading, and simple isolation procedure make the presented approach highly efficient. It should be also emphasized that the advantage of the described synthetic method is no need for the use of inert gas, special purification of the reagents, and the use of sophisticated procedures for preparation and/or activation of catalysts. The catalytic systems employed in this work are well-defined, effective and commercially available, which significantly simplifies the whole synthetic procedure.

In view of the literature data, the number of reports describing the attempts for the selective silylation of dienes is scarce.^42,43^ Saiki studied Pt-catalysed hydrosilylation of dienes and discovered that the presence of polar additives (ethers) significantly influence the regioselectivity of the reaction. However, the substrate scope was only limited to chlorosilanes.^42^ On the other hand, Murai and co-workers have reported RhCl(PPh_3_)_3_-catalysed dehydrogenative silylation of 1,5-dienes. Nevertheless, only trialkylsilanes (HSiMe_3_, HSiEt_2_Me) and triethoxysilane were applied as silylation agents. Moreover, the authors also observed the formation of by-products (up to 9%) as a result of isomerization reaction,^43^ which can be explained by high metal loading (25-fold higher Rh loading than presented in this work). It should be emphasized, that the proposed by us methodology enables direct access to new alkenylsilanes under milder conditions. Furthermore, scope of functional groups used for functionalization of 1,5-hexadiene has been significantly increased. Therefore, the synthesized compounds can be considered as useful building blocks in the preparation of silicon-containing hybrid materials recently showing raised attention.^44–46^ The detailed experimental procedures as well as NMR spectra of all products are provided in the ESI.†

## Conclusions

In brief, we have reported here a synthetic protocol that allows efficient preparation of unique unsymmetrical organo-functional alkenylsilanes and siloxanes through a highly regioselective and chemoselective hydrosilylation and dehydrogenative silylation of 1,5-hexadiene. The employment of Pt(0) and Rh(i) catalysts enabled the synthesis of new organosilicon derivatives with good isolation yields (69–98%). Moreover, all synthesized compounds contain the most common organic groups as well as the terminal alkene moiety, which can be used in subsequent stoichiometric and catalytic transformations to create further silicon derivatives tailored to specific applications.

## Conflicts of interest

There are no conflicts to declare.

## Supplementary Material

RA-011-D1RA07468G-s001
