# Supplementary material for: A library of new bifunctional alkenes obtained by a highly regiodivergent silylation of 1,5-hexadiene
Source: RSC Adv. 2021 Dec 7;11(62):38956–60. doi: 10.1039/d1ra07468g (PMC9044431; doi:10.1039/d1ra07468g)
Supplement: RA-011-D1RA07468G-s001 [file RA-011-D1RA07468G-s001.pdf]

Electronic Supplementary Material (ESI) for RSC Advances.
This journal is © The Royal Society of Chemistry 2021

SUPPORTING INFORMATION

FOR

A library of new bifunctional alkenes obtained by a highly regiodivergent silylation of 1,5-hexadiene

Rafał Januszewski* [1,2], Bartosz Orwat [1,3], Jan Merna [4], Ireneusz Kownacki* [1,2]

[1] Faculty of Chemistry, Adam Mickiewicz University in Poznan, Uniwersytetu Poznanskiego 8, 61-614, Poznan, Poland

[2] Center for Advanced Technology, Adam Mickiewicz University in Poznan, Uniwersytetu Poznanskiego 10, 61-614, Poznan, Poland

[3] Department of Molecular Physics, Lodz University of Technology, 90-924 Lodz, Zeromskiego 116, Poland

[4] University of Chemistry and Technology in Prague, Technická 5, 166 28, Prague 6, Czech Republic

Correspondence:

*Rafał Januszewski e-mail: r.janusz@amu.edu.pl

*Ireneusz Kownacki e-mail: ireneusz.kownacki@amu.edu.pl

Table of contents

1. General remarks ... 3
1.1. Methods ... 3
1.2. Materials ... 3
1.3. General procedures for functionalization of 1,5-hexadiene ... 3
2. Analytical data and NMR spectra of isolated products ... 4
2.1. Product 1A ... 4
2.2. Product 1C ... 7
2.3. Product 2a ... 10
2.4. Product 2b ... 13
2.5. Product 2c ... 16
2.6. Product 2d ... 19
2.7. Product 2f ... 22
2.8. Product 2g ... 25
2.9. Product 2h ... 28
2.10. Product 2i ... 31
2.11. Product 2j ... 34
2.12. Product 2k ... 37
2.13. Product 2l ... 40
2.14. Product 2m ... 43
2.15. Product 3a ... 46
2.16. Product 3b ... 49
2.17. Product 3c ... 52
2.18. Product 3d ... 55
2.19. Product 3e ... 58
2.20. Product 3f ... 61
2.21. Product 3g ... 64
2.22. Product 3h ... 67
2.23. Product 3i ... 70
2.24. Product 3j ... 73
2.25. Product 3k ... 76
2.26. Product 3l ... 79
2.27. Product 3m ... 82

1. General remarks

1.1. Methods

NMR analysis - ^{1}H, ^{13}C and ^{29}Si NMR spectra were recorded at 25 °C on Bruker UltraShield 300 or 400 MHz. Chemical shifts were reported in ppm with the reference to the residue portion solvent peak. In all experiments d^{1}-chloroform was used as a solvent.

GC-MS analysis - The mass spectrum of the products were obtained by GC-MS analysis on a Bruker Scion 436-GC with a 30m Varian DB-5 0.25mm capillary column and a Scion SQ-MS mass spectrometry detector. Two temperature programs were used a) 60 °C (3 min), 10°C/min, 250 °C (30 min), b) 100 °C (3 min), 10°C/min, 280 °C (44.5 min).

1.2. Materials

1,5-hexadiene, 1,1,3,3-tetramethyldisiloxane, dimethylphenylsilane, anhydrous toluene, dimethylcyclohexylsilane, dimethyl(*tert*-butyl)silane, vinyltrimethylsilane, Karstedt's complex were purchased from Sigma-Aldrich. Platinum-octanal/octanol complex (2-2.5% Platinum concentration, dimethylbenzylsialne, dimethylbutylsilane were obtained from Fluorochem. 1,1,3,3,5-pentamethyldisiloxane, H_2PtCl_6, Rhodium (III) chloride were purchased from ABCR. Unsymmetrical disiloxanes were synthesized with the use of previously reported procedures.[1-3] 3-chloroprpyldimethylsialne was prepared by method published by Daiss and co-workers.[4] The [{Rh(μ-Cl)(cod)}$_2$] was prepared according to published method.[5]

[1]R. Januszewski, I. Kownacki, H. Maciejewski, B. Marciniec, A. Szymanska *European Journal of Inorganic Chemistry* **2017**, 851-856.

[2]R. Januszewski, I. Kownacki, H. Maciejewski, B. Marciniec, *Journal of Organometallic Chemistry* **2017**, 846, 263-268.

[3]R. Januszewski, M. Grzelak, B. Orwat, M. Dutkiewicz, I. Kownacki, *Journal of Catalysis* **2020**, 390, 103-108.

[4]J. O. Daiss, S. Duda-Johner, C. Burschka, U. Holzgrabe, K. Mohr and R. Tacke, *Organometallics,* **2002**, 21, 803–811.

[5]S. Komiya, Synthesis of Organometallic Compounds: A Practical Guide (Ed. S. Komiya), Wiley, New York, 1997, p. 442.

1.3. General procedures for functionalization of 1,5-hexadiene

Hydrosilylation of 1,5-hexadiene: 1.68 mmol of silane and 16.8 mmol of 1,5-hexadiene were placed in a glass reactor and stirred at room temperature. To prepared mixture Karstedt's complex was added ([Pt] : [HSi] = $2x10^{-5}$: 1). The reaction was monitored with GC analysis. After the complete conversion of the organosilicon compound the mixture was filtered through silica to separate the catalyst and was washed with diethyl ether or hexane. Evaporation of the solvent and 1,5-hexadiene excess gave the product as a pale yellow or transparent liquid.

Dehydrogenative silylation of 1,5-hexadiene: 1.68 mmol of silane and 8.4 mmol of 1,5-hexadiene were placed in a glass reactor and dissolved in 2mL of toluene. Prepared mixture was heated up to 50 °C, then the catalyst [$2x10^{-4}$ Rh/ per 1 mol of H-Si] [{Rh(μ-Cl)(cod)}$_2$]

was added. The reaction was monitored with GC analysis. After the complete conversion of silane the mixture was then filtered through silica to separate the catalyst and was washed with diethyl ether or hexane. Evaporation of the solvent and 1,5-hexadiene excess gave the product as a pale yellow or transparent liquid.

2. Analytical data and NMR spectra of isolated products

2.1. Product 2A

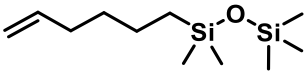

^{1}H NMR (300 MHz, $CDCl_3$) δ 5.81 CH_2=<u>CH</u>R (ddt, *J* = 16.9, 10.2, 6.7 Hz, 1H), 5.09 – 4.87 <u>CH_2</u>=CHR (m, 2H), 2.05 (q, *J* = 6.8 Hz, 2H), 1.36 (m, 4H), 0.60 – 0.43 CH_2Si (m, 2H), 0.06 $SiMe_3$ (s, 9H), 0.04 $SiMe_2$ (s, 6H).
^{13}C NMR (75 MHz, $CDCl_3$) δ 139.30, 114.28, 33.71, 32.74, 22.94, 18.35, 2.12, 0.49.
^{29}Si NMR (79 MHz, $CDCl_3$) δ 7.50, 7.07.
MS (EI, m/z): 215.0 $[M-15]^+$ (3.5), 149.0 (7.5), 148.1 (14.7), 146.9 (100), 132.9 (44.5), 72.9 (14.1),
Product contains 2% of disubstituted derivative

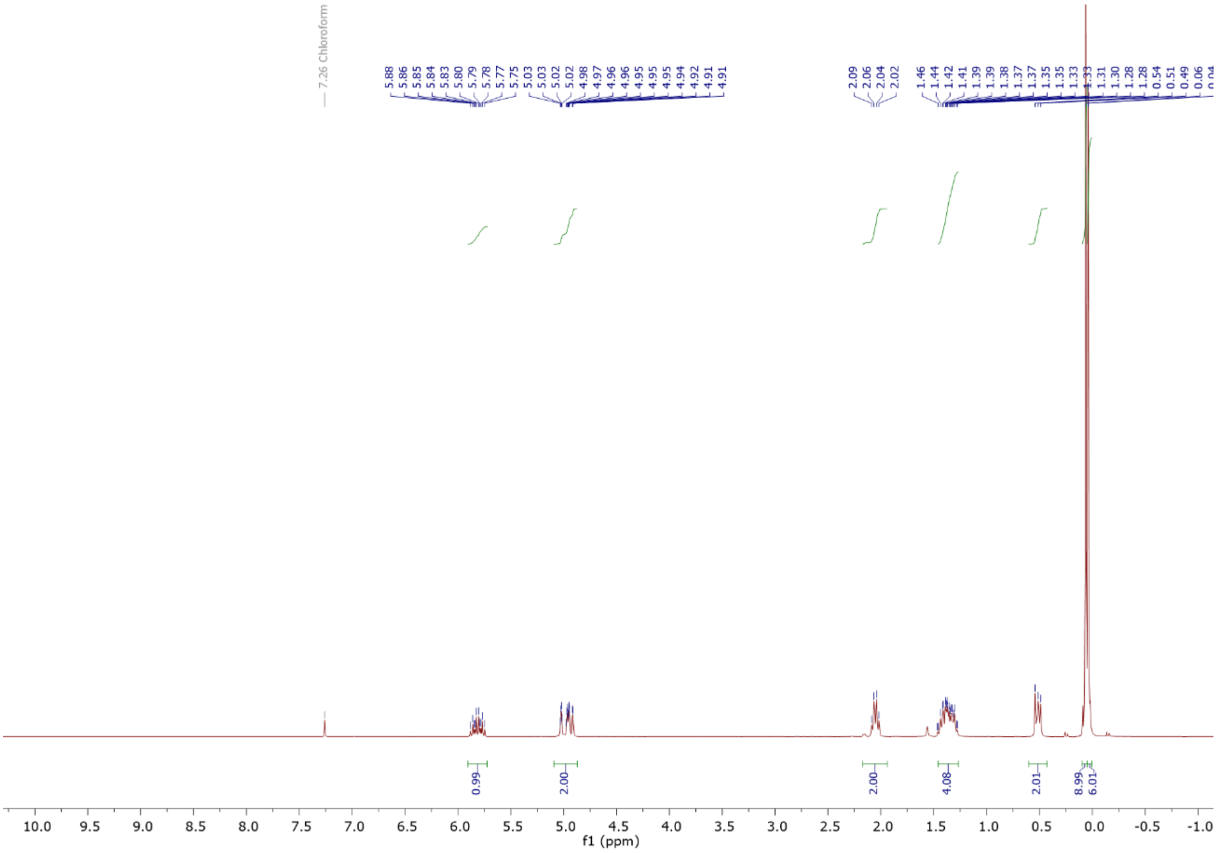

Figure S1. ^{1}H NMR spectrum of 2A

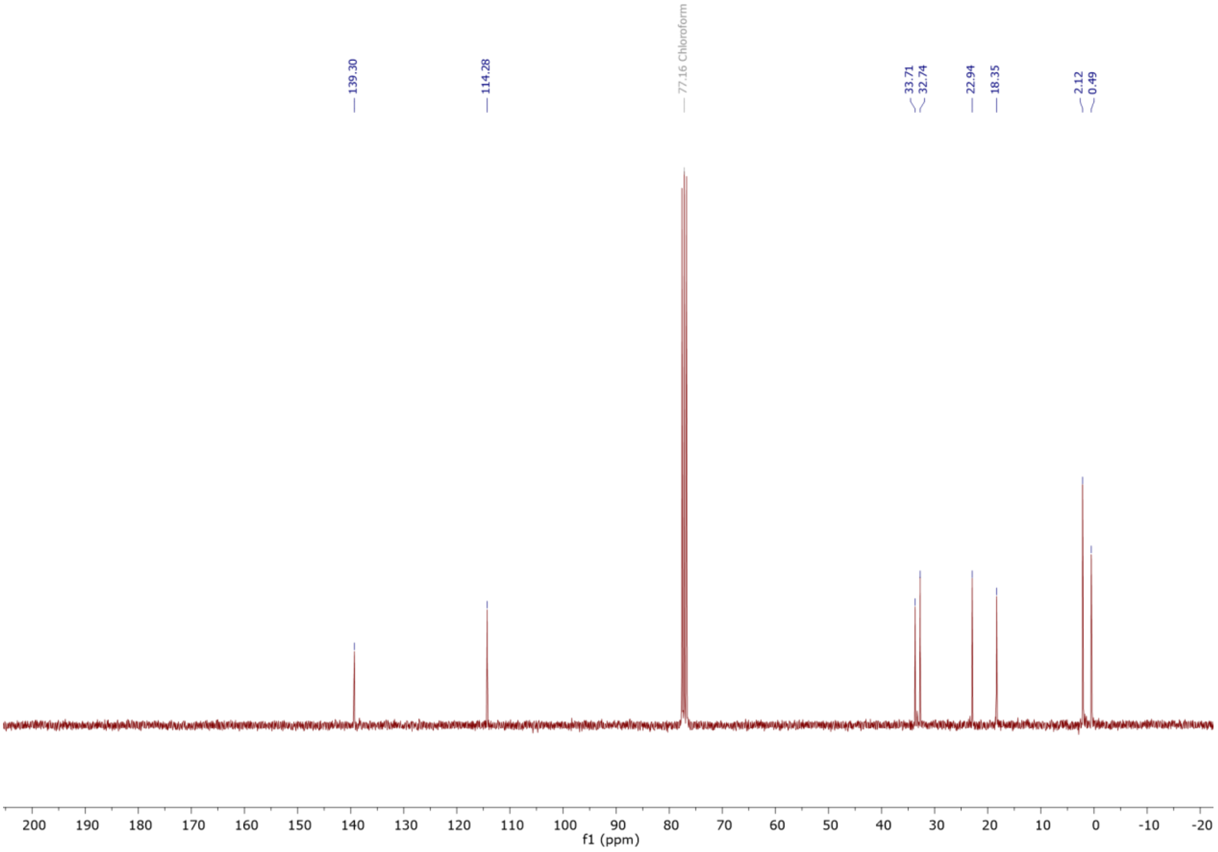

Figure S2. ^{13}C NMR spectrum of 2A

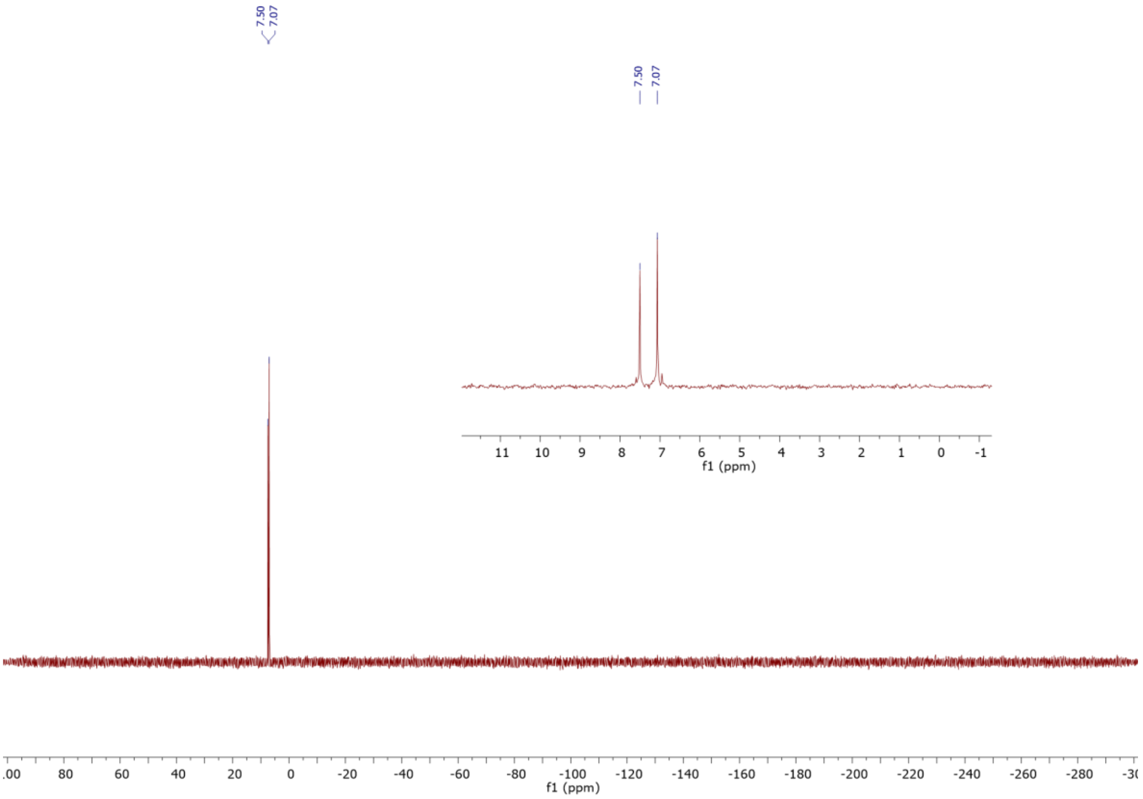

Figure S3. ^{29}Si NMR spectrum of 2A

2.2. Product 2C

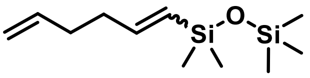

^{1}H NMR (300 MHz, $CDCl_3$) δ, 6.10 RCH=CHSi (dt, J = 18.6, 5.8 Hz, 1H), 5.94 – 5.72 CH_2=CHR (m, 1H), 5.63 RCH=CHSi (d, J = 18.7 Hz, 1H), 5.13 – 4.79 CH_2=CHR (m, 2H), 2.19 (m, 4H), 0.11 $SiMe_2$ (s, 6H), 0.07 $SiMe_3$ (s, 9H). Isomer *β-Z*: 6.30 RCH=CHSi (dt, J = 14.3, 7.2 Hz, H) 5.47 RCH=CHSi (d, J = 14.2 Hz).
^{13}C NMR (75 MHz, $CDCl_3$) δ 147.12, 138.36, 130.11, 114.81, 35.87, 32.94, 2.16, 0.91.
^{29}Si NMR (79 MHz, $CDCl_3$) δ 7.91, -4.10.
MS (EI, m/z): 213.0 [M-15]$^+$ (3.9), 156.9 (5.6), 148.1 (6.5), 146.9 (44.6), 135.0 (7.1), 134.1 (13.6), 132.9 (100), 116.9 (5.2), 72.9 (15.0), 58.9 (5.3)

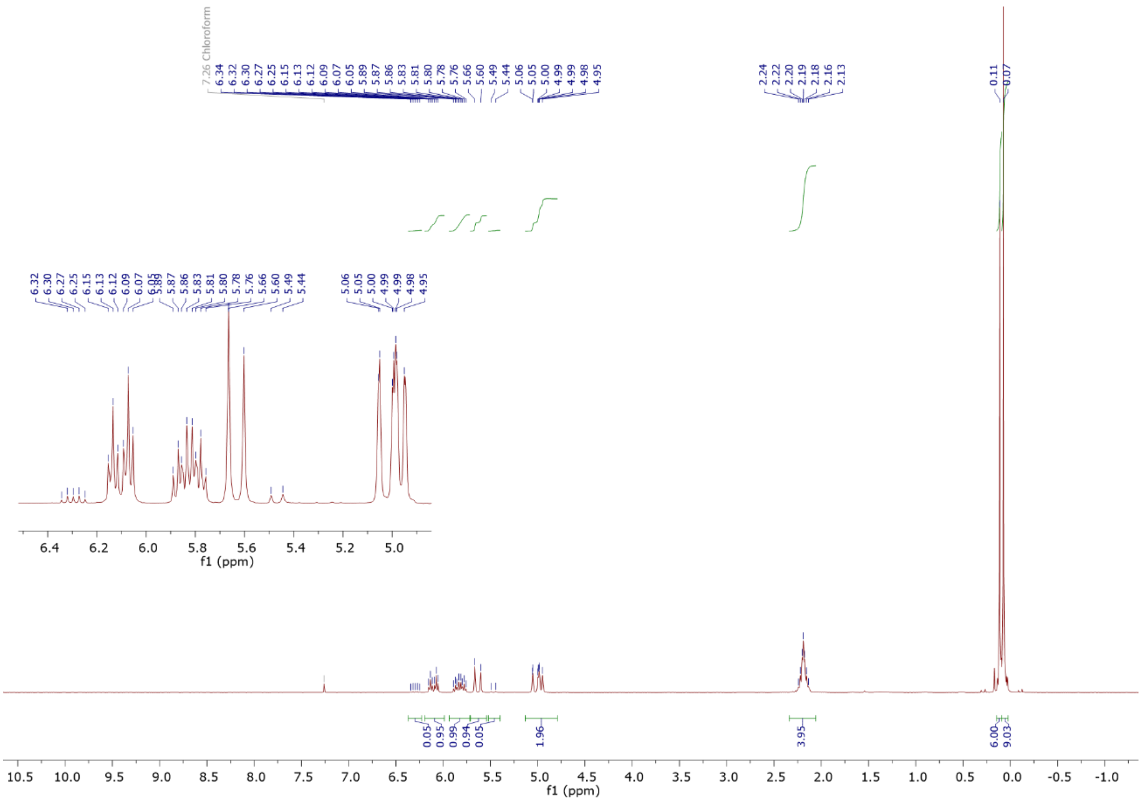

Figure S4. ^{1}H NMR spectrum of 2C

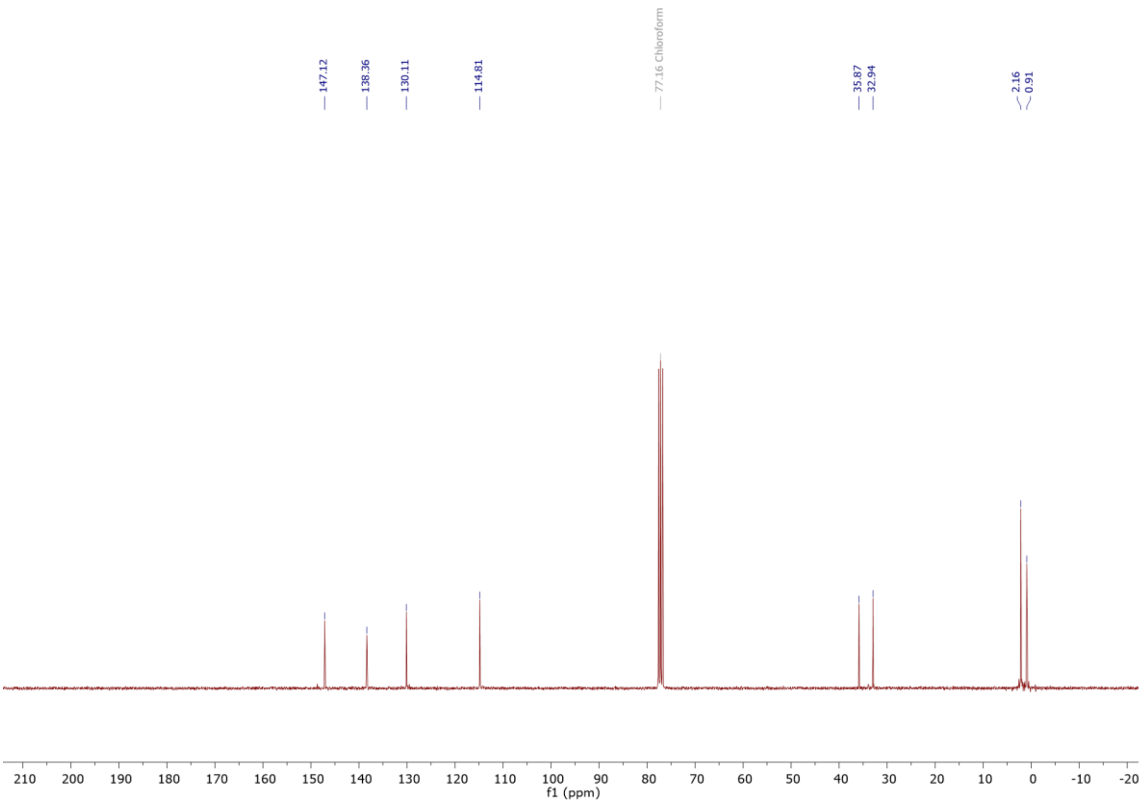

Figure S5. ^{13}C NMR spectrum of 2C

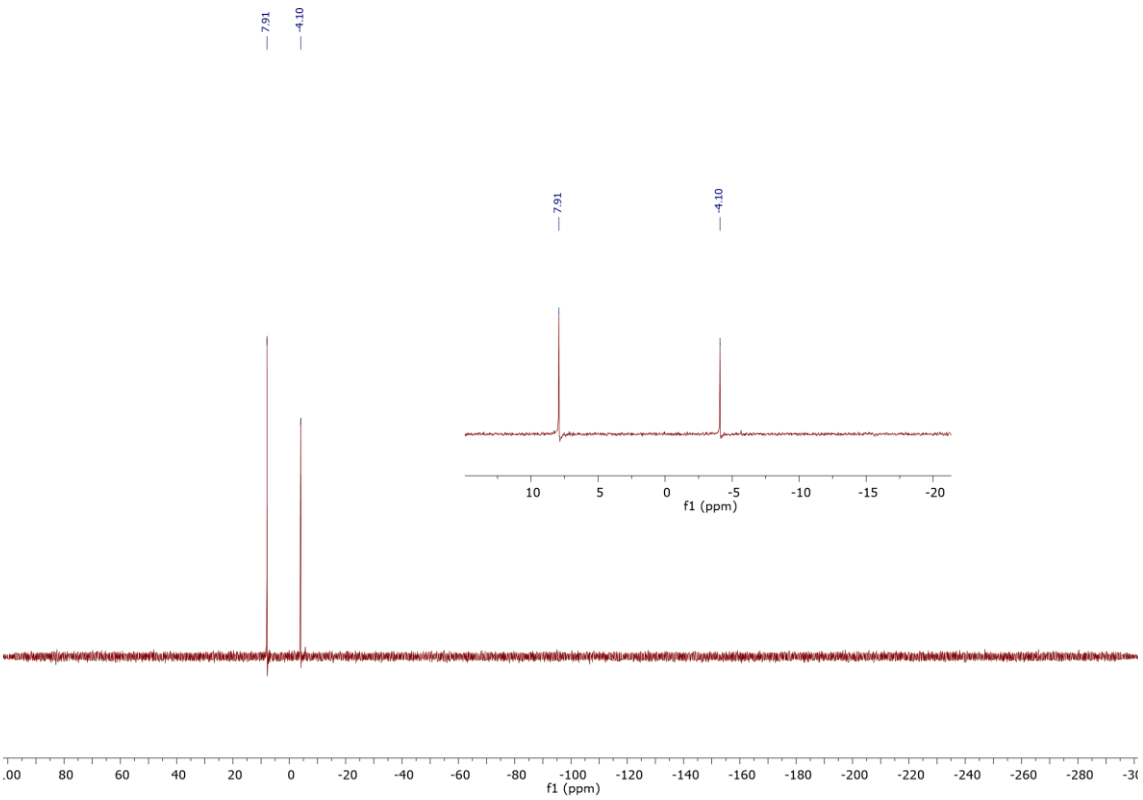

Figure S6. ^{29}Si NMR spectrum of 2C

2.3. Product 3a

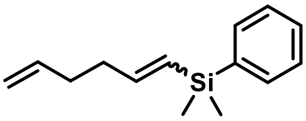

^{1}H NMR (300 MHz, $CDCl_3$) δ 7.59 – 7.50 (m, 2H), 7.40 – 7.32 (m, 3H), 6.14 RCH=CHSi (dt, J = 18.6, 5.8 Hz, 1H), 5.92 – 5.73 CH_2=CHR, RCH=CHSi (m, 2H), 5.13 – 4.89 CH_2=CHR (m, 2H), 2.23 (m, 4H), 0.34 $SiMe_2$ (s, 6H), Isomer β-Z: 6.44 RCH=CHSi (dt, J = 14.1, 7.1 Hz), 5.69 RCH=CHSi (d, J = 14.9 Hz).
^{13}C NMR (75 MHz, $CDCl_3$) δ 148.44, 139.36, 138.32, 133.97, 128.97, 128.03, 127.85, 114.85, 36.14, 32.99, -2.31.
^{29}Si NMR (79 MHz, $CDCl_3$) δ -11.92.
MS (EI, m/z): 216.1 M^+ (2.2), 201.0 $[M-15]^+$ (16.0), 175.0 (23.3), 173.0 (5.7), 158.9 (13.5), 144.9 (15.4), 137.9 (19.1), 136.1 (8.3), 134.9 (59.0), 130.9 (5.6), 123.1 (9.9), 122.1 (9.8), 120.9 (100), 104.8 (13.1), 98.9 (46.0), 94.9 (7.8), 80.1 (7.4)73.0 (11.1), 58.9 (29.5).

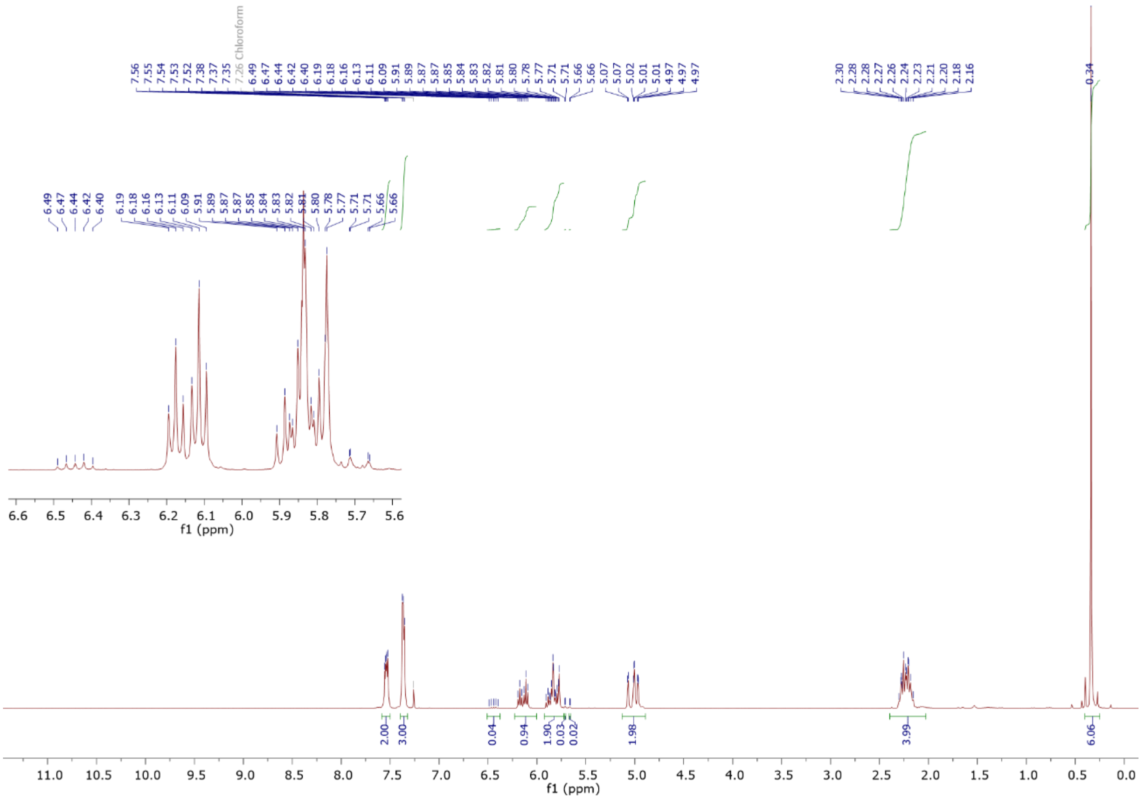

Figure S7. ^{1}H NMR spectrum of 3a

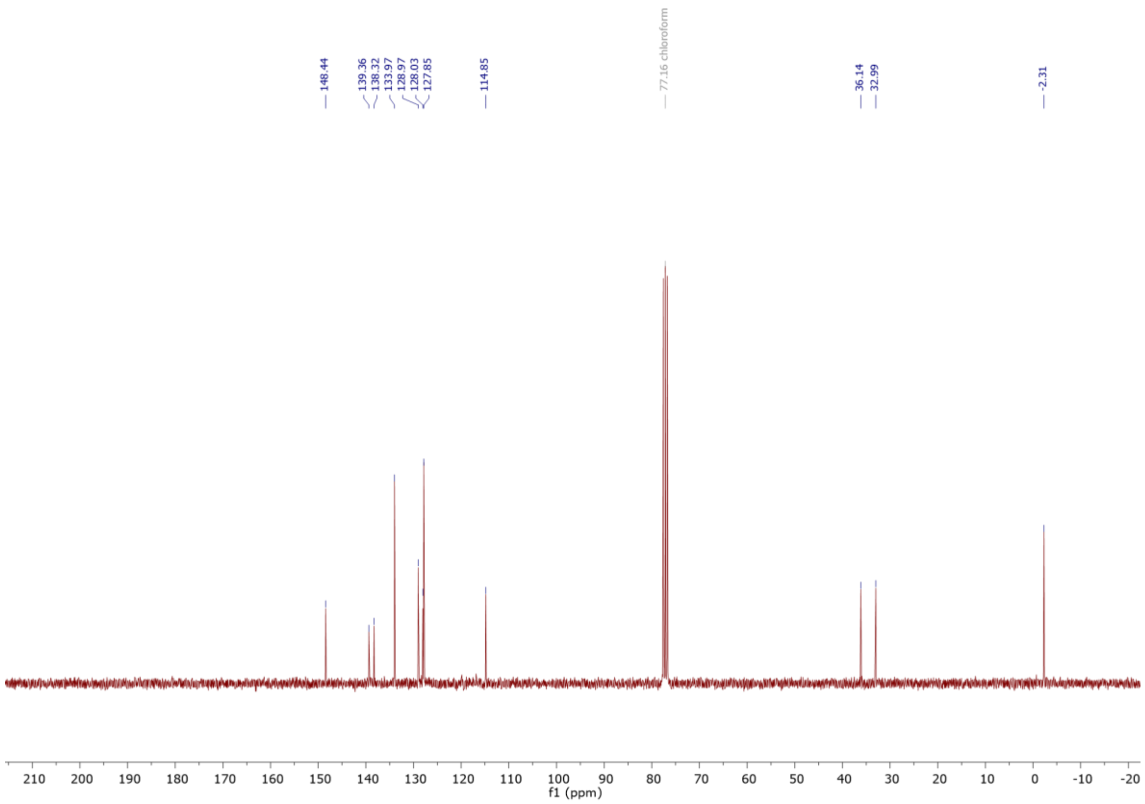

Figure S8. ^{13}C NMR spectrum of 3a

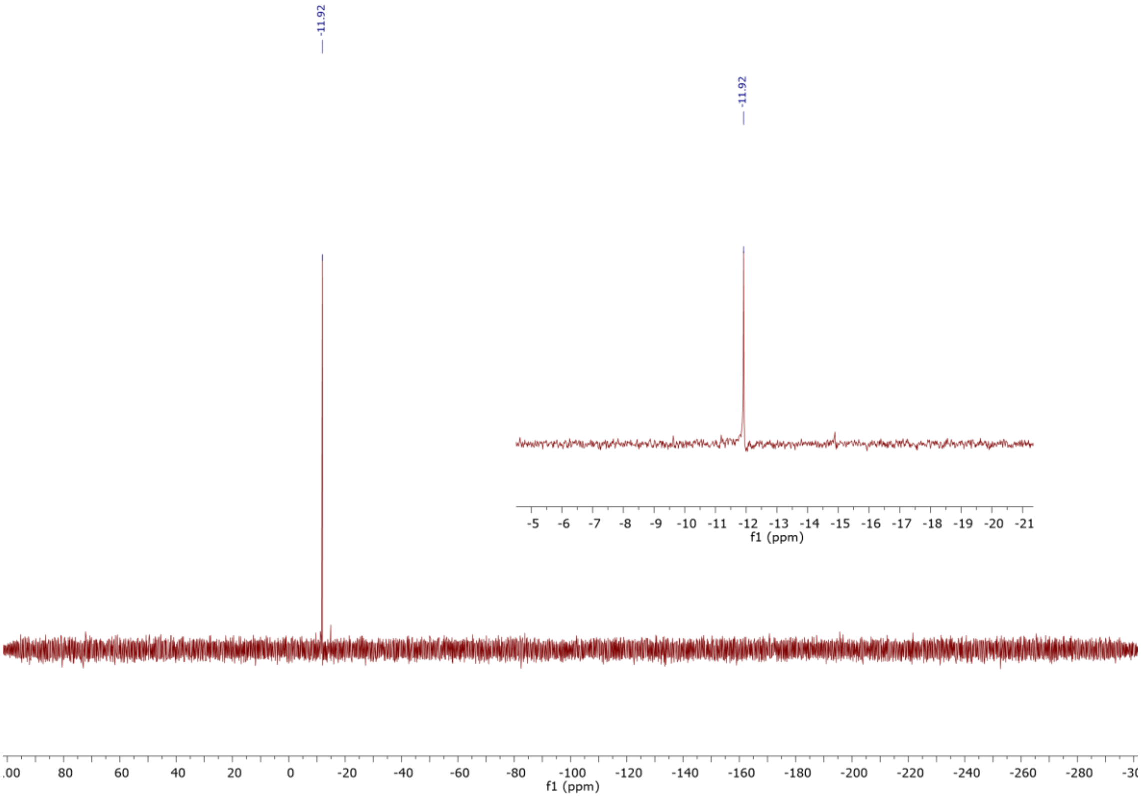

Figure S9. ^{29}Si NMR spectrum of 3a

2.4. Product 3b

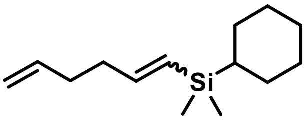

^{1}H NMR (300 MHz, $CDCl_3$) δ 6.01 R<u>CH</u>=CHSi (dt, *J* = 18.6, 5.8 Hz, 1H), 5.82 (ddt, *J* = 16.6, 10.4, 6.3 Hz, 1H), 5.61 RCH=<u>CH</u>Si (d, *J* = 18.7 Hz, 1H), 5.10 – 4.85 (m, 2H), 2.18 (m, 4H), 1.68 (m, 6H), 1.27 – 0.98 (m, 5H), 0.67 – 0.50 SiCH (m, 1H), -0.02 $SiMe_2$ (s, 6H). Isomer *β-Z*: 6.33 R<u>CH</u>=CHSi (dt, *J* = 13.9, 6.9 Hz,), 5.47 RCH=<u>CH</u>Si (d, *J* = 13.1 Hz).
^{13}C NMR (101 MHz, $CDCl_3$) δ 147.20, 138.45, 128.17, 114.72, 36.25, 33.18, 28.27, 27.60, 27.17, 25.91, -4.90.
^{29}Si NMR (79 MHz, $CDCl_3$) δ -5.79.
MS (EI, m/z): 207.0 $[M-15]^+$ (4.8), 141.0 (12.0), 140.1 (13.8), 138.9 (86.6), 110.9 (16.0), 97.0 (14.1), 84.9 (9.7), 82.9 (9.9), 79.0 (57.5), 73.0 (34.8), 60.1 (6.8), 58.9 (100)

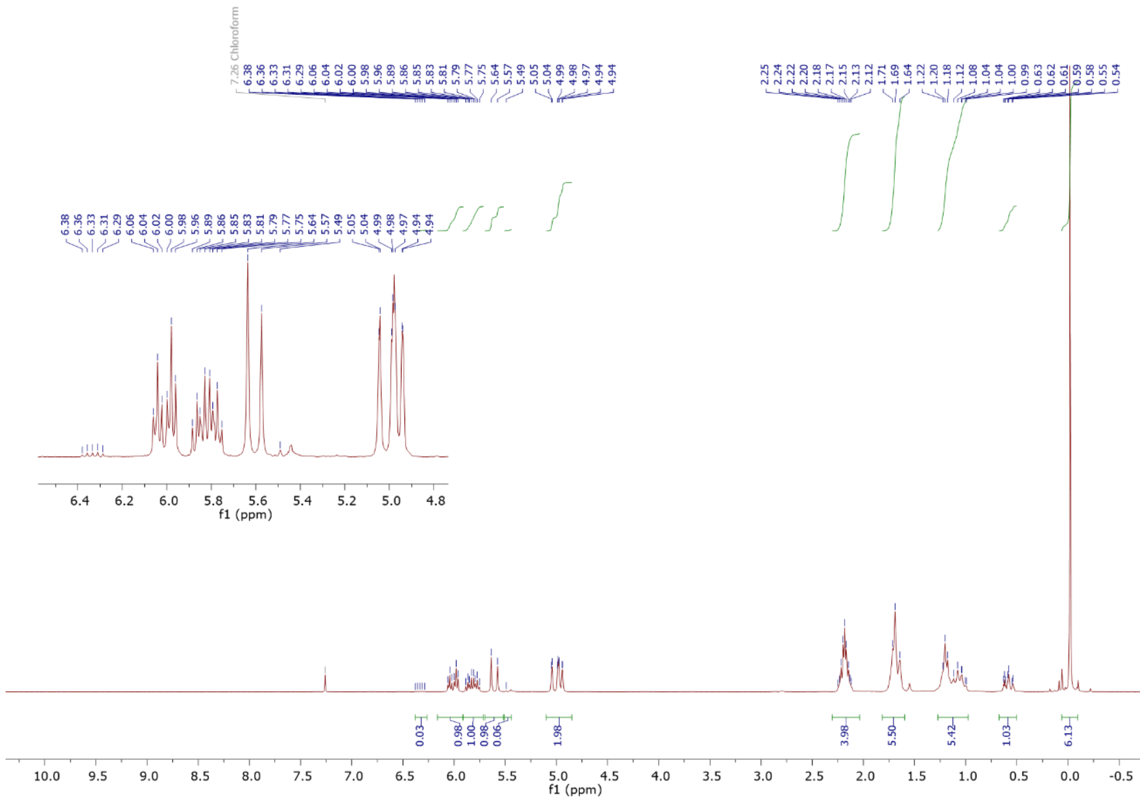

Figure S10. ^{1}H NMR spectrum of 3b

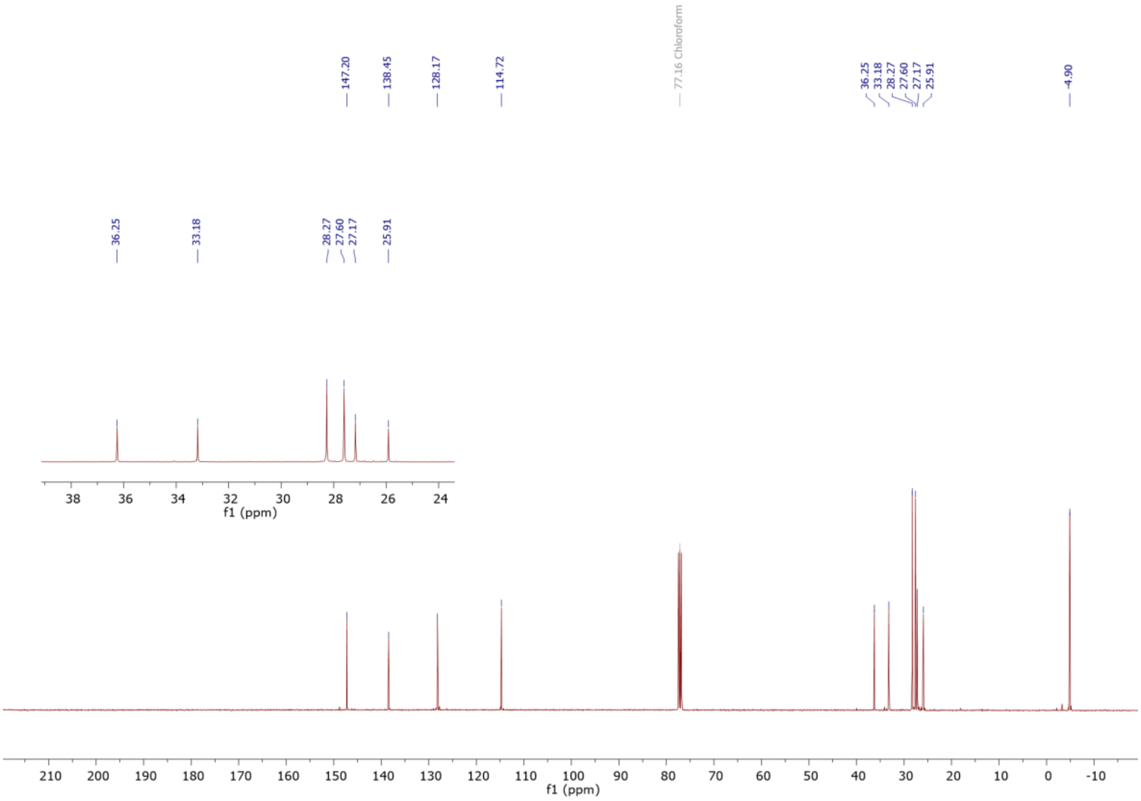

Figure S11. ^{13}C NMR spectrum of 3b

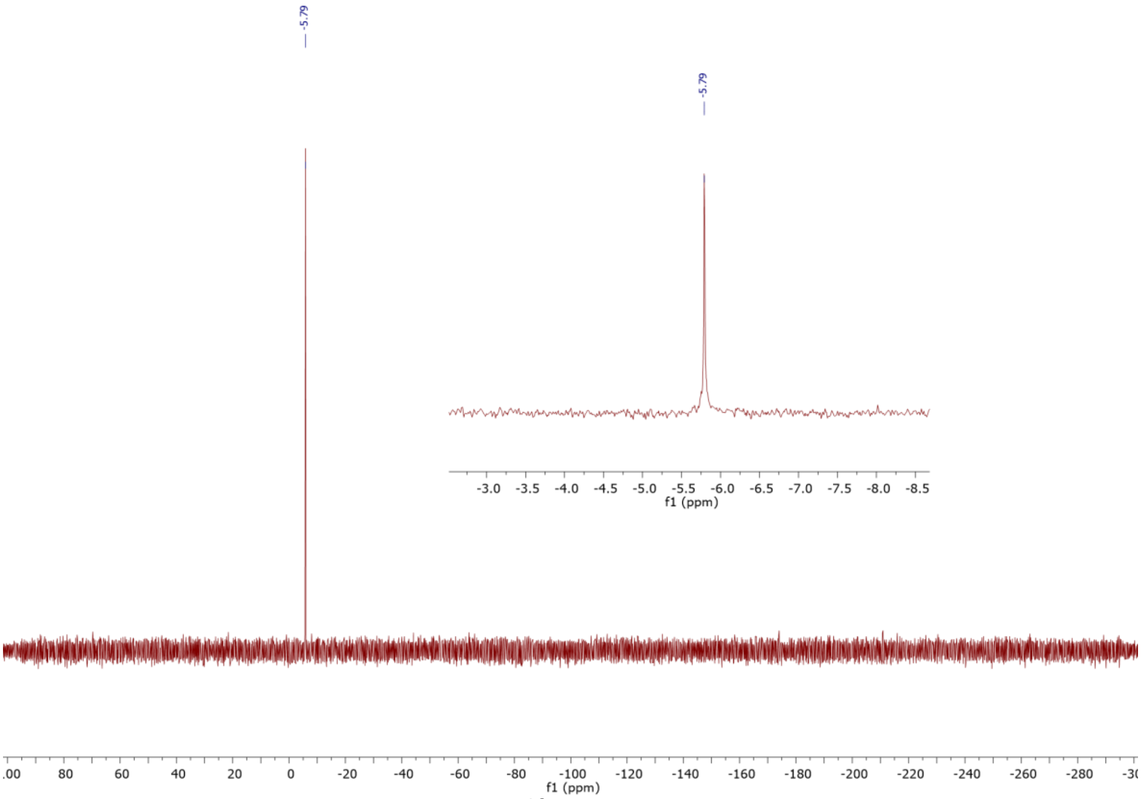

Figure S12. ^{29}Si NMR spectrum of 3b

2.5. Product 3c

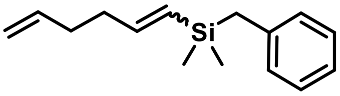

^{1}H NMR (300 MHz, $CDCl_3$) δ 7.21 (m, 2H), 7.05 (m, 3H), 6.02 RCH=CHSi (dt, J = 18.6, 5.8 Hz, 1H), 5.92 – 5.74 CH_2=CHR (m, 1H), 5.63 RCH=CHSi (d, J = 18.6 Hz, 1H), 5.12 – 4.90 CH_2=CHR (m, 2H), 2.30 – 2.02 (m, 6H), 0.04 $SiMe_2$ (s, 6H).
^{13}C NMR (75 MHz, $CDCl_3$) δ 147.71, 140.32, 138.34, 128.40, 128.24, 128.17, 124.01, 114.80, 36.13, 32.98, 26.37, -3.16. Isomer *β-Z*: 6.35 RCH=CHSi (dt, J = 14.0, 6.9 Hz), 5.49 (d, J = 14.0 Hz)
^{29}Si NMR (79 MHz, $CDCl_3$) δ -7.40.
MS (EI, m/z): 230.0 (2.7) M^+, 215.0 $[M-15]^+$ (2.4), 140.1 (10.4), 139.0 (78.4), 120.9 (5.9), 110.9 (14.4), 84.9 (9.2), 82.9 (10.3), 79.0 (47.3), 73.0 (26.6), 60.1 (6.9), 59.0 (100),

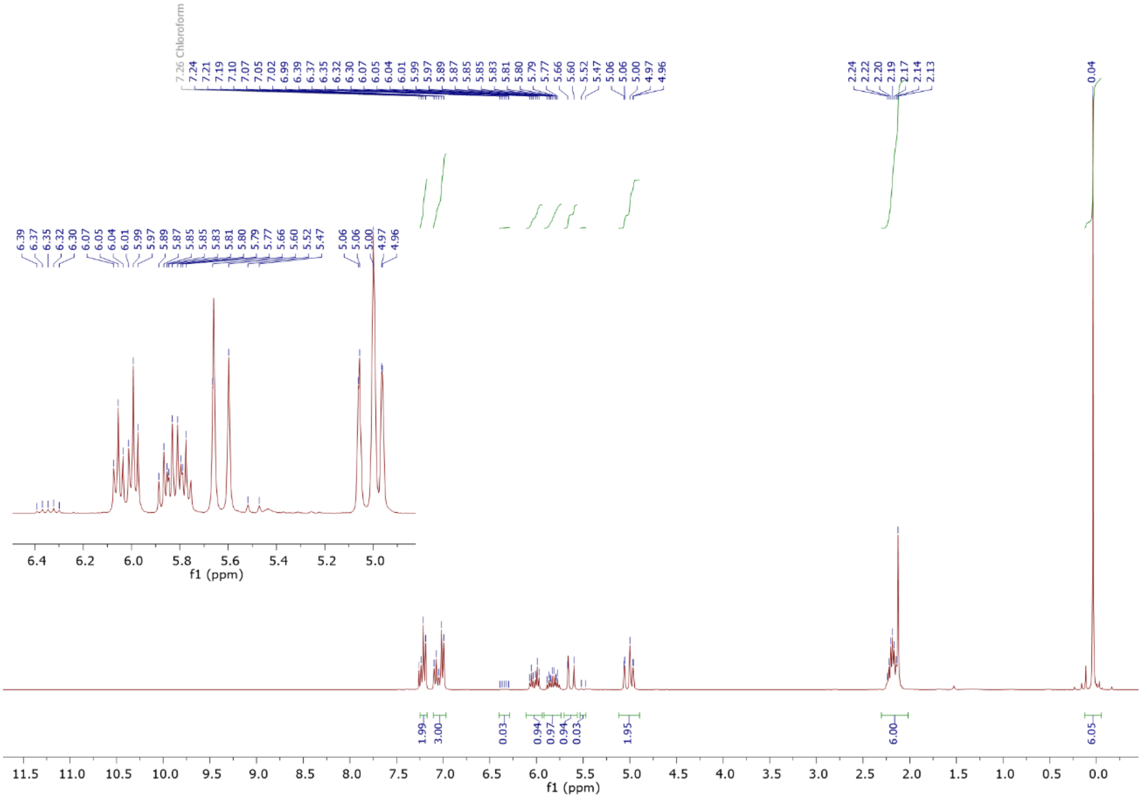

Figure S13. ^{1}H NMR spectrum of 3c

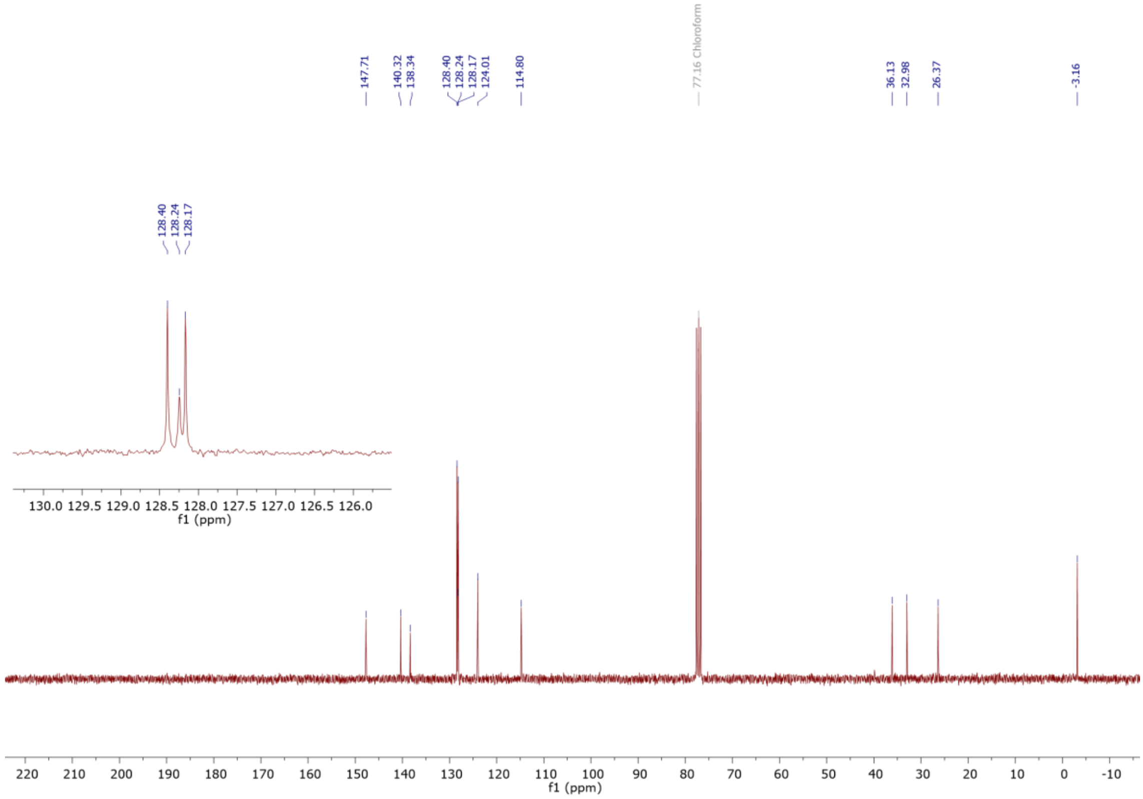

Figure S14. ^{13}C NMR spectrum of 3c

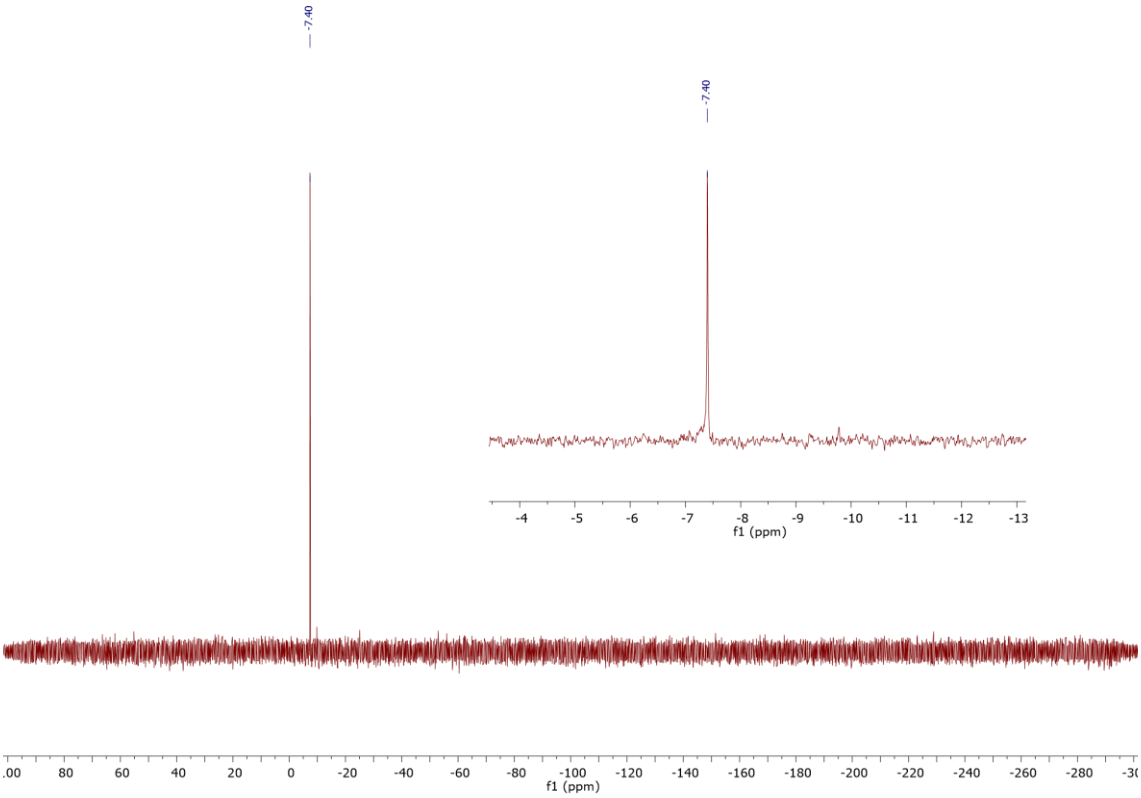

Figure S15. ^{29}Si NMR spectrum of 3c

2.6. Product 3d

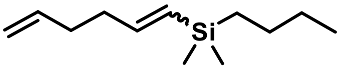

^{1}H NMR (400 MHz, $CDCl_3$) δ 6.02 R<u>CH</u>=CHSi (dt, J = 18.6, 5.8 Hz, 1H), 5.82 CH_2=<u>CH</u>R (ddt, J = 16.4, 10.3, 6.2 Hz, 1H), RCH=<u>CH</u>Si 5.63 (d, J = 18.6 Hz, 1H), 5.15 – 4.90 <u>CH_2</u>=CHR (m, 2H), 2.29 – 2.10 (m, 4H), 1.29 (m, 4H), 0.88 CH_3 (t, J = 7.0 Hz, 3H), 0.59 – 0.50 $SiCH_2$ (m, 2H), 0.02 $SiMe_2$ (s, 6H), Isomer *β-Z*: 6.31 R<u>CH</u>=CHSi (dt, J = 14.2, 7.1 Hz), 5.50 RCH=<u>CH</u>Si (d, J = 14.0 Hz)
^{13}C NMR (101 MHz, $CDCl_3$) δ 146.72, 138.44, 129.42, 114.72, 36.18, 33.13, 26.70, 26.29, 15.63, 13.97, -2.82.
^{29}Si NMR (79 MHz, $CDCl_3$) δ -6.84.
MS (EI, m/z): 181.0 $[M-15]^+$ (5.1), 140.2 (6.1), 139.0 (41.5), 124.9 (5.6), 114.9 (22.7), 110.9 (13.0), 99.2 (6.9), 96.9 (26.7), 82.9 (9.2), 80.1 (14.4), 79.0 (45.7), 73.0 (39.5), 70.9 (5.6), 60.1 (6.9), 59.0 (100).

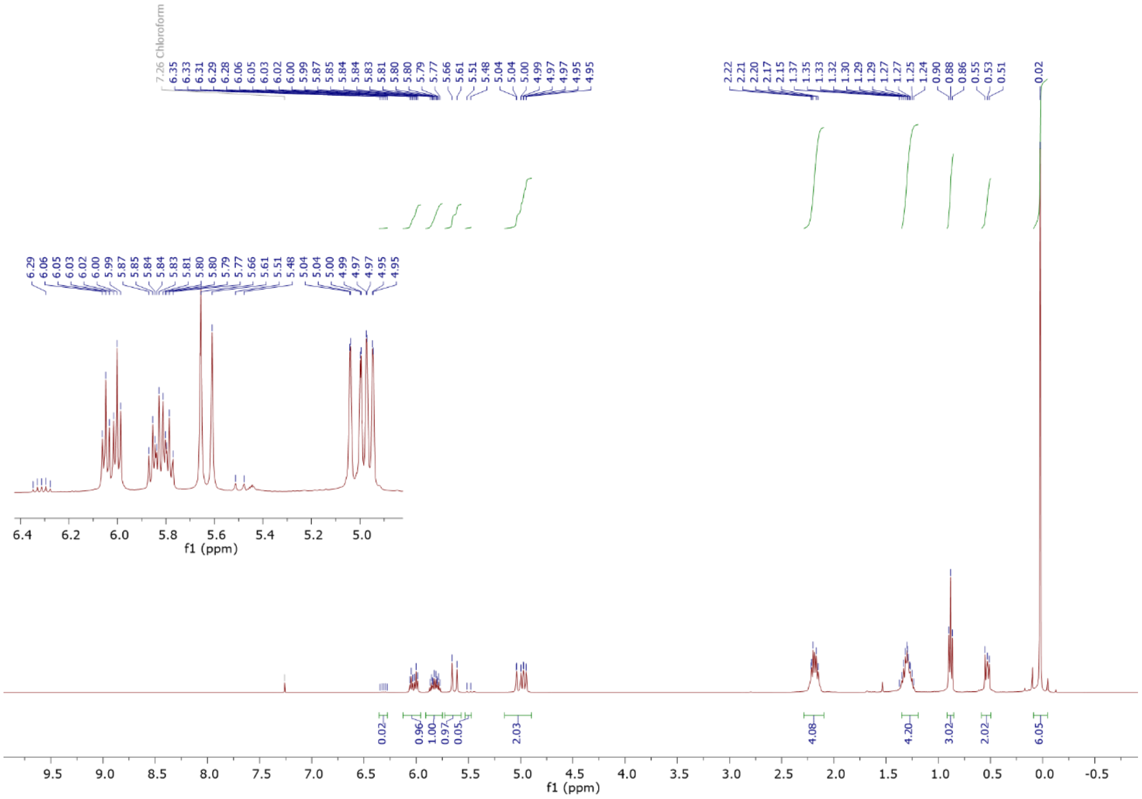

Figure S16. ^{1}H NMR spectrum of 3d

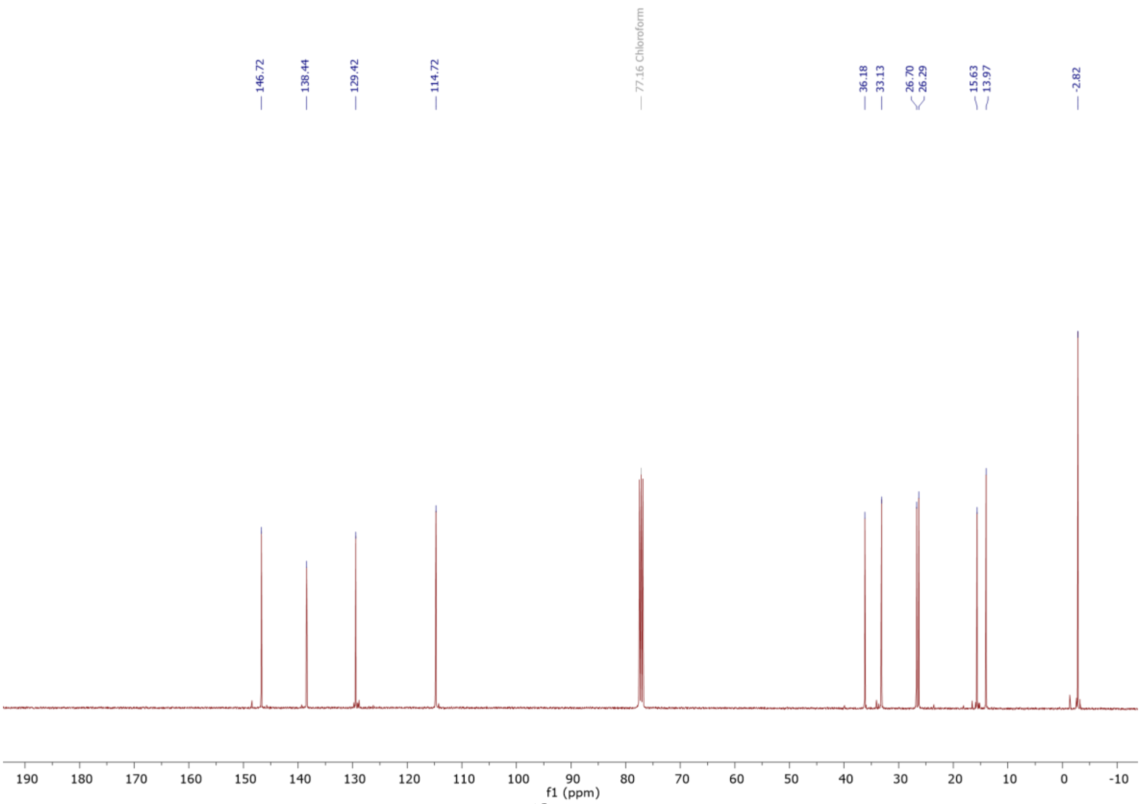

Figure S17. ^{13}C NMR spectrum of 3d

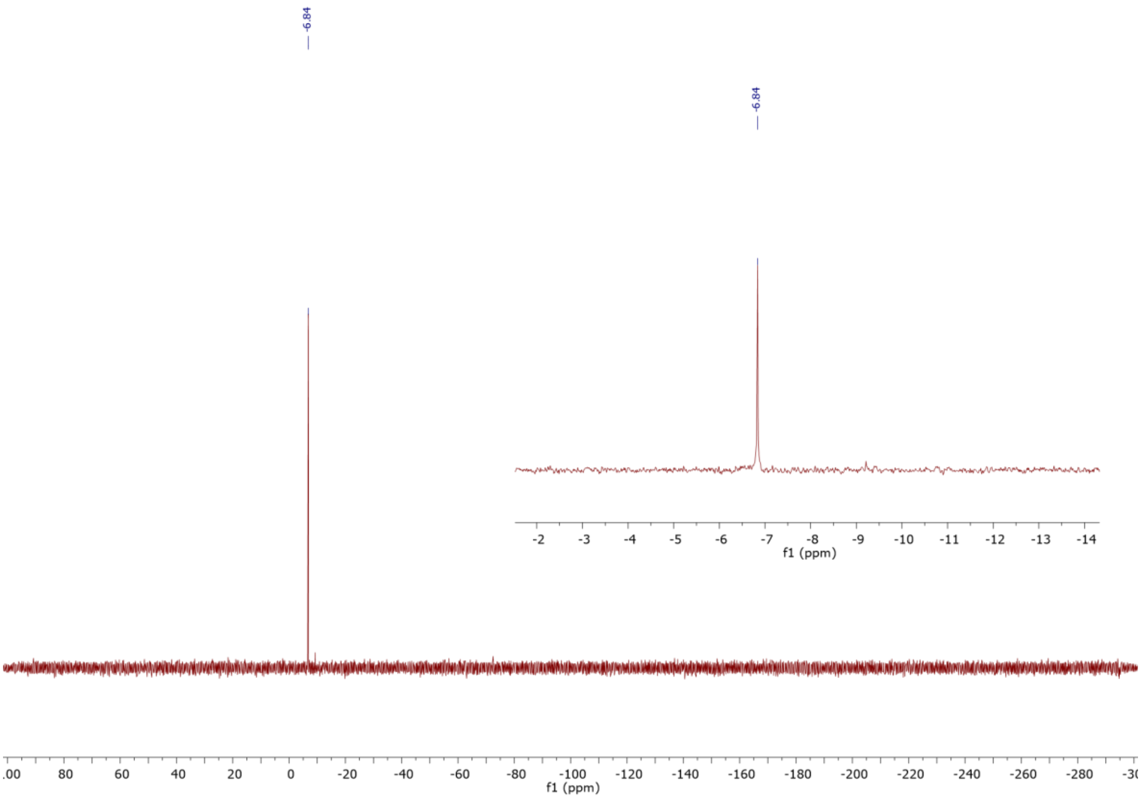

Figure S18. ^{29}Si NMR spectrum of 3d

2.7. Product 3f

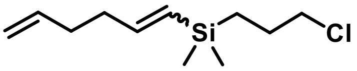

^{1}H NMR (300 MHz, $CDCl_3$) δ 6.04 RCH=CHSi (dt, *J* = 18.6, 5.8 Hz, 1H), 5.81 CH_2=CHR (ddt, *J* = 16.6, 10.3, 6.3 Hz, 1H), 5.61 RCH=CHSi (d, *J* = 18.6 Hz, 1H), 5.13 – 4.83 CH_2=CHR (m, 2H), 3.50 CH_2Cl (t, *J* = 7.0 Hz, 2H), 2.18 (m, 4H), 1.76 (m, 2H), 0.69 – 0.56 $SiCH_2$ (m, 2H), 0.05 $SiMe_2$ (s, 6H), Isomer *β-Z*: 6.34 RCH=CHSi (dt, *J* = 14.0, 7.0 Hz), 5.47 RCH=CHSi (d, *J* = 14.1 Hz).
^{13}C NMR (75 MHz, $CDCl_3$) δ 147.58, 138.28, 128.38, 114.85, 48.16, 36.12, 33.02, 27.84, 13.61, -2.97.
^{29}Si NMR (79 MHz, $CDCl_3$) δ -6.49.
MS (EI, m/z): 201.0 $[M-15]^+$ (5.6), 158.9 (21.4), 138.9 (17.2), 134.8 (6.6), 130.9 (5.2), 122.9 (17.1), 110.9 (11.0), 104.9 (8.6), 97.1 (9.3), 94.8 (42.1), 93.9 (5.9), 92.9 (100), 84.9 (7.4), 82.9 (6.9), 81.1 (13.4), 80.1 (44.7), 79.0 (70.4), 73.0 (20.6), 66.9 (5.6), 64.9 (6.3), 60.1 (6.7), 59.0 (86.7)

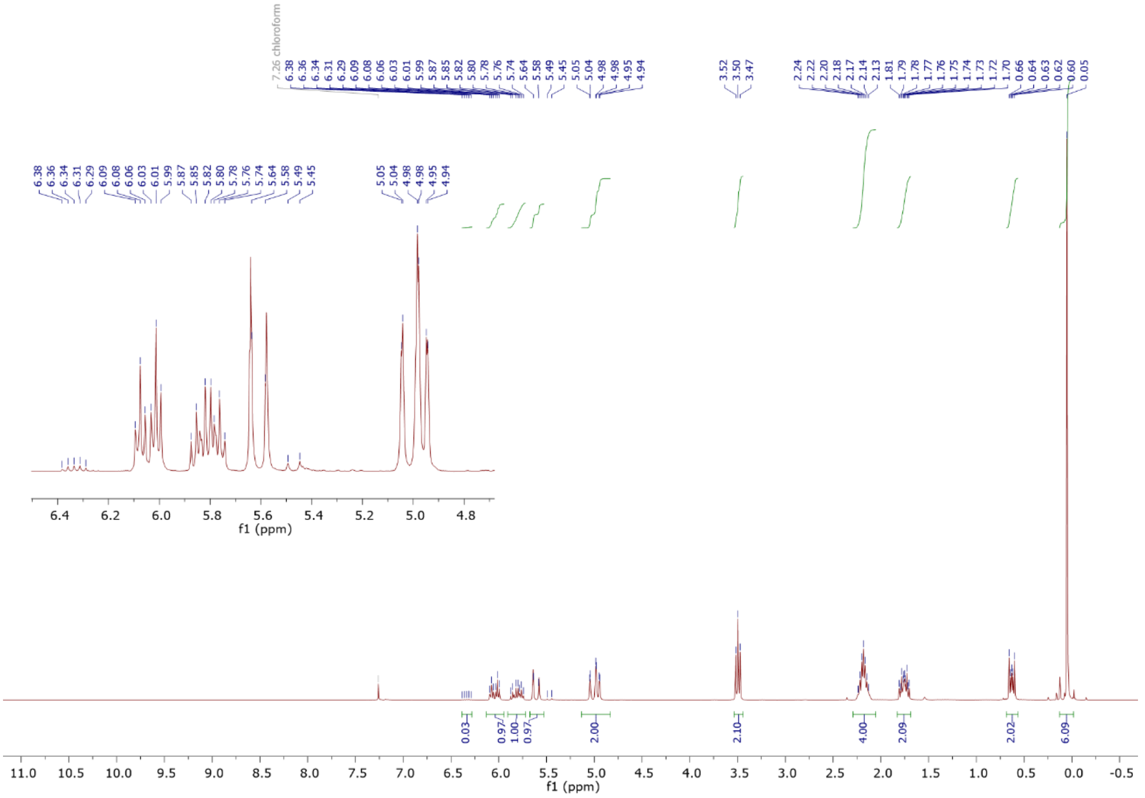

Figure S19. ^{1}H NMR spectrum of 3f

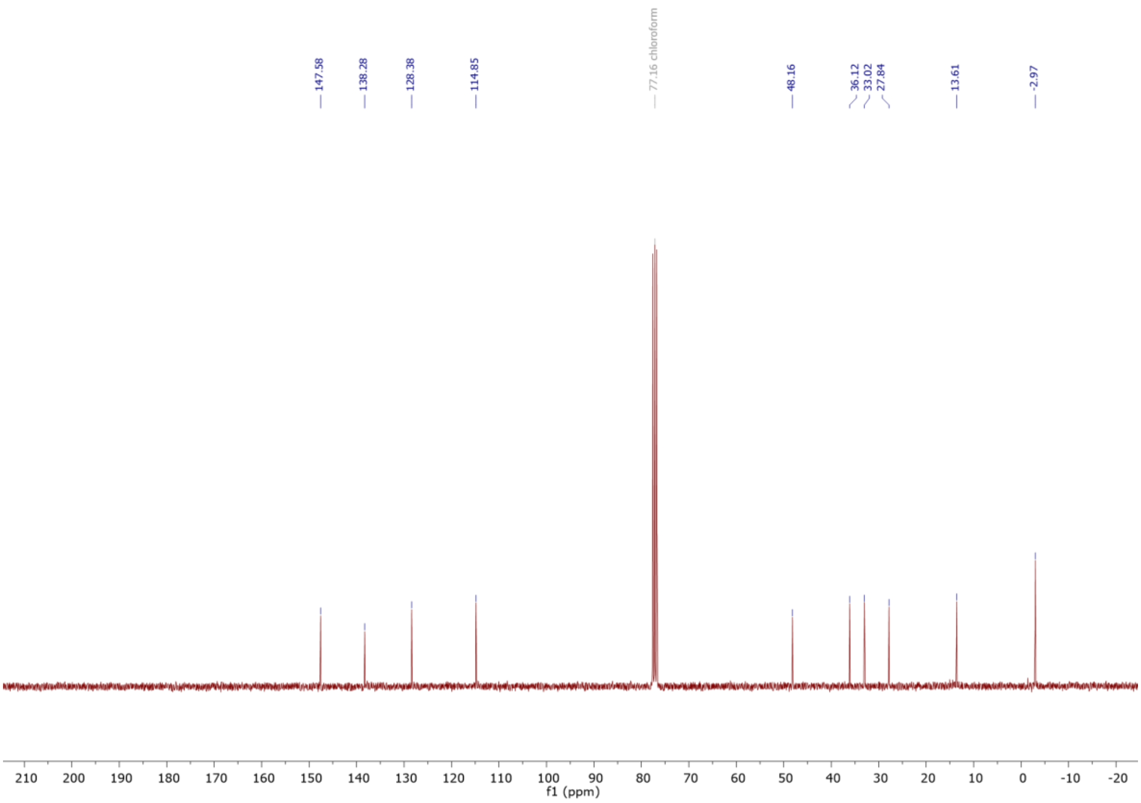

Figure S20. ^{13}C NMR spectrum of 3f

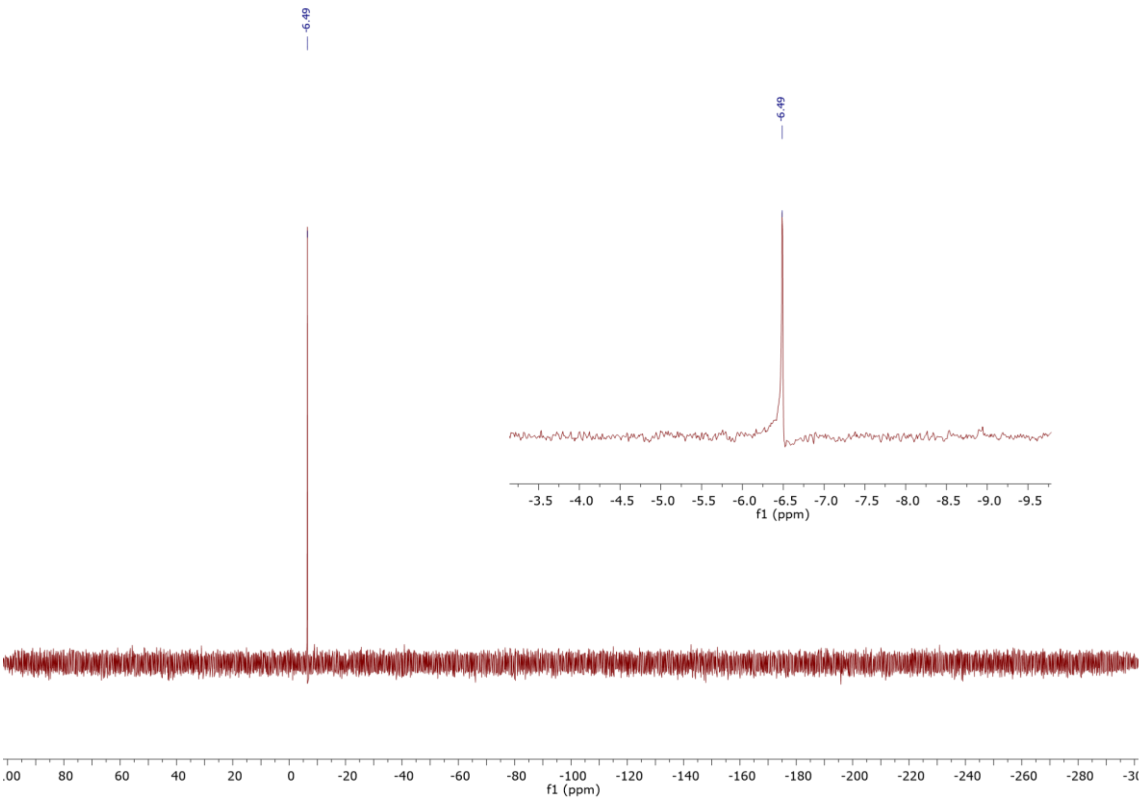

Figure S21. ^{29}Si NMR spectrum of 3f

2.8. Product 3g

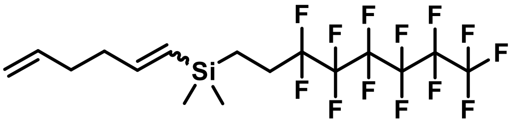

^{1}H NMR (300 MHz, $CDCl_3$) δ 6.07 RCH=CHSi (dt, *J* = 18.7, 5.9 Hz, 1H), 5.80 CH_2=CHR (ddt, *J* = 16.5, 10.2, 6.3 Hz, 1H), 5.59 RCH=CHSi (d, *J* = 18.7 Hz, 1H), 5.08 – 4.88 CH_2=CHR (m, 2H), 2.32 – 2.10 (m, 4H), 2.09 – 1.86 (m, 2H), 0.85 – 0.70 $SiCH_2$ (m, 2H), 0.09 $SiMe_2$ (s, 6H), Isomer *β-Z*: 6.38 RCH=CHSi (dt, *J* = 14.4, 7.3 Hz), 5.45 RCH=CHSi (d, *J* = 14.0 Hz).
^{13}C NMR (75 MHz, $CDCl_3$) δ 148.62, 138.11, 127.28, 114.95, 36.10, 32.94, 26.04, 5.20, -3.28.
^{29}Si NMR (79 MHz, $CDCl_3$) δ -5.54.
MS (EI, m/z): 288.9 (4.5), 244.9 (6.8), 238.9 (11.3), 140.2 (8.8), 138.9 (67.4), 114.9 (9.4), 110.9 (18.0), 88.9 (18.4), 84.9 (9.0), 80.9 (26.3), 79.0 (79.7), 77.9 (7.4), 76.9 (81.9), 73.0 (36.4), 68.9 (10.2), 62.9 (16.6), 59.0 (100)

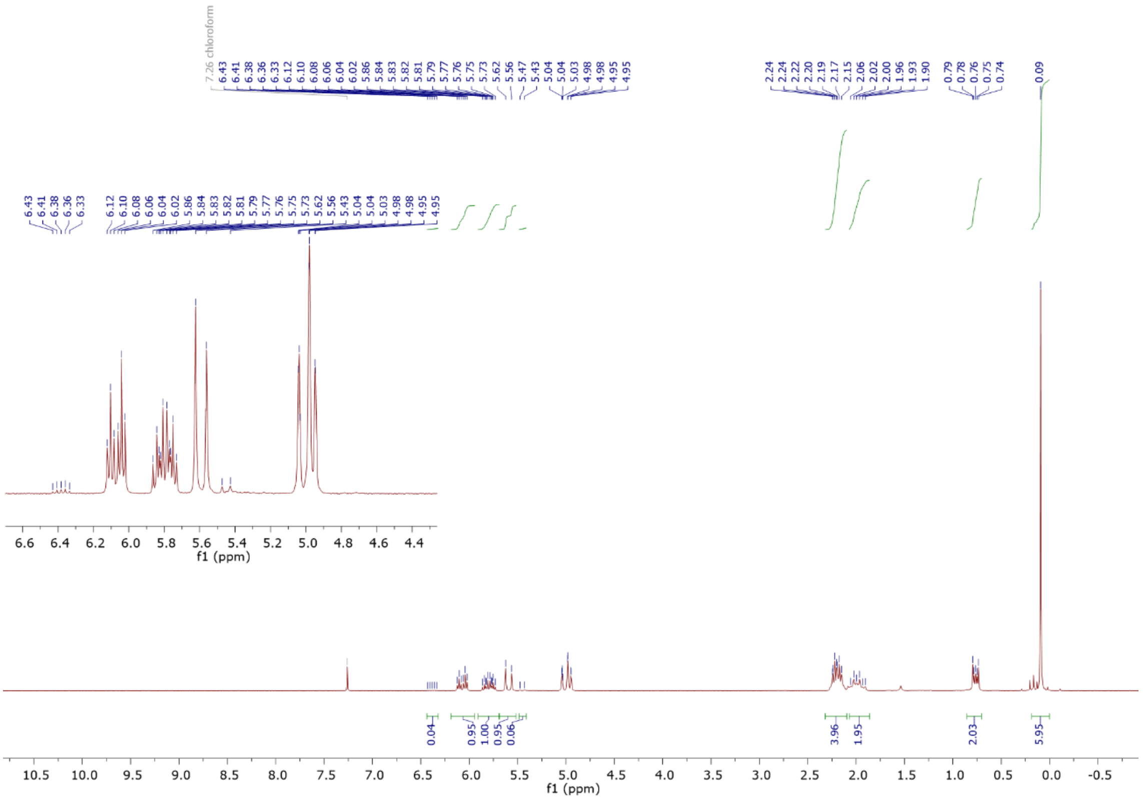

Figure S22. ^{1}H NMR spectrum of 3g

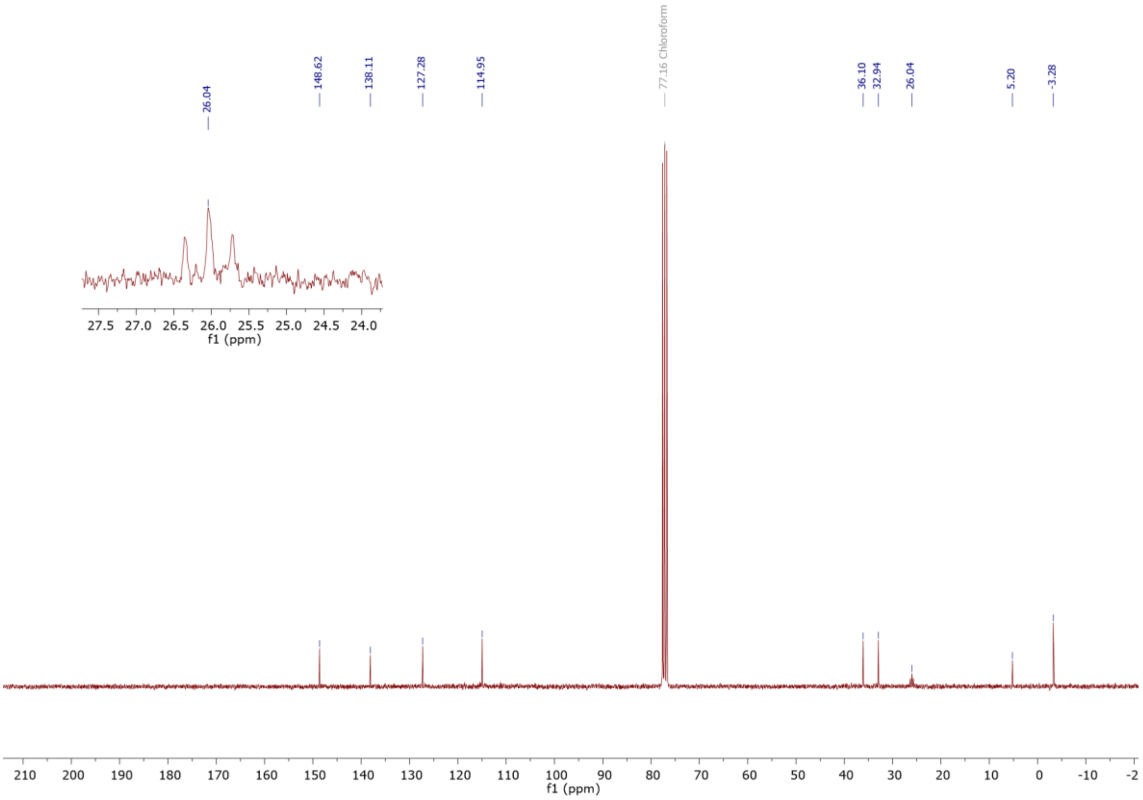

Figure S23. ^{13}C NMR spectrum of 3g

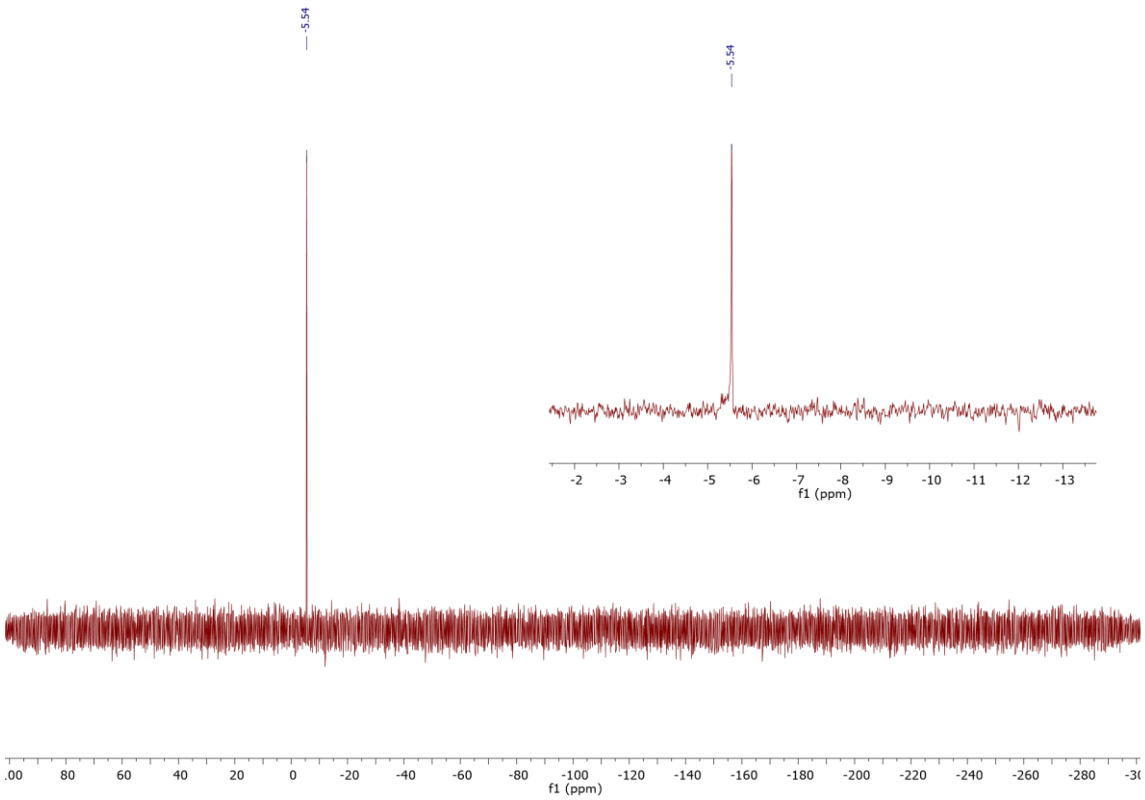

Figure S24. ^{29}Si NMR spectrum of 3g

2.9. Product 3h

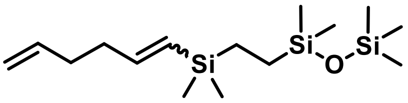

^{1}H NMR (300 MHz, $CDCl_3$) δ 6.10 R$\underline{\text{CH}}$=CHSi (dt, J = 18.7, 5.8 Hz, 1H), 5.82 CH_2=$\underline{\text{CH}}$R (ddt, J = 16.6, 10.3, 6.3 Hz, 1H), 5.63 RCH=$\underline{\text{CH}}$Si (d, J = 18.7 Hz, 1H), $\underline{CH_2}$=CHR 5.09 – 4.84 (m, 2H), 2.18 (m, 4H), 0.39 $SiCH_2$ (s, 4H), 0.12 – (-0.07) $SiCH_3$ (m, 21H), Isomer β-Z: 6.30 R$\underline{\text{CH}}$=CHSi (dt, J = 14.3, 7.2 Hz), 5.46 RCH=$\underline{\text{CH}}$Si (d, J = 14.2 Hz).
^{13}C NMR (75 MHz, $CDCl_3$) δ 147.04, 138.36, 130.18, 114.80, 35.87, 32.94, 10.45, 8.19, 0.96, -0.18, -2.06.
^{29}Si NMR (79 MHz, $CDCl_3$) δ 8.94, 3.07, -4.41.
MS (EI, m/z): 299.1 $[M-15]^+$ (4.7), 233.1 (5.5), 232.0 (4.9), 213.0 (18.8), 144.9 (9.1), 139.0 (8.7), 134.1 (13.8), 132.9 (100), 130.9 (5.1), 72.9 (27.0), 58.9 (12.0)

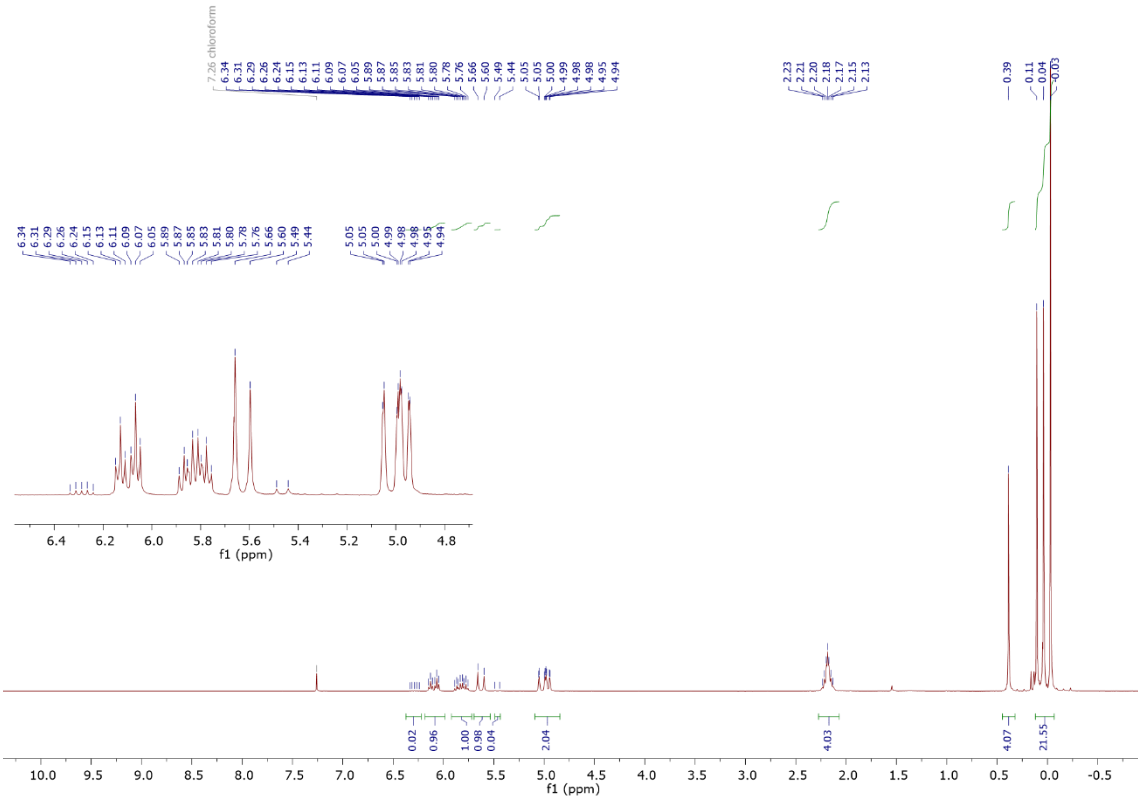

Figure S25. ^{1}H NMR spectrum of 3h

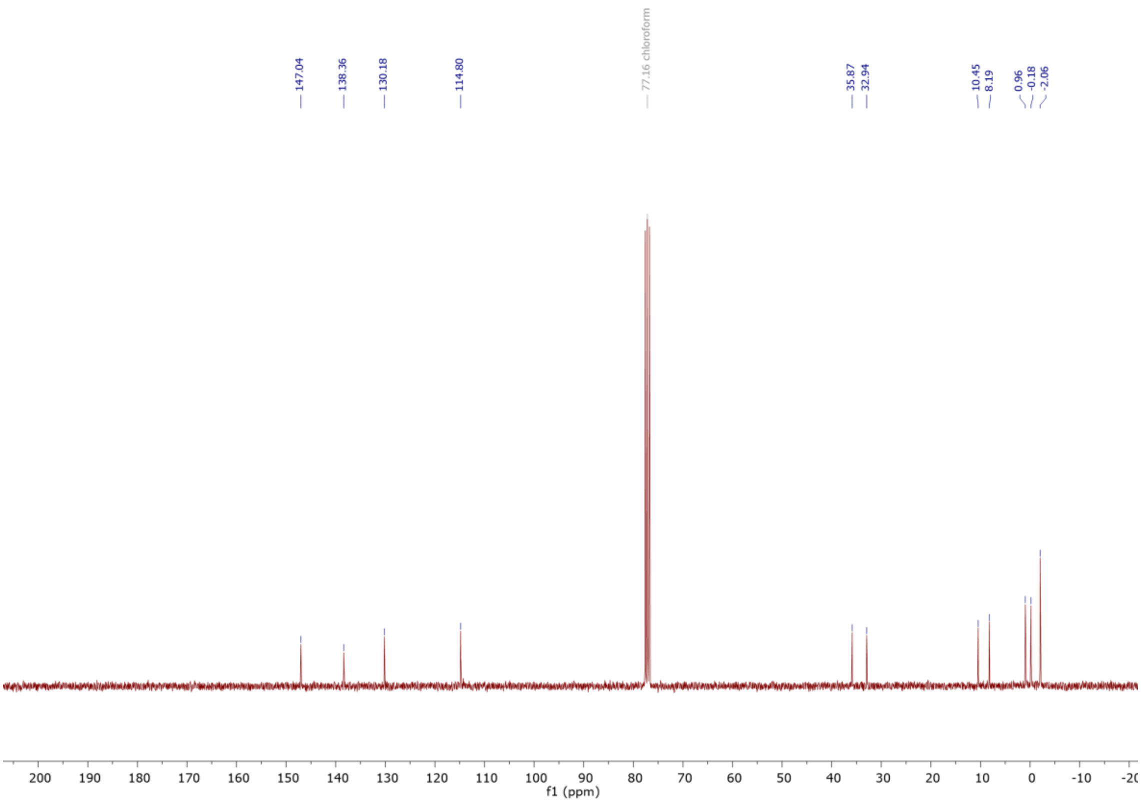

Figure S26. ^{13}C NMR spectrum of 3h

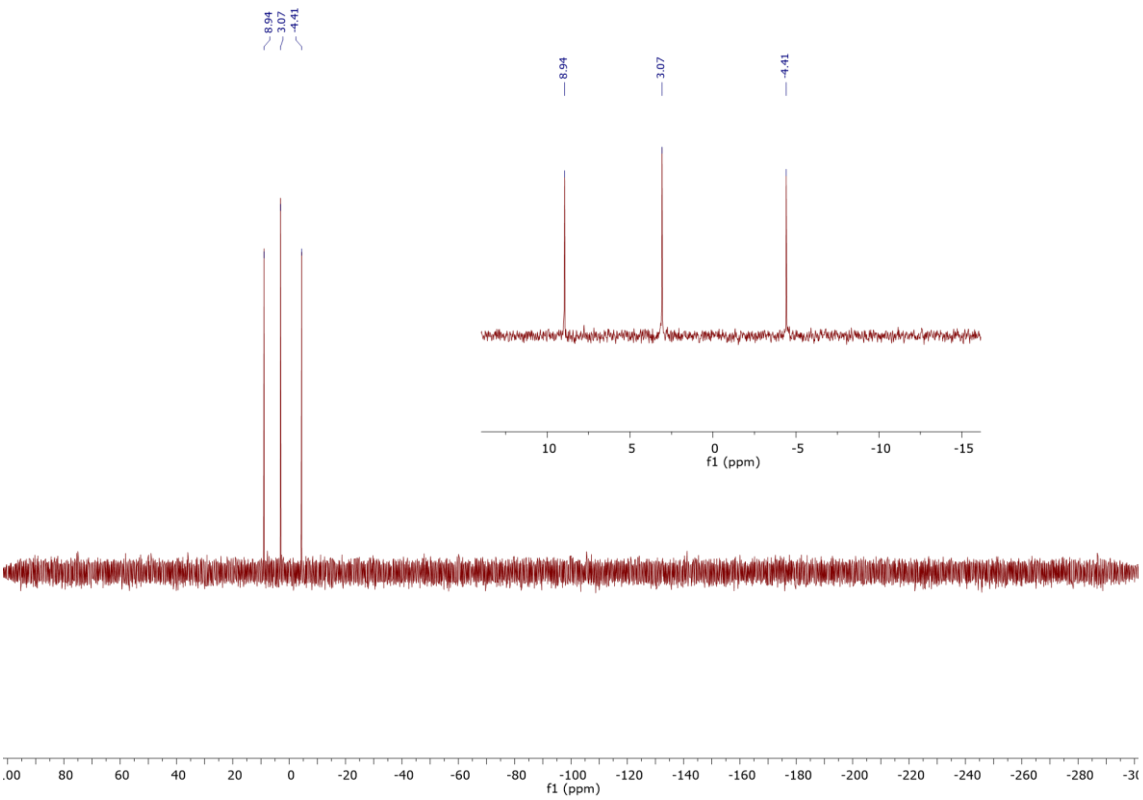

Figure S27. ^{29}Si NMR spectrum of 3h

2.10. Product 3i

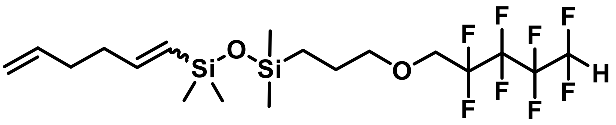

^{1}H NMR (300 MHz, $CDCl_3$) δ 6.34 – 5.71 CF_2H, R$\underline{\text{CH}}$=CHSi, CH_2=$\underline{\text{CH}}$R (m, 3H), 5.62 RCH=$\underline{\text{CH}}$Si (d, *J* = 18.7 Hz, 1H), 5.46 RCH=$\underline{\text{CH}}$Si (*β*-*Z* isomer) (d, *J* = 14.2 Hz), 5.08 – 4.90 $\underline{CH_2}$=CHR (m, 2H), 3.91 CH_2O (t, *J* = 14.0 Hz, 2H), 3.55 CH_2O (t, *J* = 6.9 Hz, 2H), 2.18 (m, 4H), 1.68 – 1.58 (m, 2H), 0.56 – 0.45 $SiCH_2$ (m, 2H), 0.10 $SiMe_2$ (s, 6H), 0.06 $SiMe_2$ (s, 6H).
^{13}C NMR (101 MHz, $CDCl_3$) δ 147.27, 138.32, 129.96, 114.80, 75.98, 67.64, 35.83, 32.90, 23.46, 14.10, 0.85, 0.42.
^{29}Si NMR (79 MHz, $CDCl_3$) δ 8.00, -3.82.
MS (EI, m/z): 471.1 (3.7) $[M-15]^+$, 331. (3.5), 213.0 (7.4), 160.9 (6.6), 156.9 (6.5), 154.8 (6.6), 153.0 (9.1), 152.1 (13.5), 150.9 (100), 138.1 (11.4), 136.9 (91.4), 135.1 (9.9), 134.2 (13.5), 132.9 (97.9), 78.9 (7.0), 73.0 (11.9), 58.9 (5.6).

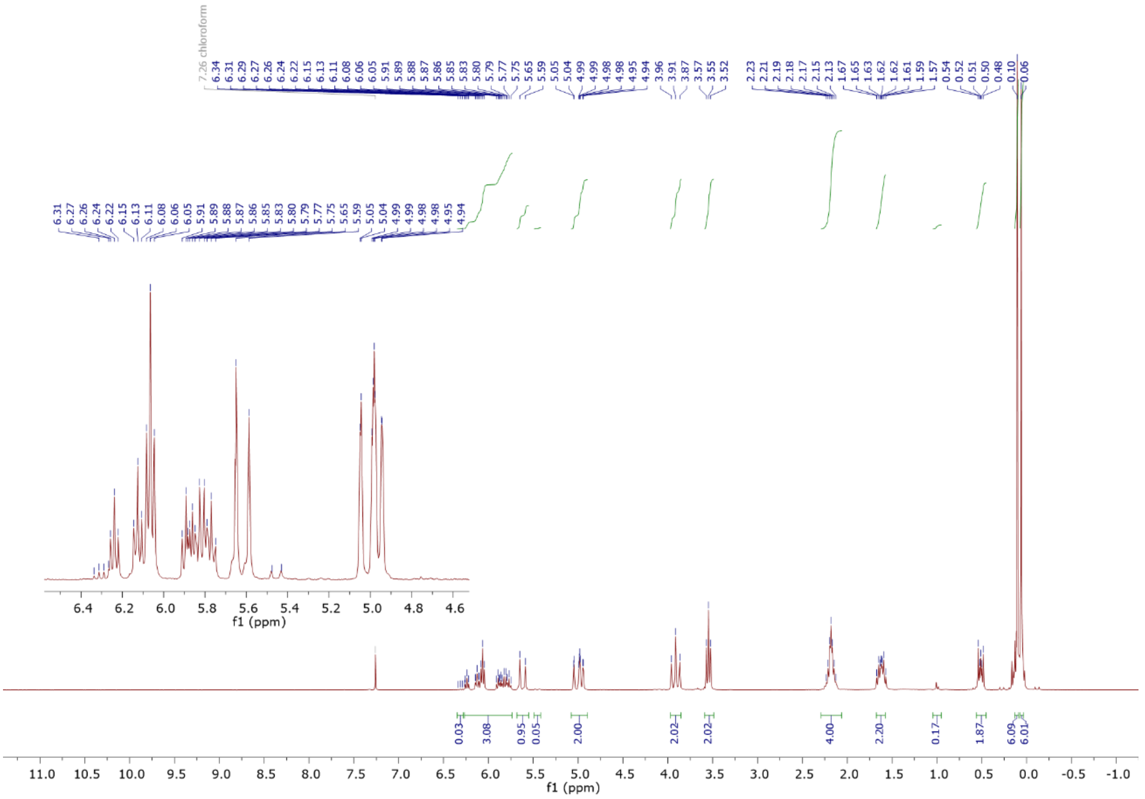

Figure S28. ^{1}H NMR spectrum of 3i

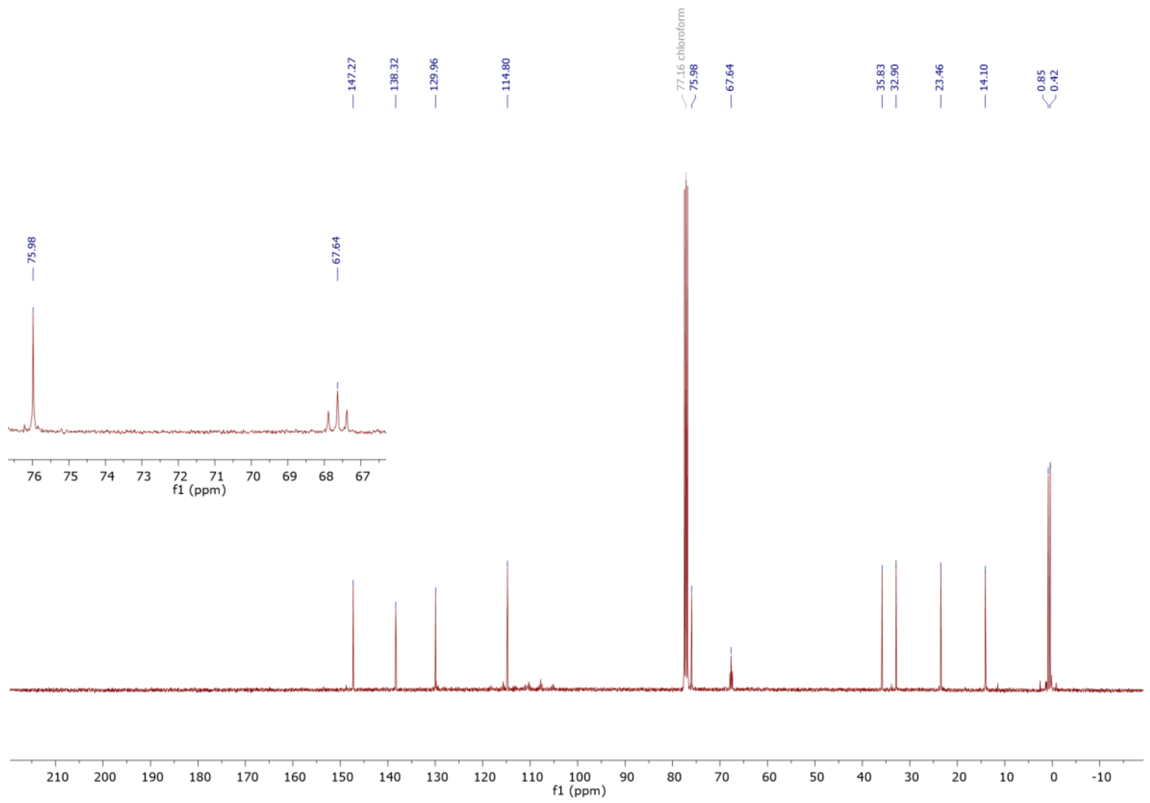

Figure S29. ^{13}C NMR spectrum of 3i

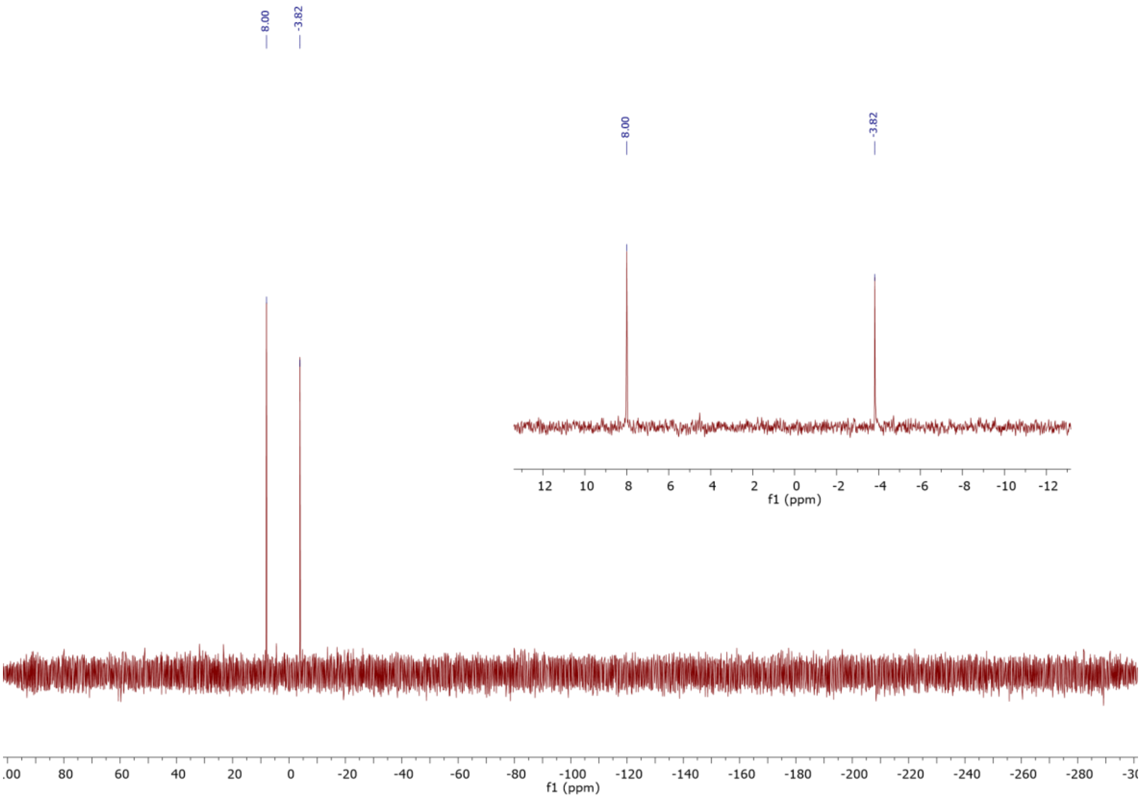

Figure S30. ^{29}Si NMR spectrum of 3i

2.11. Product 3j

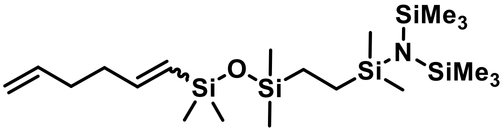

^{1}H NMR (300 MHz, $CDCl_3$) δ 6.10 RCH=CHSi (dt, *J* = 18.6, 5.8 Hz, 1H), 5.82 CH_2=CHR (ddt, *J* = 16.5, 10.2, 6.3 Hz, 1H), 5.63 RCH=CHSi (d, *J* = 18.7 Hz, 1H), 5.11 – 4.86 (m, 2H), 2.18 (m, 4H), 0.55 – 0.47 $SiCH_2$ (m, 2H), 0.43 – 0.35 $SiCH_2$ (m, 2H), 0.17 $SiMe_3$, $SiMe_2$ (24H), 0.11 $SiMe_2$ (s, 6H), 0.05 $SiMe_2$ (s, 6H), Isomer *β-Z*: 6.29 RCH=CHSi (dt, *J* = 14.4, 7.3 Hz), 5.47 RCH=CHSi (d, *J* = 14.2 Hz).
^{13}C NMR (75 MHz, $CDCl_3$) δ 147.04, 138.37, 130.16, 114.79, 35.87, 32.95, 12.11, 10.76, 5.74, 3.19, 0.99, -0.13.
^{29}Si NMR (79 MHz, $CDCl_3$) δ 8.89, 5.08, 2.23, -4.34.
MS (EI, m/z): 459.2 (0.2) M^+, 220.1 (12.0), 219.2 (22.7), 218.0 (100), 216.0 (11.2), 202.0 (6.7), 132.9 (18.4), 129.9 (7.7), 72.9 (6.5).

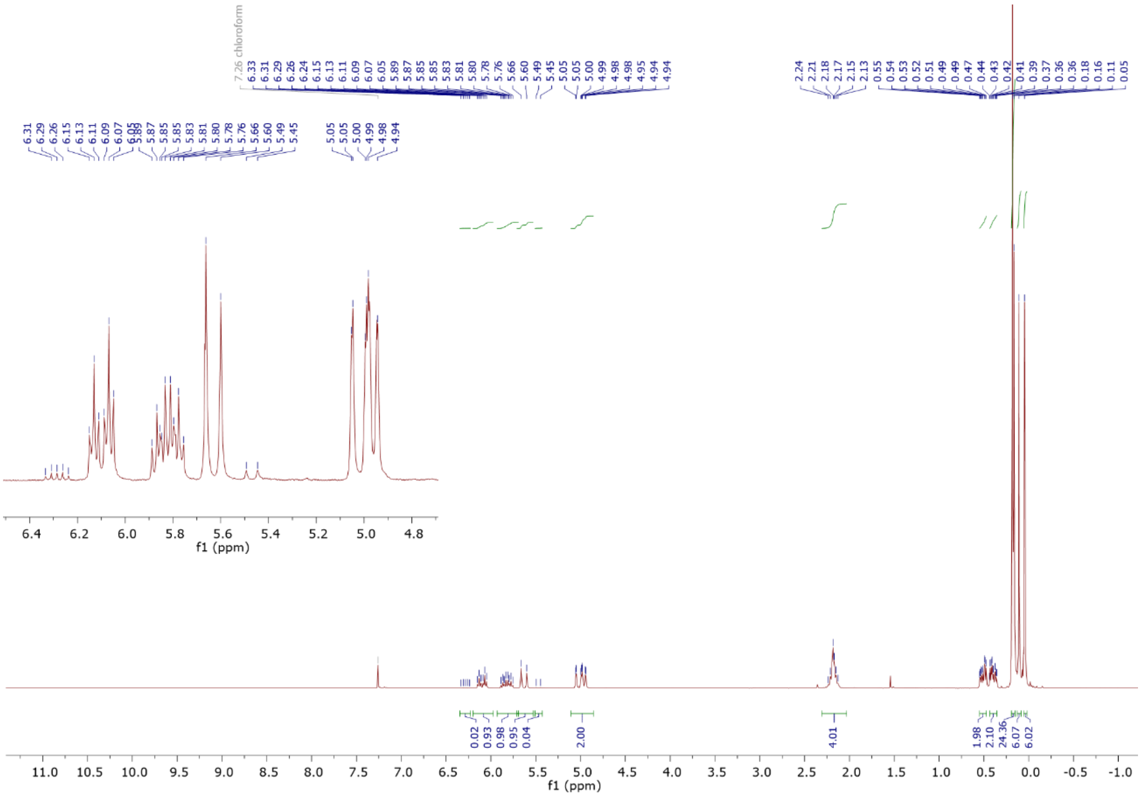

Figure S31. ^{1}H NMR spectrum of 3j

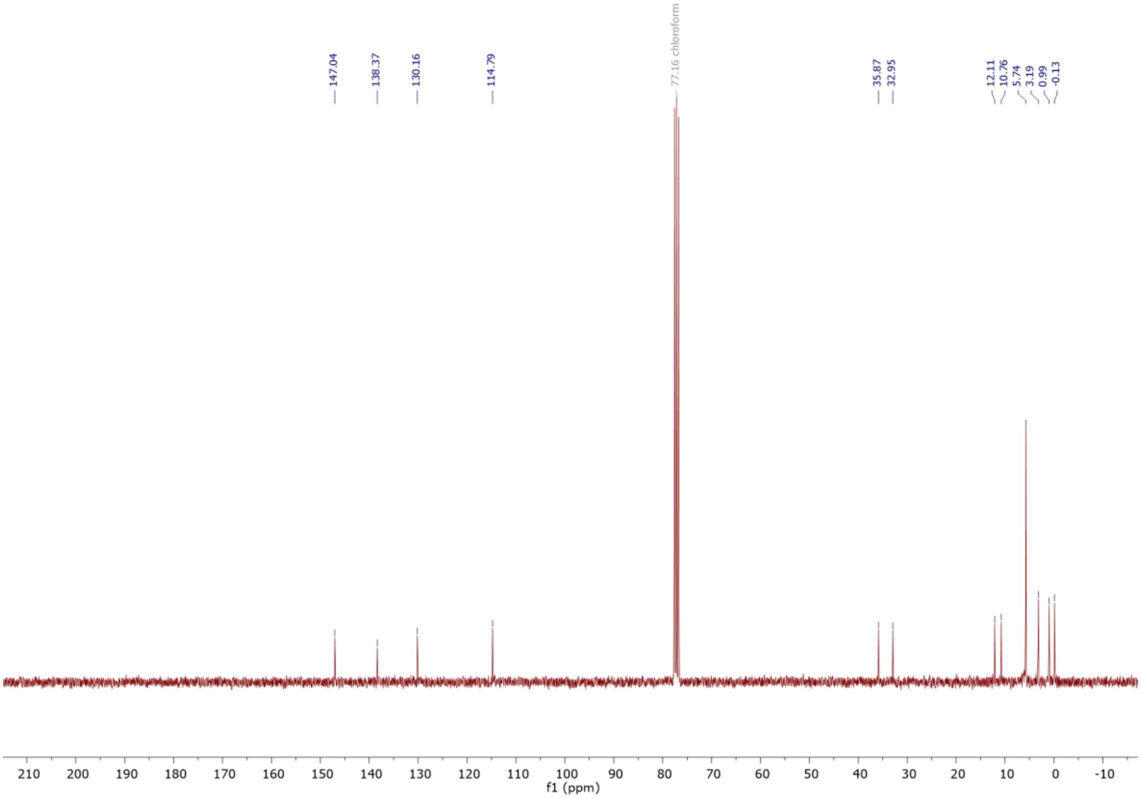

Figure S32. ^{13}C NMR spectrum of 3j

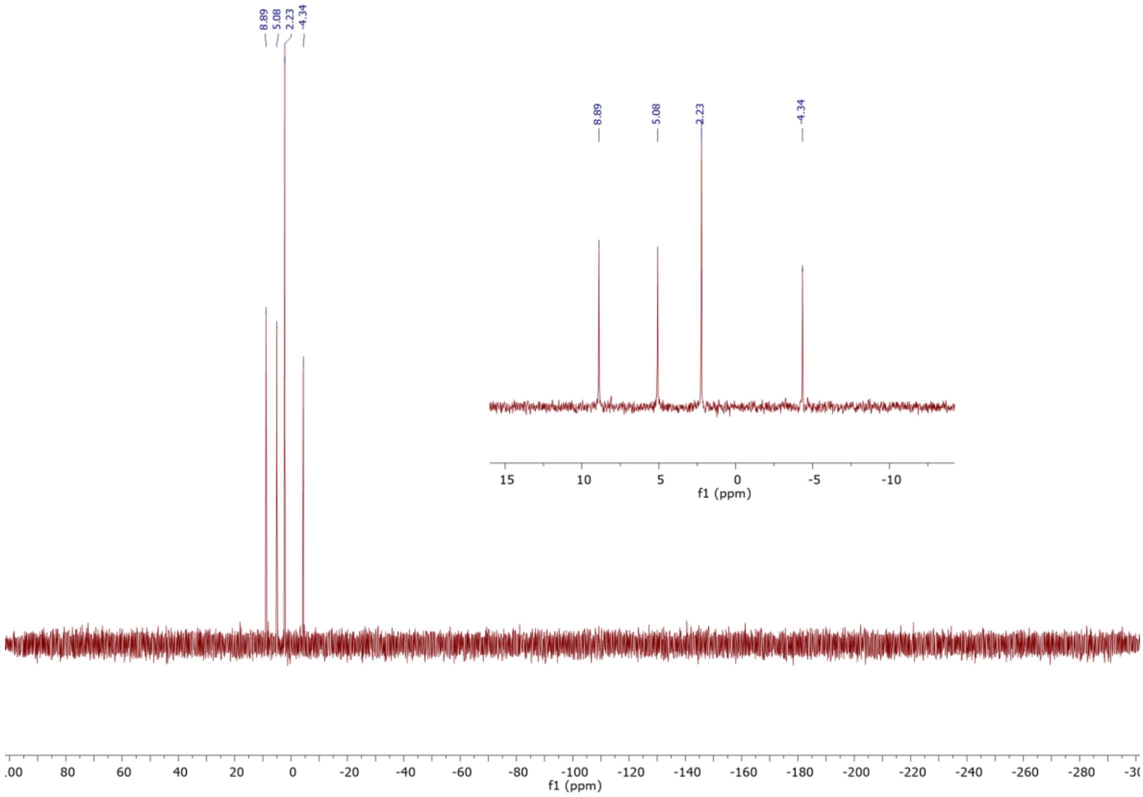

Figure S33. ^{29}Si NMR spectrum of 3j

2.12. Product 3k

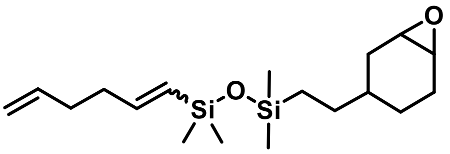

^{1}H NMR (300 MHz, $CDCl_3$) δ 6.08 RCH=CHSi (dt, J = 18.6, 5.8 Hz, 1H), 5.82 CH_2=CHR (ddt, J = 16.6, 10.3, 6.3 Hz, 1H), RCH=CHSi 5.61 (d, J = 18.7 Hz, 1H), 5.10 – 4.87 CH_2=CHR (m, 2H), 3.14 CH(O) (m, 2H), 2.26 – 1.94 (m, 6H), 1.87 – 1.66 (m, 1H), 1.56 – 1.26 (m, 3H), 1.22 – 1.04 (m, 3H), 0.93 – 0.78 (m, 1H), 0.46 $SiCH_2$ (m, 2H), 0.09 $SiMe_2$ (s, 6H), 0.03 $SiMe_2$ (s, 6H), Isomer *β-Z*: 6.28 RCH=CHSi (dt, J = 14.5, 7.3 Hz), 5.45 RCH=CHSi (d, J = 14.0 Hz).
^{13}C NMR (75 MHz, $CDCl_3$) δ 147.10, 138.31, 130.07, 114.82, 53.44, 52.92, 52.17, 52.13, 35.84, 35.53, 32.91, 32.42, 31.70, 30.56, 30.31, 29.75, 26.90, 25.54, 24.21, 23.74, 15.39, 15.26, 0.93, 0.40.
^{29}Si NMR (79 MHz, $CDCl_3$) δ 8.30, -4.23.
MS (EI, m/z): 150.1 (7.1), 148.9 (52.4), 135.2 (7.6), 134.5 (6.6), 132.9 (100), 118.9 (9.7), 116.9 (5.2), 108.9 (7.3), 92.9 (8.3), 81.0 (14.7), 80.0 (6.6), 79.0 (11.9), 72.9 (9.0), 67.0 (24.6).

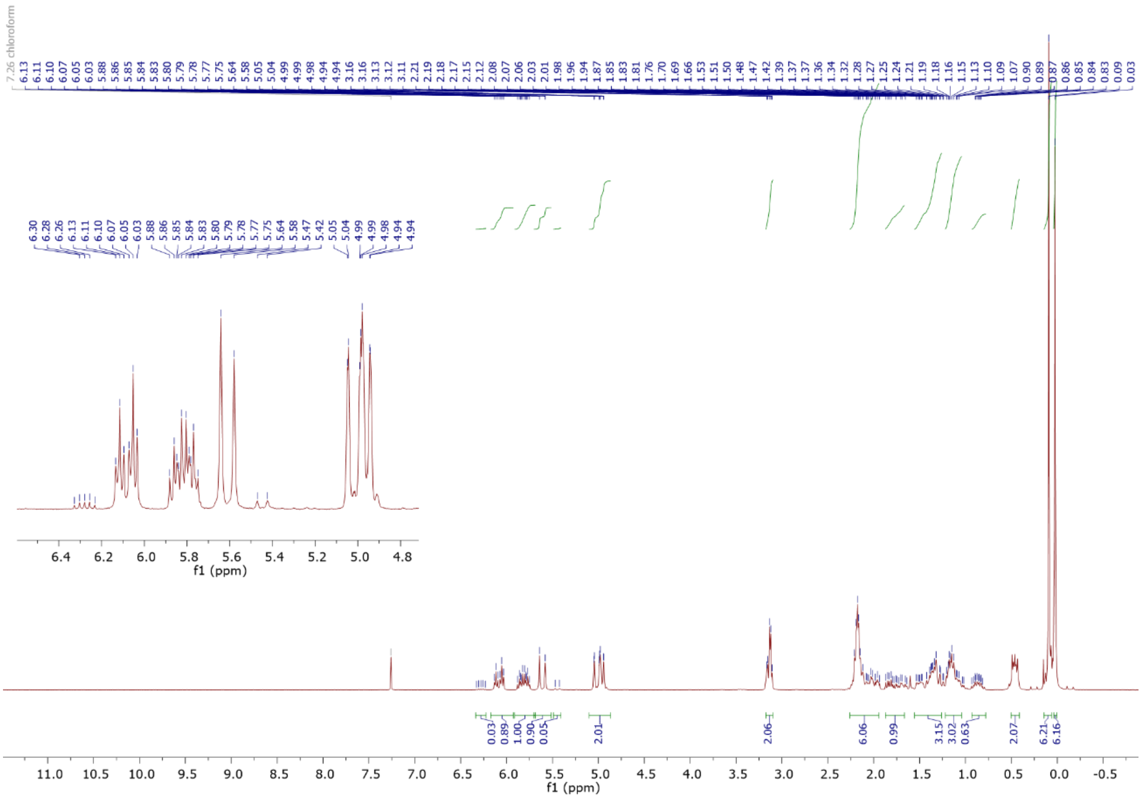

Figure S34. ^{1}H NMR spectrum of 3k

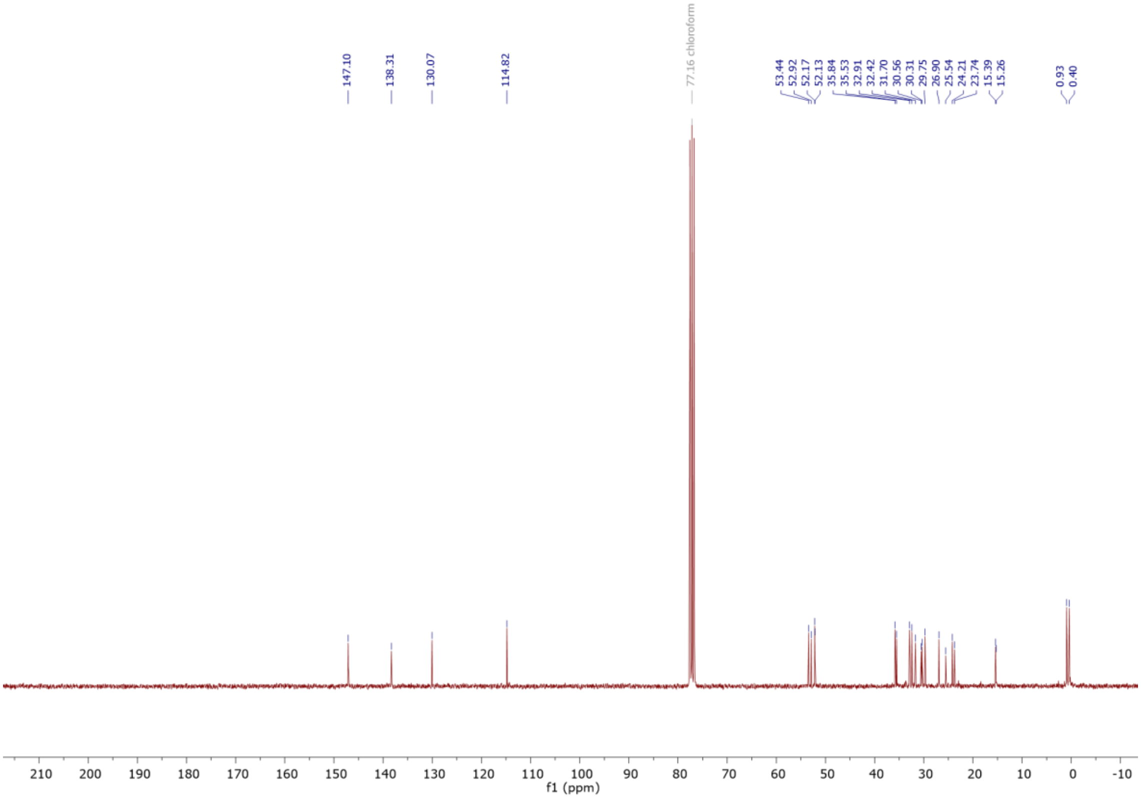

Figure S35. ^{1}H NMR spectrum of 3k

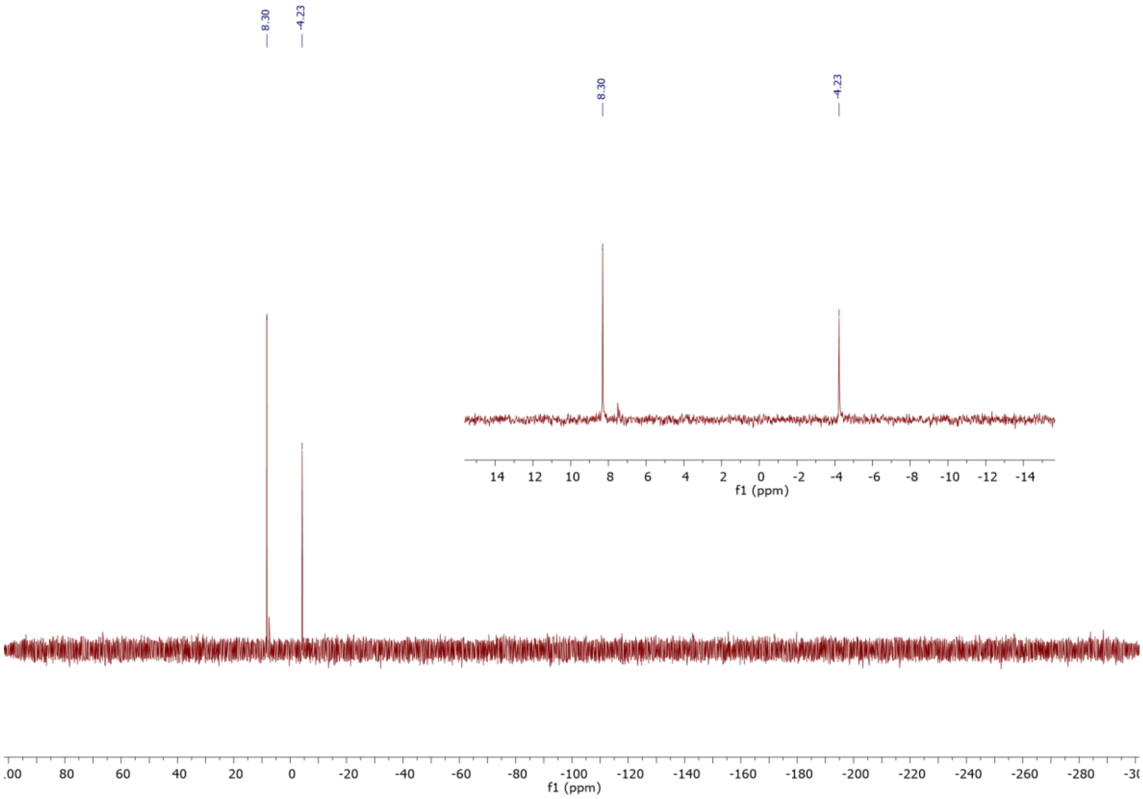

Figure S36. ^{29}Si NMR spectrum of 3k

2.13. Product 3l

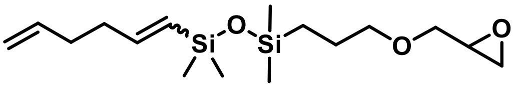

^{1}**H NMR** (300 MHz, $CDCl_3$) δ 6.09 RCH=CHSi (dt, *J* = 18.7, 5.9 Hz, 1H), 5.81 CH_2=CHR (ddt, *J* = 16.6, 10.3, 6.3 Hz, 1H), 5.61 RCH=CHSi (d, *J* = 18.7 Hz, 1H), 5.08 – 4.85 CH_2=CHR (m, 2H), 3.70 (dd, *J* = 11.5, 3.1 Hz, 1H), 3.52 – 3.30 (m, 3H), 3.15 (m, 1H), 2.82 – 2.76 (m, 1H), 2.61 (dd, *J* = 5.0, 2.7 Hz, 1H), 2.26 – 2.09 (m, 4H), 1.66 – 1.55 (m, 2H), 0.56 – 0.45 $SiCH_2$ (m, 2H), 0.10 $SiMe_2$ (s, 6H), 0.05 $SiMe_2$ (s, 6H), Isomer *β-Z*: 6.28 RCH=CHSi (dt, *J* = 14.4, 7.2 Hz), 5.45 RCH=CHSi (d, *J* = 14.1 Hz).

^{13}**C NMR** (101 MHz, $CDCl_3$) δ 147.16, 138.32, 130.03, 114.80, 74.52, 71.57, 51.02, 44.52, 35.83, 32.90, 23.62, 14.37, 0.90, 0.45.

^{29}**Si NMR** (79 MHz, $CDCl_3$) δ 8.16, -4.01.

MS (EI, m/z): 313.1 $[M-15]^+$, 174.9 (29.7), 148.9 (8.3), 135.0 (14.4), 134.1 (13.8), 132.9 (100), 130.9 (5.5), 118.9 (7.4), 92.9 (5.3), 78.9 (7.0), 67.0 (14.5).

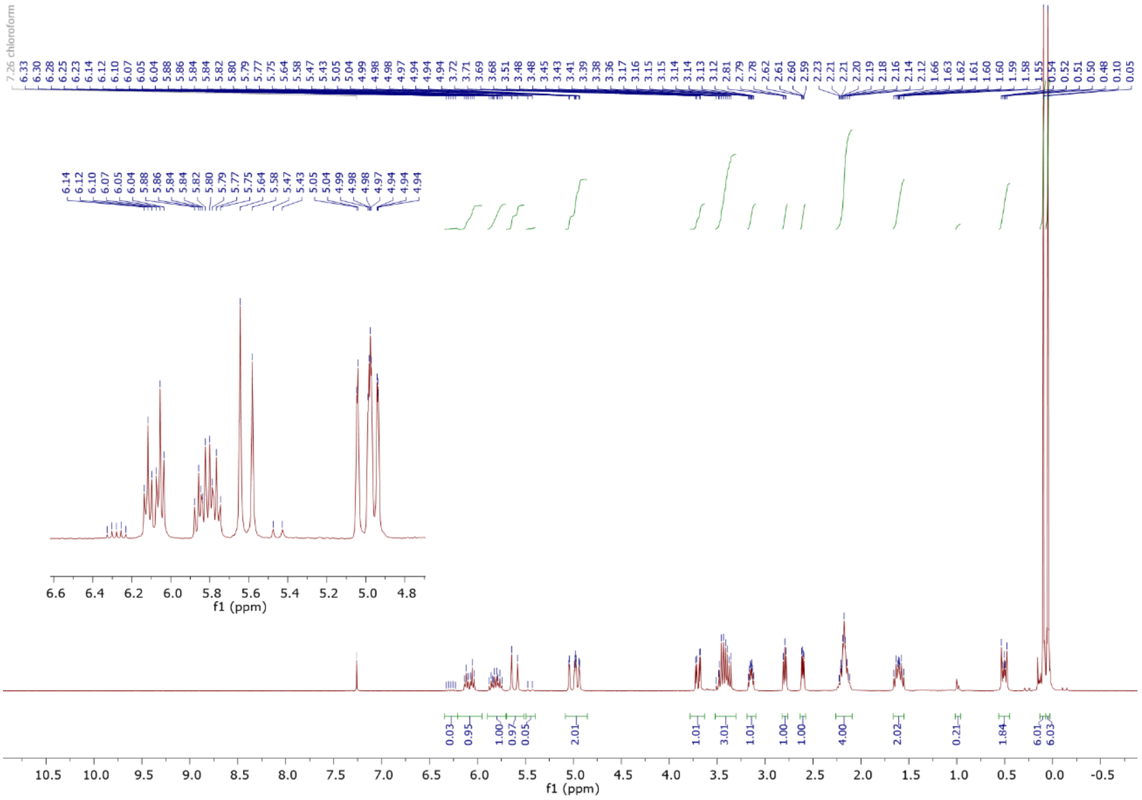

Figure S37. ^{1}H NMR spectrum of 3l

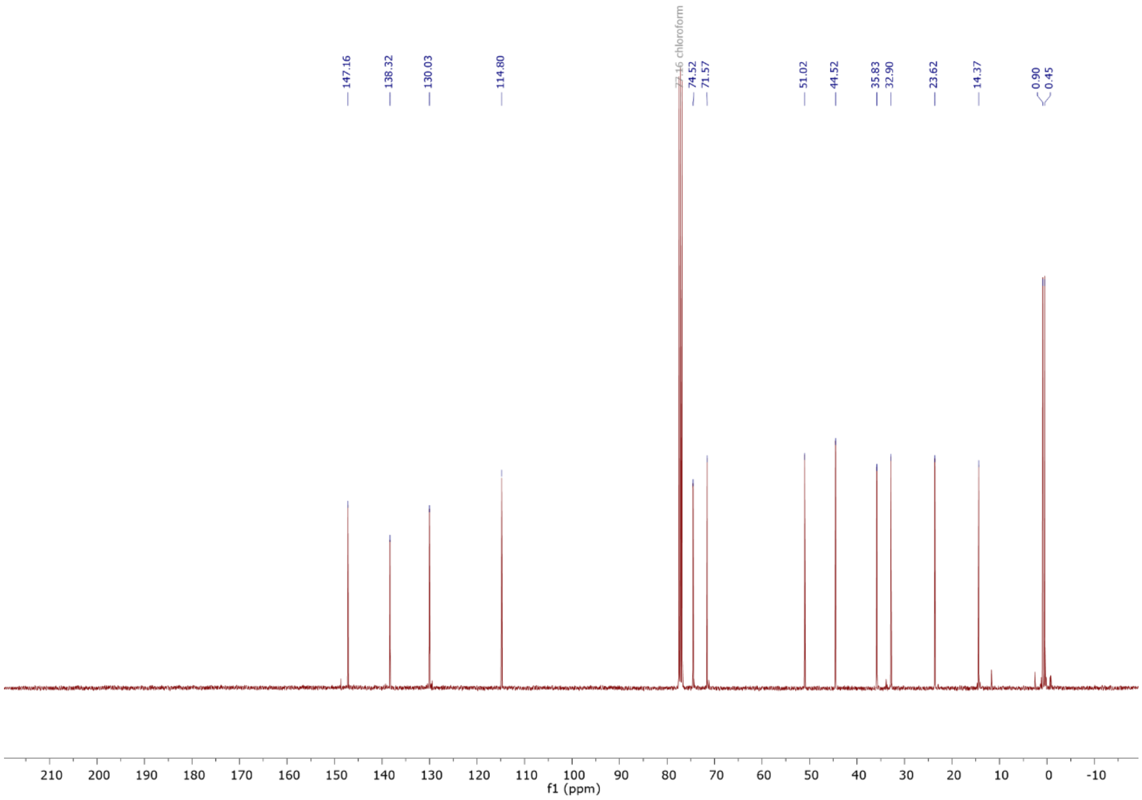

Figure S38. ^{13}C NMR spectrum of 3l

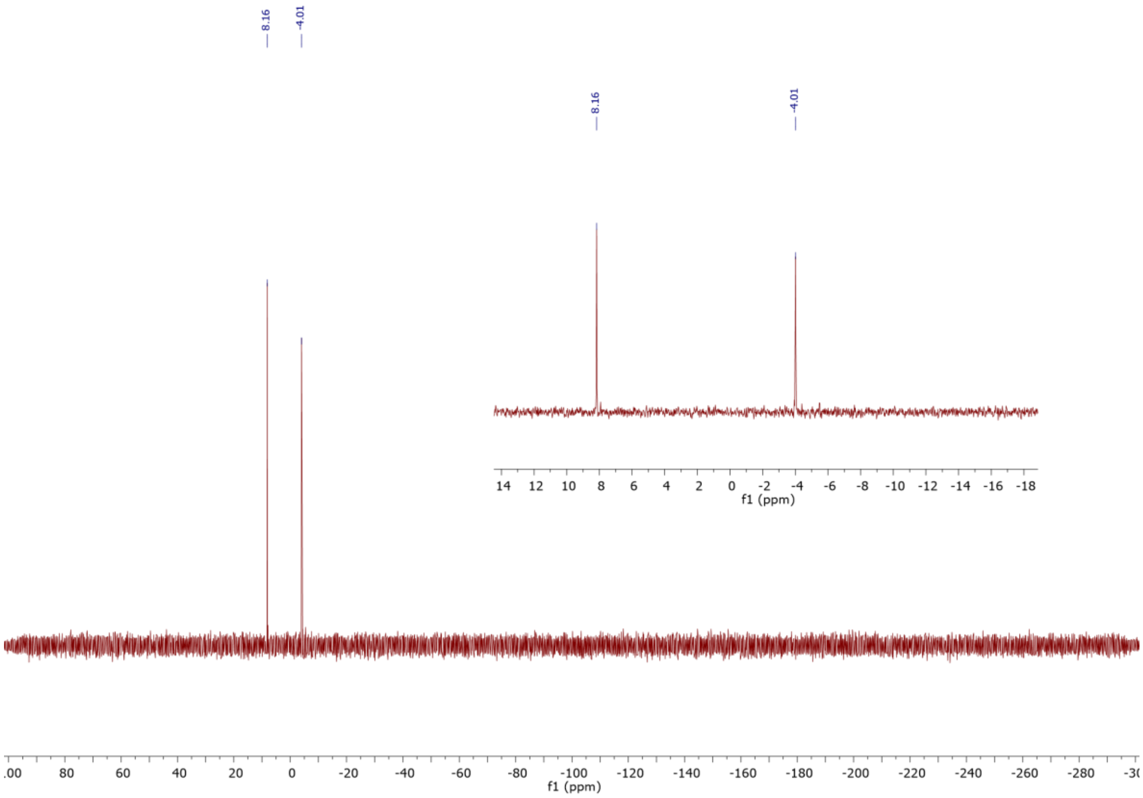

Figure S39. ^{29}Si NMR spectrum of 3l

2.14. Product 3m

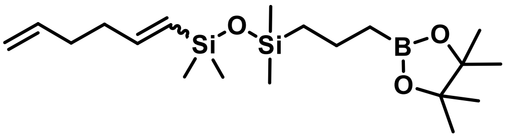

^{1}H NMR (300 MHz, $CDCl_3$) δ 6.08 RCH=CHSi (dt, J = 18.7, 5.9 Hz, 1H), 5.81 CH_2=CHR (ddt, J = 16.6, 10.2, 6.3 Hz, 1H), 5.62 RCH=CHSi (d, J = 18.7 Hz, 1H), 5.07 – 4.88 CH_2=CHR (m, 2H), 2.34 – 2.00 (m, 4H), 1.50 – 1.38 (m, 2H), 1.24 $C(CH_3)_2$ (s, 12H), 0.82 BCH_2 (t, J = 7.6 Hz, 2H), 0.53 $SiCH_2$ (m, 2H), 0.09 $SiMe_2$ (s, 6H), 0.03 $SiMe_2$ (s, 6H), Isomer *β-Z*: 6.27 RCH=CHSi (dt, J = 14.3, 7.2 Hz), 5.45 RCH=CHSi (d, J = 14.3 Hz).
^{13}C NMR (75 MHz, $CDCl_3$) δ 147.01, 138.40, 130.18, 114.78, 82.94, 35.85, 32.93, 24.98, 21.84, 18.08, 0.95, 0.58.
^{29}Si NMR (79 MHz, $CDCl_3$) δ 7.80, -4.42.
MS (EI, m/z): 367.1 $[M-15]^+$, 267.0 (8.5), 227.1 (5.8), 225.0 (5.9), 212.9 (5.3), 203.1 (7.1), 202.2 (8.3), 201.0 (43.9), 199.9 (12.2), 187.0 (11.8), 175.1 (16.1), 174.4 (8.2), 172.9 (15.4), 151.0 (6.8), 158.9 (24.8), 156.9 (7.9), 144.9 (20.5), 135.1 (7.9), 134.1 (12.7), 132.9 (100), 130.9 (9.6), 116.9 (8.8), 84.0 (36.2), 83.0 (35.6), 73.0 (9.7),69.0 (5.6)

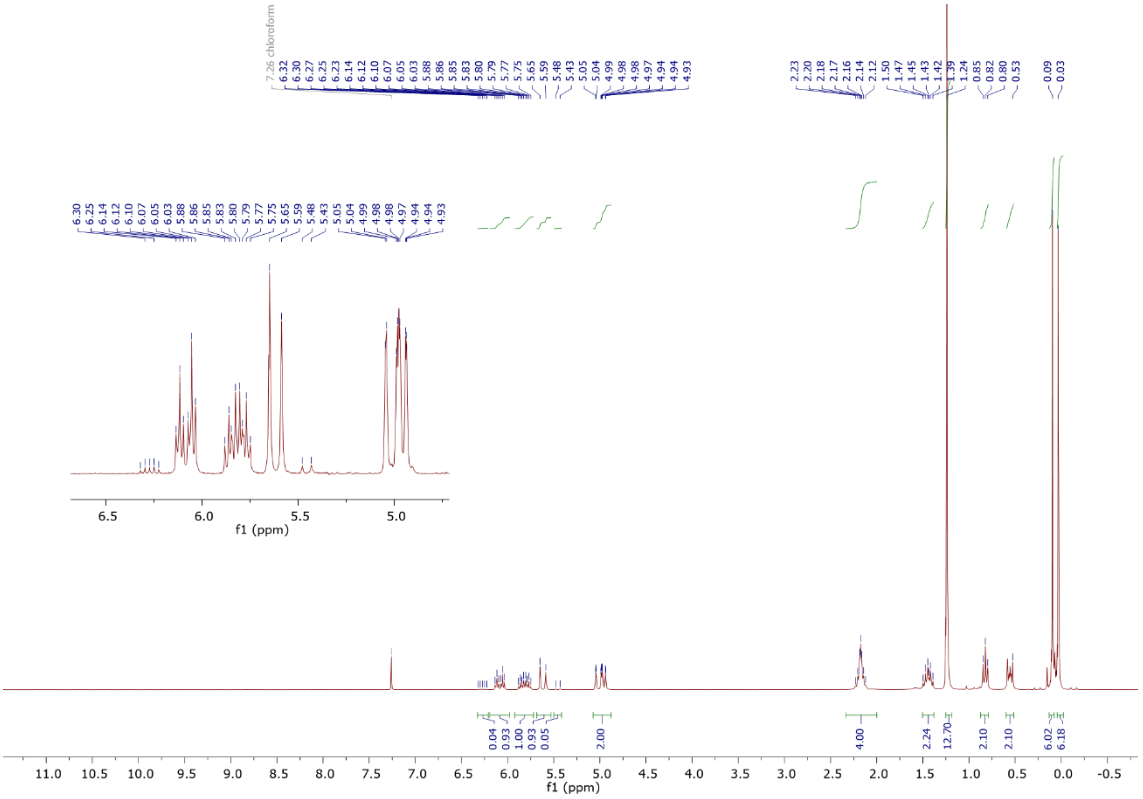

Figure S40. ^{1}H NMR spectrum of 3m

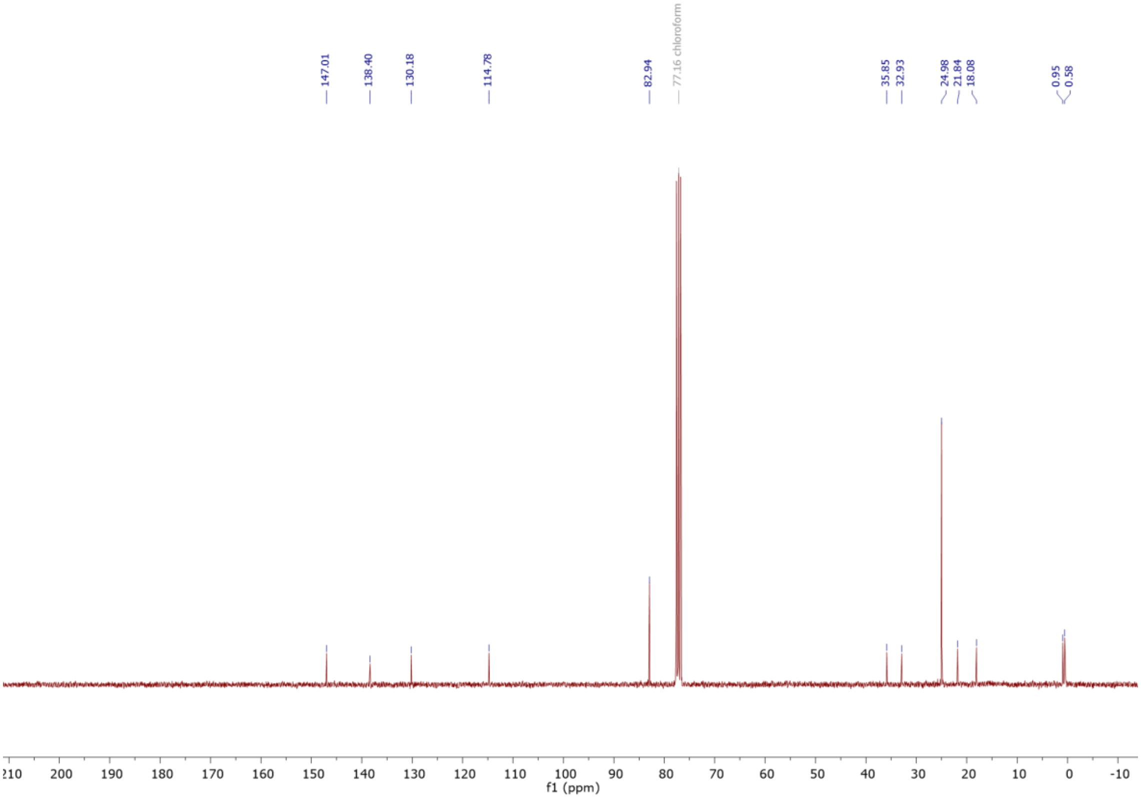

Figure S41. ^{13}C NMR spectrum of 3m

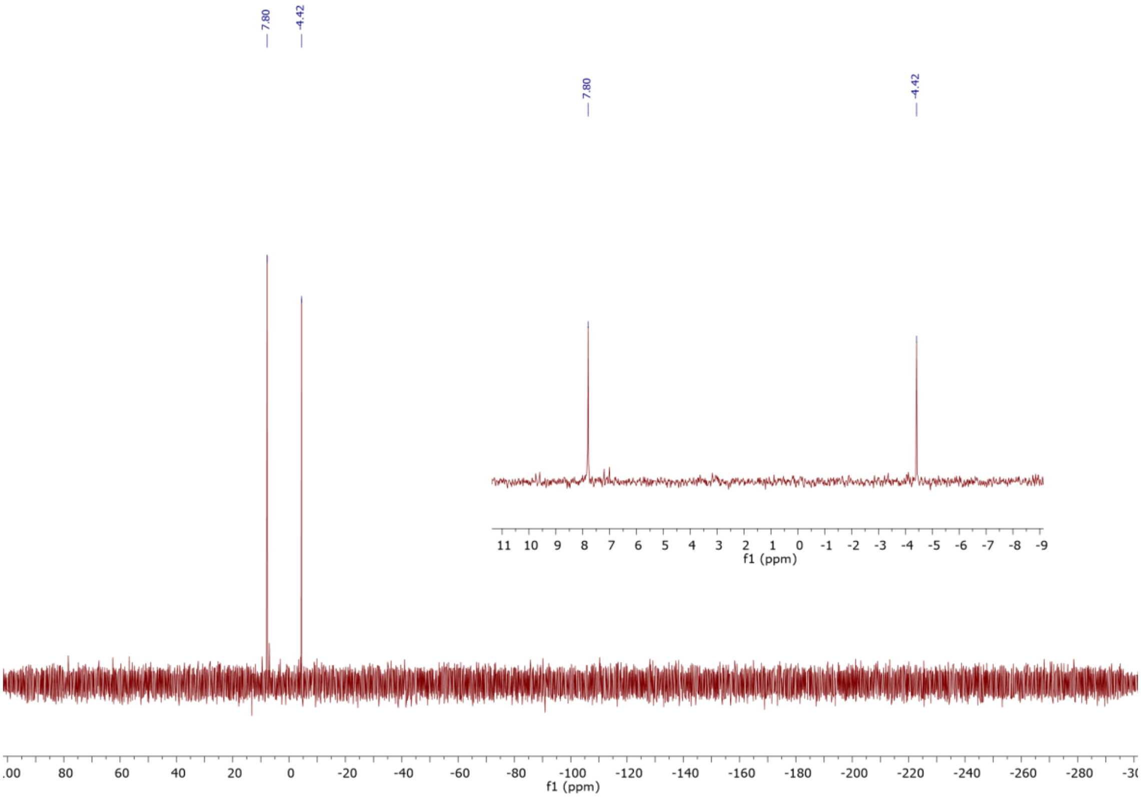

Figure S42. ^{29}Si NMR spectrum of 3m

2.15. Product 4a

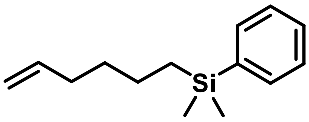

^{1}H NMR (300 MHz, $CDCl_3$) δ 7.56 – 7.50 (m, 2H), 7.42 – 7.34 (m, 3H), 5.81 CH_2=<u>CH</u>R (ddt, *J* = 16.9, 10.1, 6.7 Hz, 1H), 5.12 – 4.76 <u>CH_2</u>=CHR (m, 2H), 2.06 (q, *J* = 6.8 Hz, 2H), 1.48 – 1.28 (m, 4H), 0.84 – 0.72 $SiCH_2$ (m, 2H), 0.28 $SiMe_2$ (s, 6H).
^{13}C NMR (75 MHz, $CDCl_3$) δ 139.78, 139.21, 133.69, 128.90, 127.84, 114.30, 33.60, 32.92, 23.51, 15.69, -2.88.
^{29}Si NMR (79 MHz, $CDCl_3$) δ -3.08.
MS (EI, m/z): 218.0 M^+, 203.0 (1.4) $[M-15]^+$, 139.9 (22.4), 136.1 (13.6), 134.9 (100), 124.9 (8.7), 120.9 (19.9), 111.9 (6.3), 106.9 (5.4), 104.8 (6.1).
Product contains 4% of disubstituted derivative

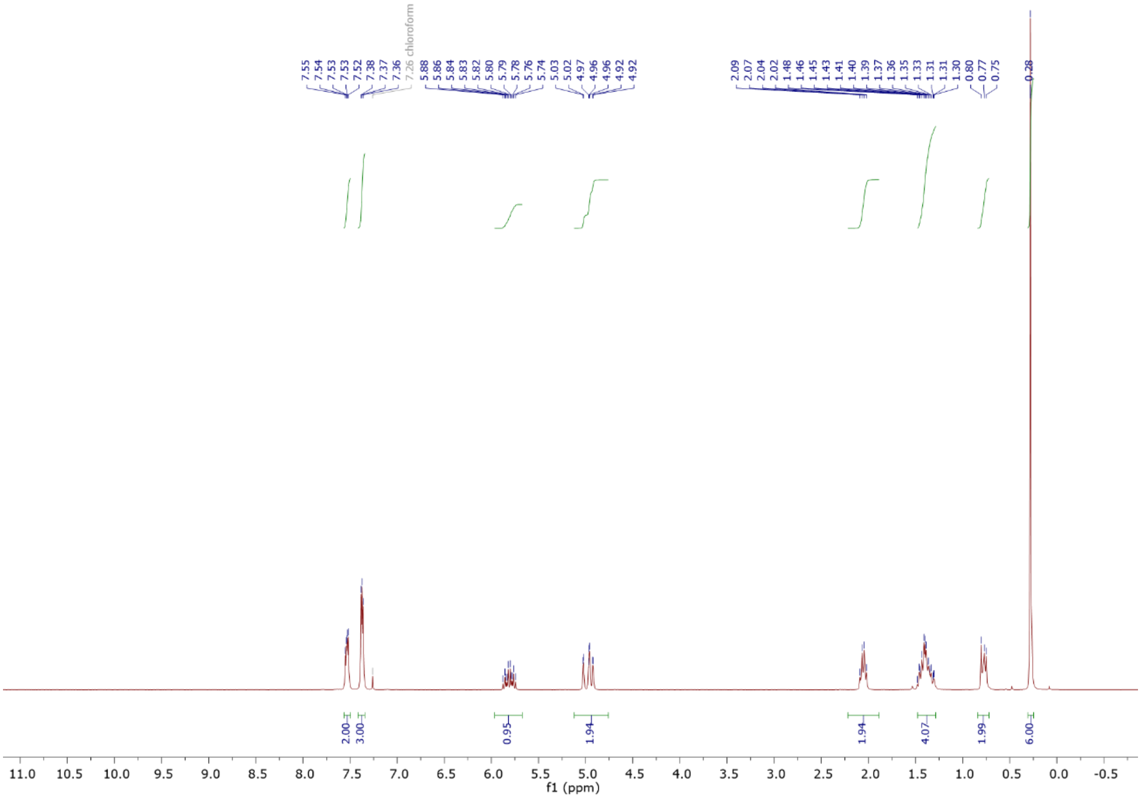

Figure S43. ^{1}H NMR spectrum of 4a

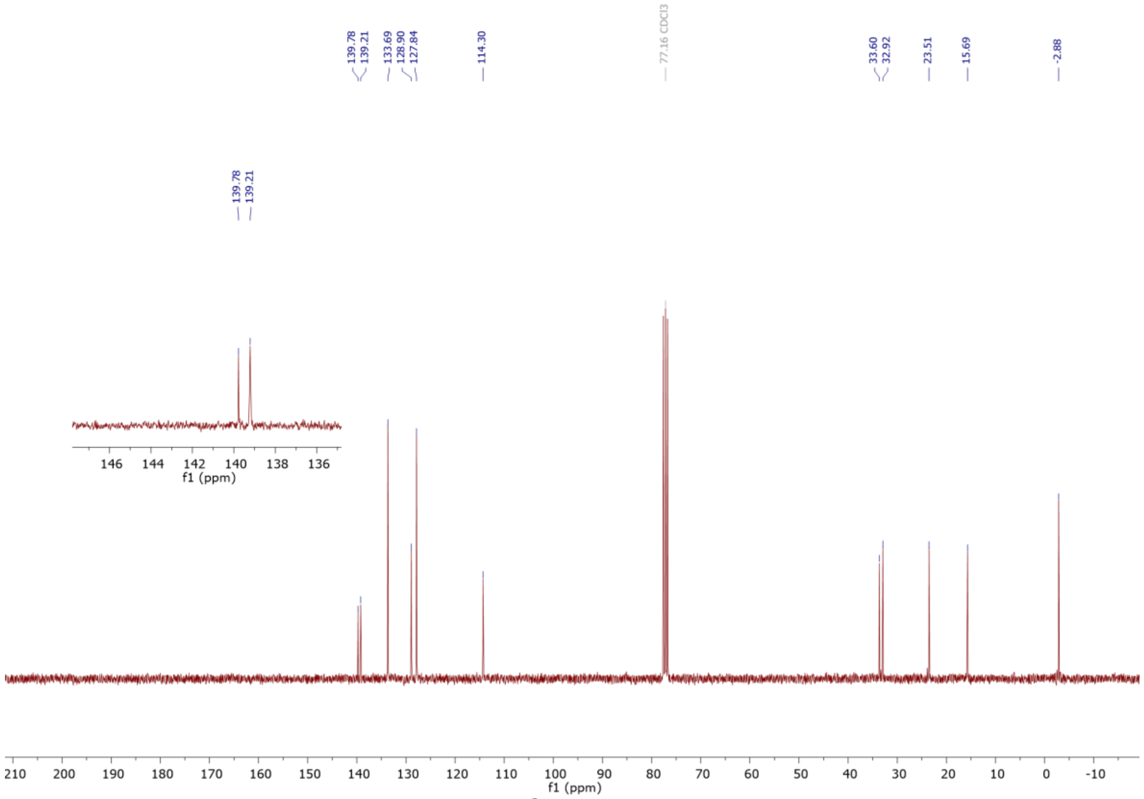

Figure S44. ^{13}C NMR spectrum of 4a

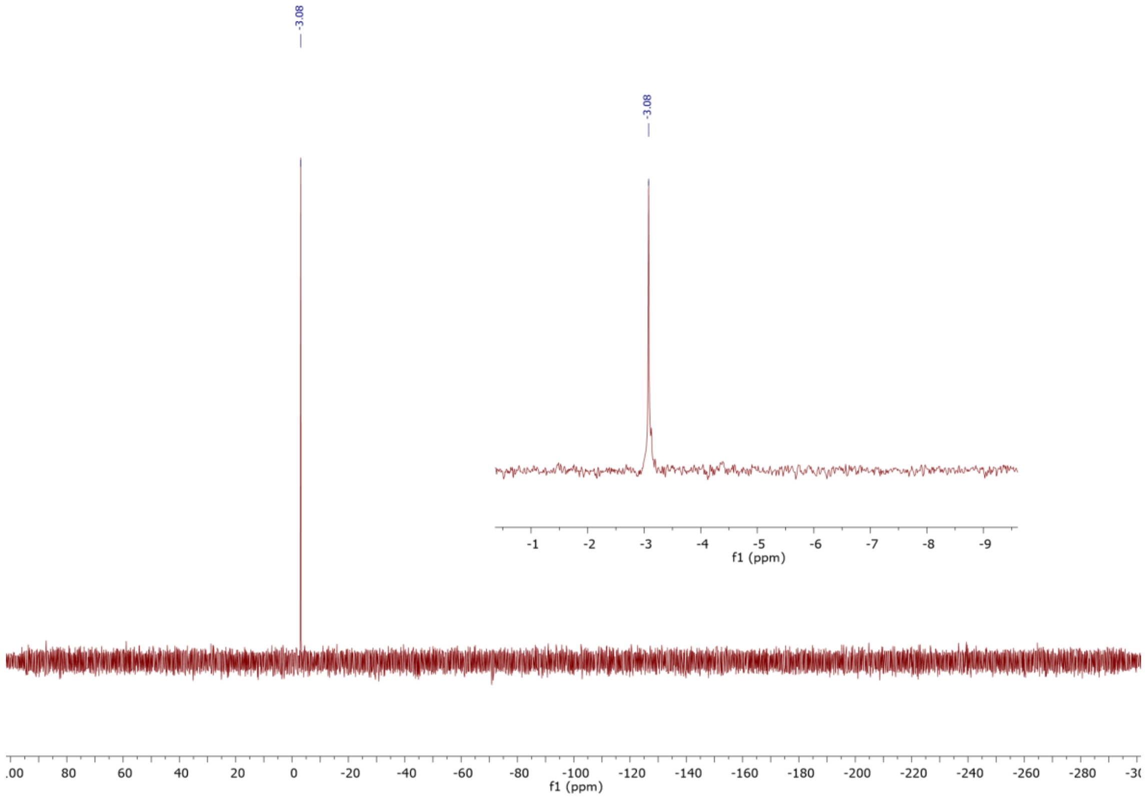

Figure S45. ^{29}Si NMR spectrum of 4a

2.16. Product 4b

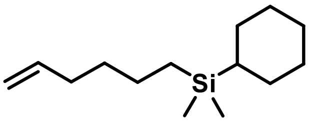

^{1}H NMR (300 MHz, $CDCl_3$) δ 5.81 CH_2=<u>CH</u>R (ddt, *J* = 16.9, 10.1, 6.7 Hz, 1H), 5.10 – 4.71 <u>CH_2</u>=CHR (m, 2H), 2.05 (q, *J* = 6.9 Hz, 2H), 1.68 (m, 5H), 1.42 – 1.00 (m, 9H), 0.66 – 0.37 SiCH, $SiCH_2$ (m, 3H), -0.10 $SiMe_2$ (s, 6H).
^{13}C NMR (75 MHz, $CDCl_3$) δ 139.34, 114.24, 33.68, 33.16, 28.32, 27.63, 27.20, 25.48, 23.60, 13.50, -5.19.
^{29}Si NMR (79 MHz, $CDCl_3$) δ 2.98.
MS (EI, m/z): 142.1 (9.2), 141.0 (71.9), 112.9 (32.3), 98.9 (32.8), 84.9 (11.8), 80.9 (24.7), 73.0 (87.5), 60.1 (7.1), 58.9 (100)
Product contains 4% of disubstituted derivative

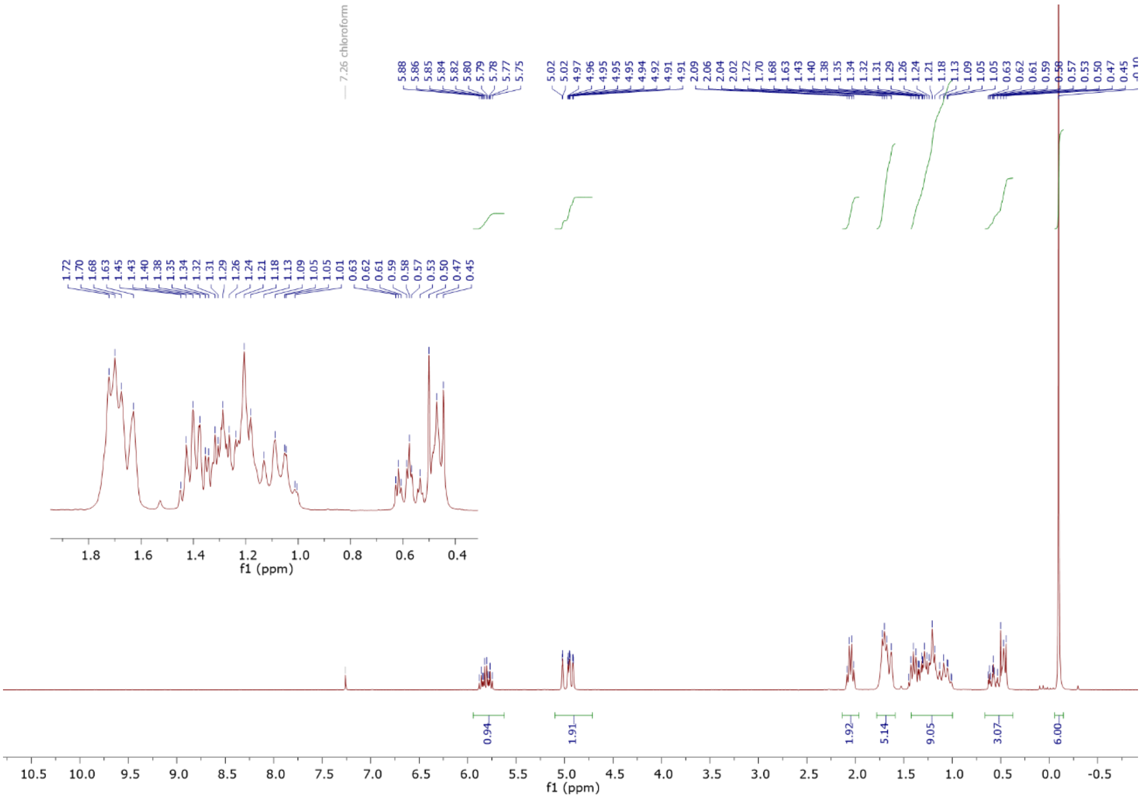

Figure S46. ^{1}H NMR spectrum of 4b

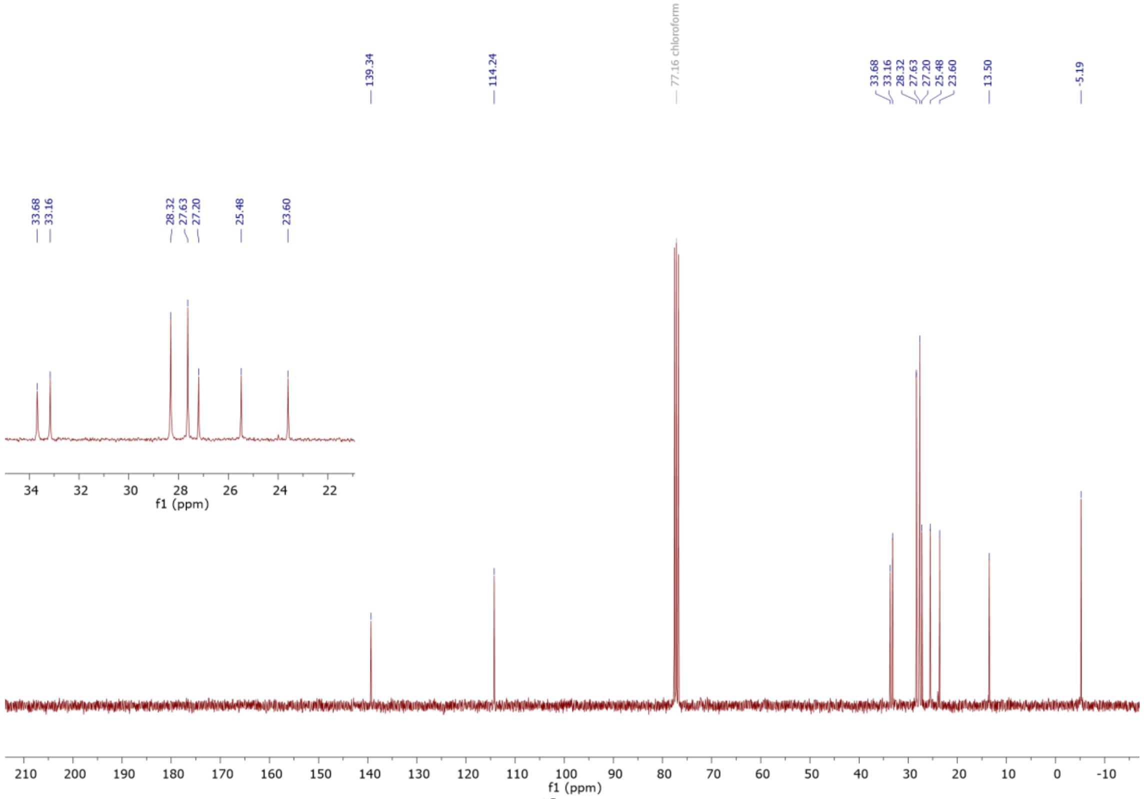

Figure S47. ^{13}C NMR spectrum of 4b

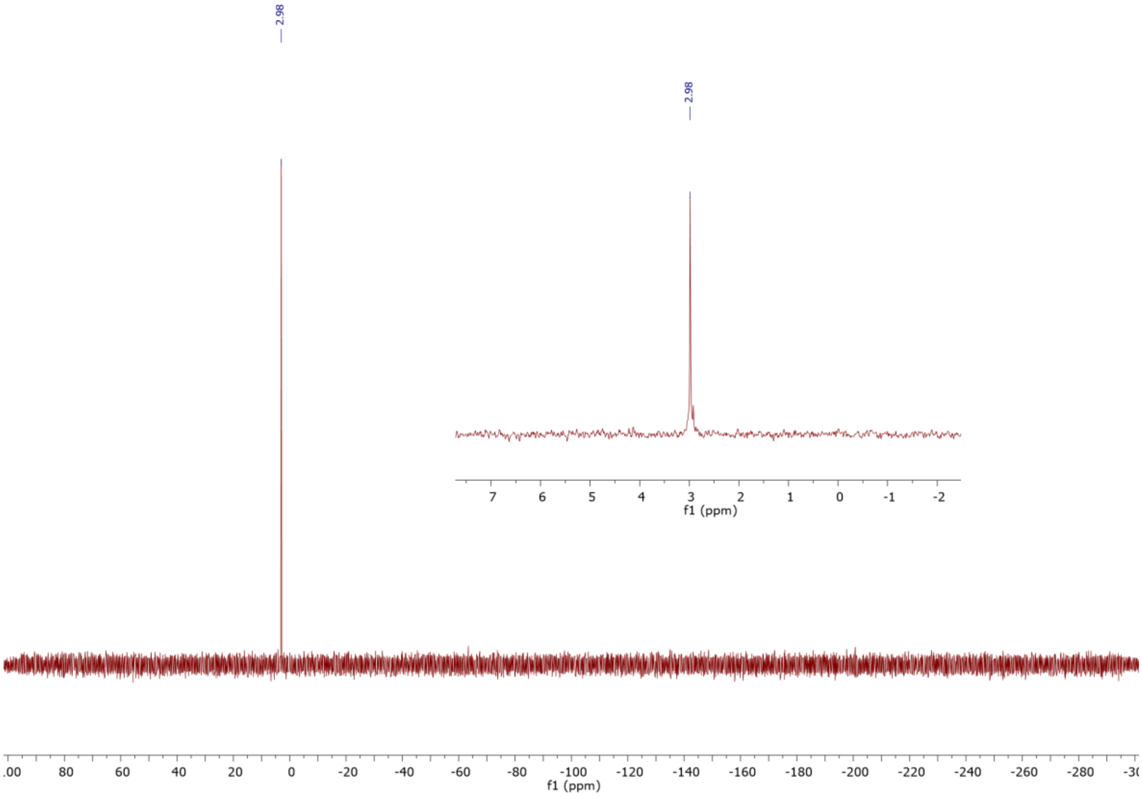

Figure S48. ^{29}Si NMR spectrum of 4b

2.17. Product 4c

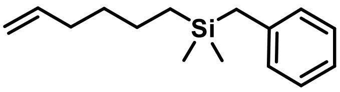

^{1}H NMR (300 MHz, $CDCl_3$) δ 7.22 (m, 2H), 7.05 (m, 3H), 5.82 CH_2=CHR (ddt, *J* = 16.9, 10.2, 6.7 Hz, 1H), 5.09 – 4.88 CH_2=CHR (m, 2H), 2.14 – 1.97 (m, 4H), 1.47 – 1.27 (m, 4H), 0.56 – 0.44 $SiCH_2$ (m, 2H), -0.02 $SiMe_2$ (s, 6H).
^{13}C NMR (75 MHz, $CDCl_3$) δ 140.60, 139.21, 128.26, 128.20, 123.97, 114.34, 33.62, 32.92, 25.75, 23.38, 14.73, -3.46.
^{29}Si NMR (79 MHz, $CDCl_3$) δ 2.29.
MS (EI, m/z): 216.9 $[M-15]^+$, 142.2 (8.9), 141.0 (70.3), 134.9 (6.5), 120.9 (16.2), 112.9 (32.1), 98.9 (25.2), 90.9 (5.8), 84.9 (15.3), 80.9 (14.6), 74.2 (6.0), 73.0 (80.4), 60.1 (7.0), 58.9 (100).
Product contains 3% of disubstituted derivative

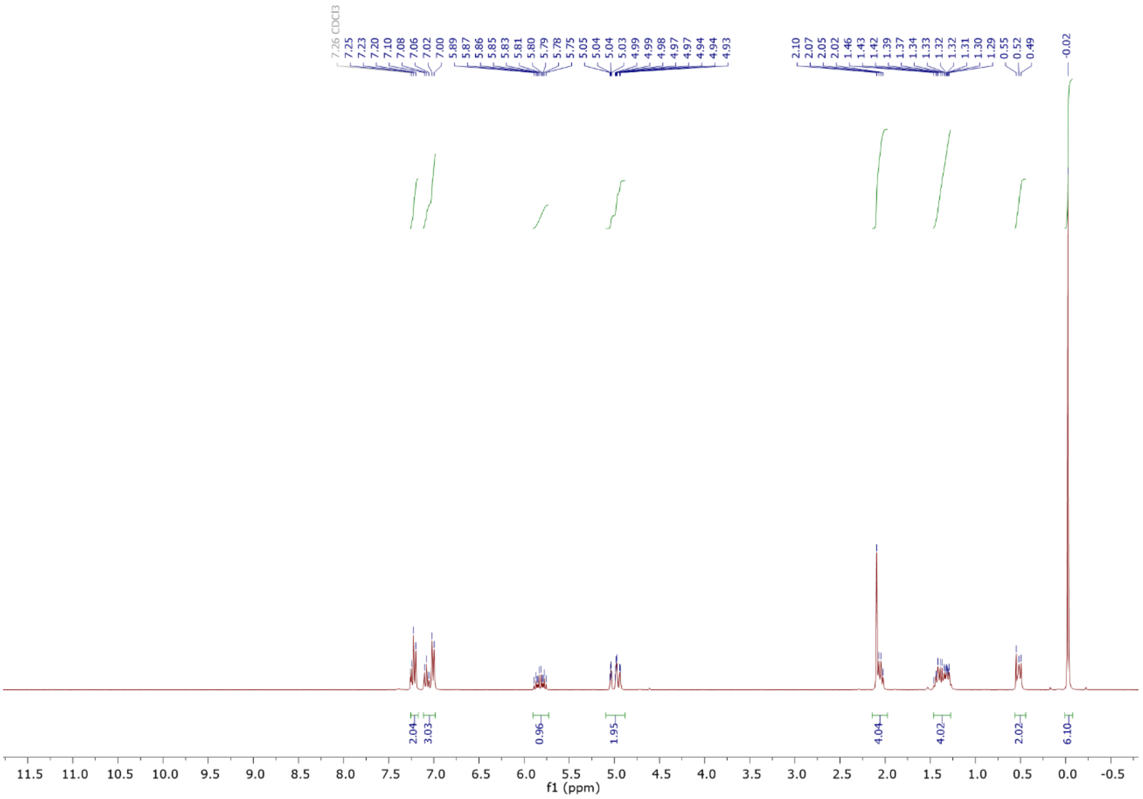

Figure S49. ^{1}H NMR spectrum of 4c

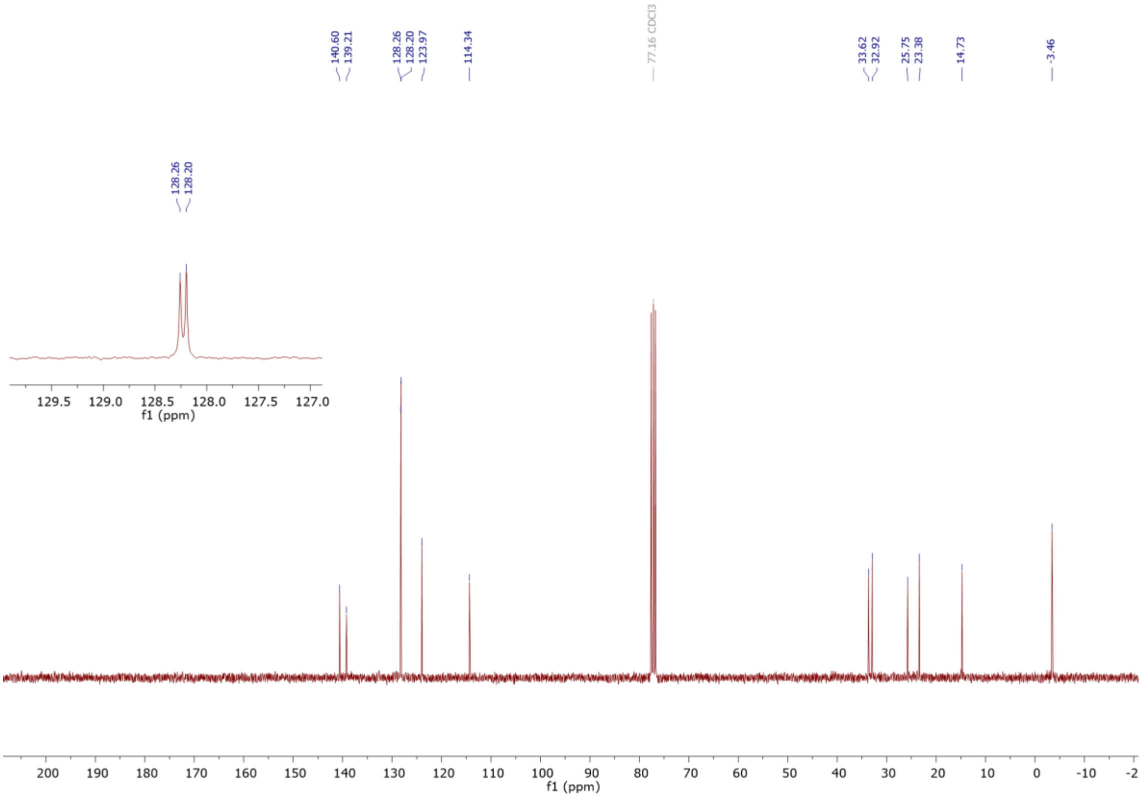

Figure S50. ^{13}C NMR spectrum of 4c

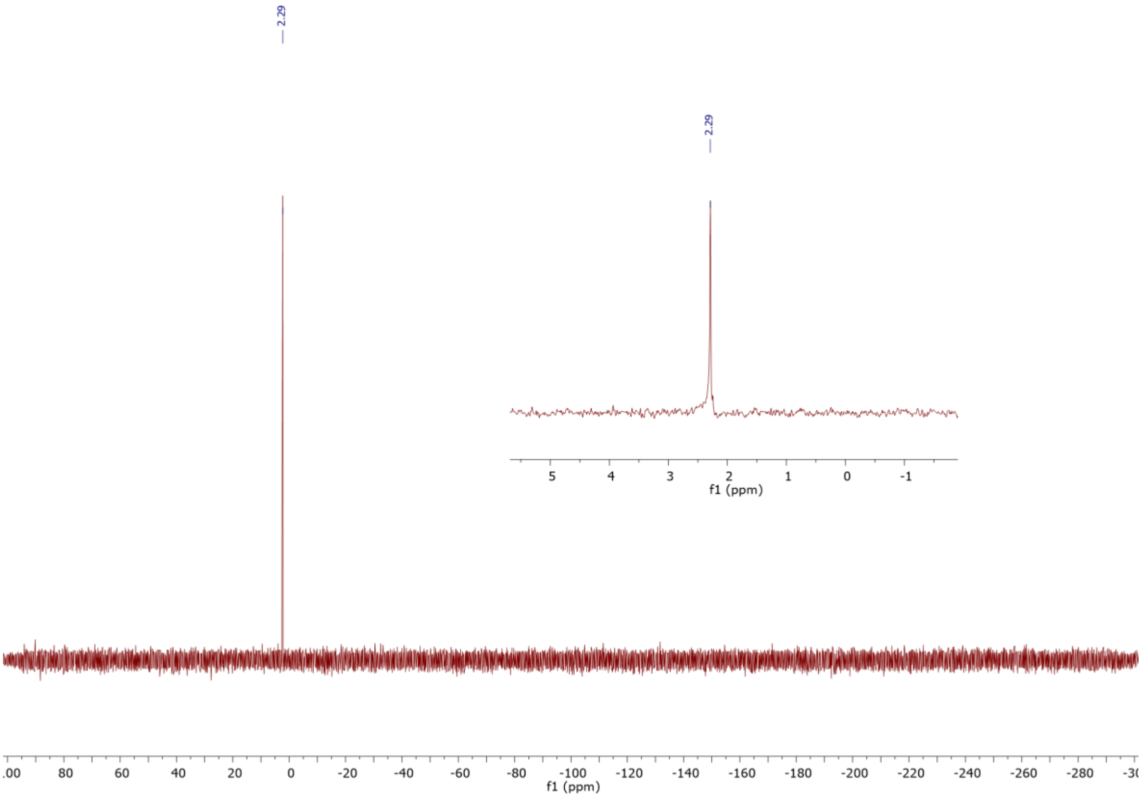

Figure S51. ^{29}Si NMR spectrum of 4c

2.18. Product 4d

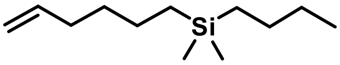

^{1}H NMR (300 MHz, $CDCl_3$) δ 5.81 CH_2=<u>CH</u>R (ddt, *J* = 16.9, 10.2, 6.7 Hz, 1H), 5.07 – 4.83 <u>CH_2</u>=CHR (m, 2H), 2.05 (q, *J* = 6.9 Hz, 2H), 1.45 – 1.20 (m, 8H), 0.88 CH_3 (t, *J* = 6.9 Hz, 3H), 0.55 – 0.44 $SiCH_2$ (m, 4H), -0.05 $SiMe_2$ (s, 6H).
^{13}C NMR (75 MHz, $CDCl_3$) δ 139.34, 114.24, 33.69, 33.05, 26.82, 26.31, 23.56, 15.28, 15.13, 13.99, -3.23.
^{29}Si NMR (79 MHz, $CDCl_3$) δ 2.27.
MS (EI, m/z): 142.2 (5.1), 141.0 (40.9), 126.9 (4.8), 114.9 (21.0), 112.9 (17.2), 98.9 (23.3), 84.9 (8.7), 80.9 (7.5), 74.2 (7.2), 73.0 (89.4), 60.1 (6.8), 58.9 (100).
Product contains 3% of disubstituted derivative

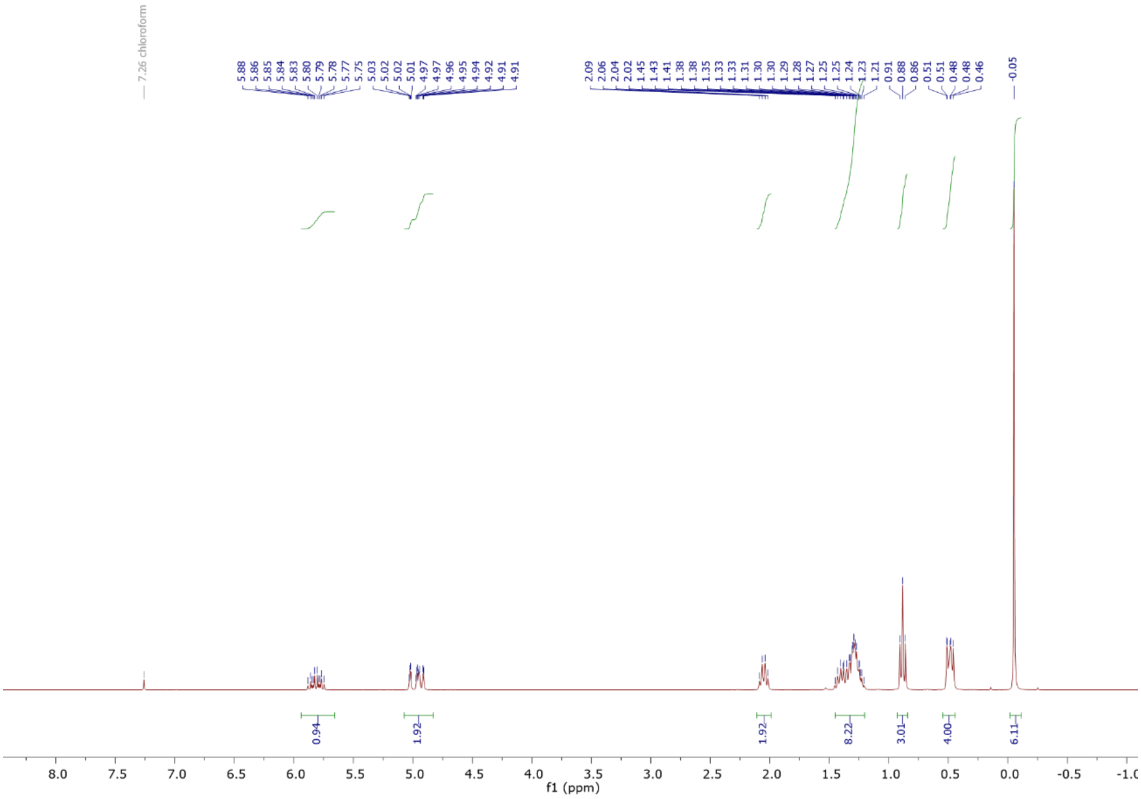

Figure S52. ^{1}H NMR spectrum of 4d

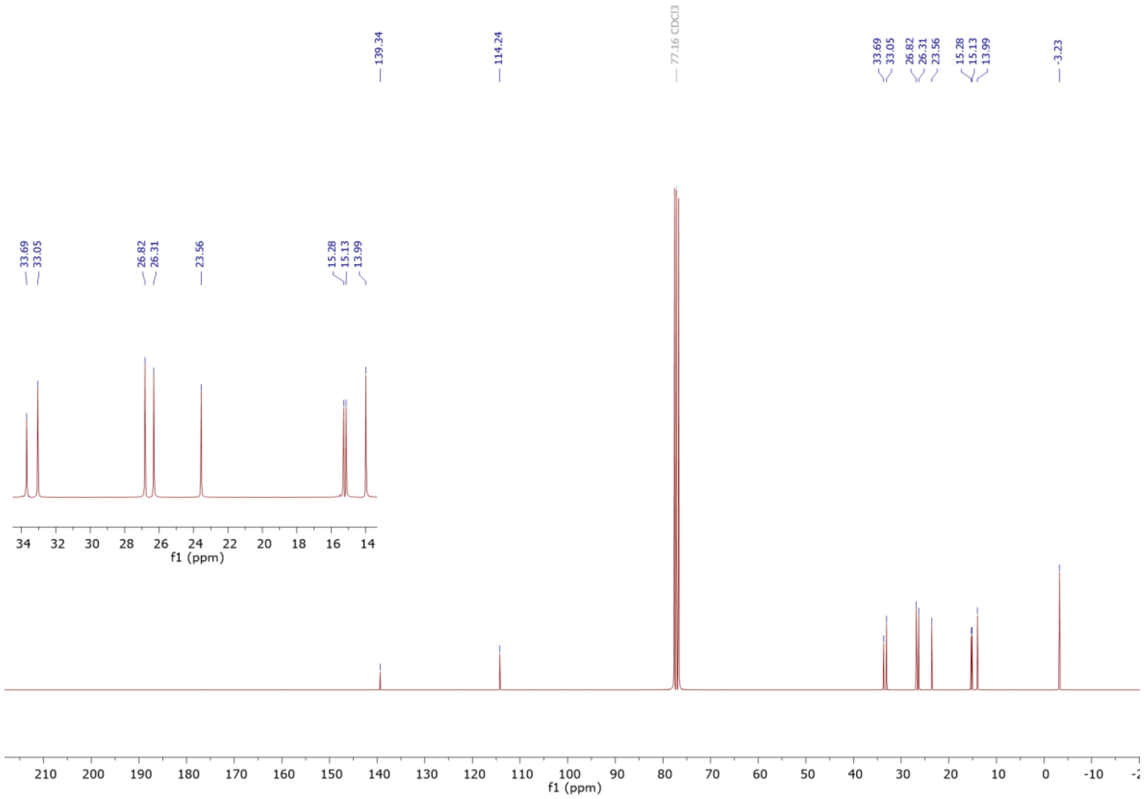

Figure S53. ^{13}C NMR spectrum of 4d

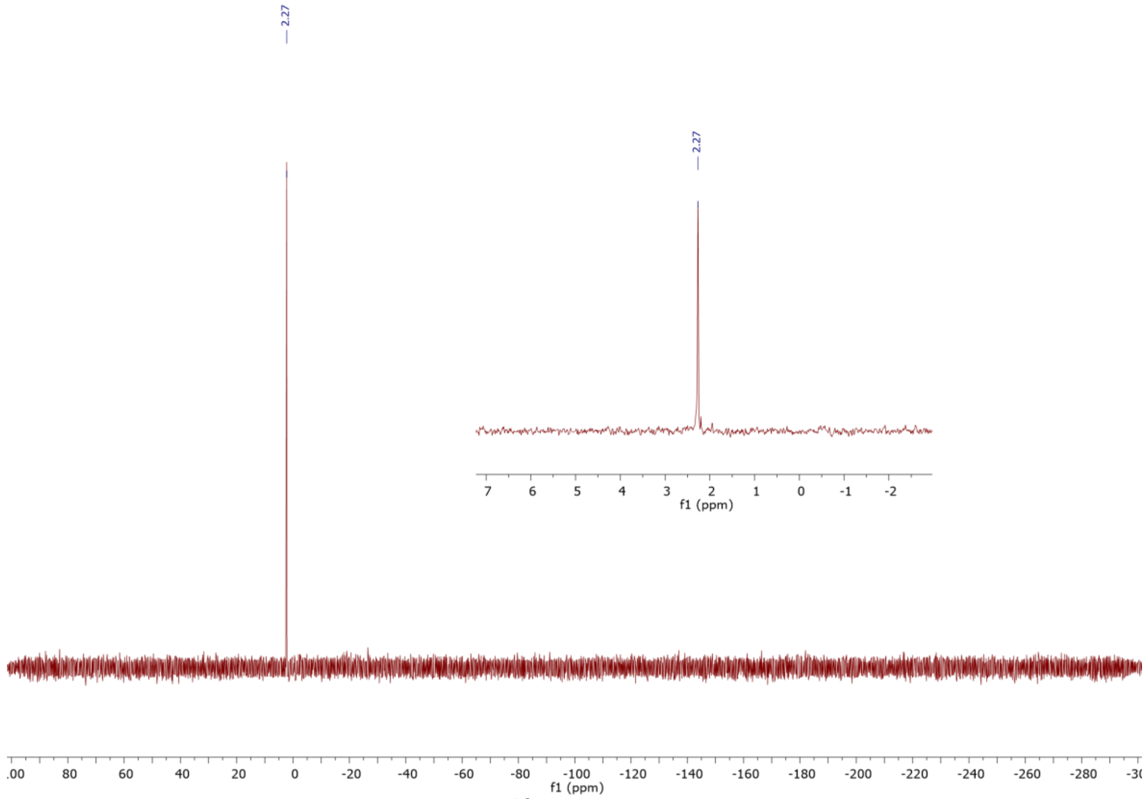

Figure S54. ^{29}Si NMR spectrum of 4d

2.19. Product 4e

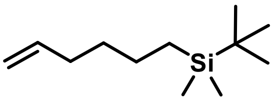

^{1}H NMR (300 MHz, $CDCl_3$) δ 5.81 CH_2=CHR (ddt, *J* = 16.9, 10.1, 6.7 Hz, 1H), 5.42 $CH_3CH=CHR_{isomer}$ (m, 2H) 5.09 – 4.86 CH_2=CHR (m, 2H), 2.05 (q, *J* = 6.9 Hz, 2H), 1.47 – 1.23 (m, 4H), 0.86 $C(CH_3)_3$ (s, 9H), 0.54 – 0.46 $SiCH_2$ (m, 2H), -0.08 $SiMe_2$ (s, 6H).
^{13}C NMR (75 MHz, $CDCl_3$) δ 139.32, 114.27, 33.67, 33.26, 26.76, 23.94, 16.69, 12.46, -6.14.
^{29}Si NMR (79 MHz, $CDCl_3$) δ 8.16.
MS (EI, m/z): 142.1 (5.2), 141.0 (36.0), 112.9 (29.1), 98.9 (23.7), 84.9 (15.2), 80.9 (13.0), 74.1 (7.7), 72.9 (100), 60.1 (6.5), 58.9 (88.3)

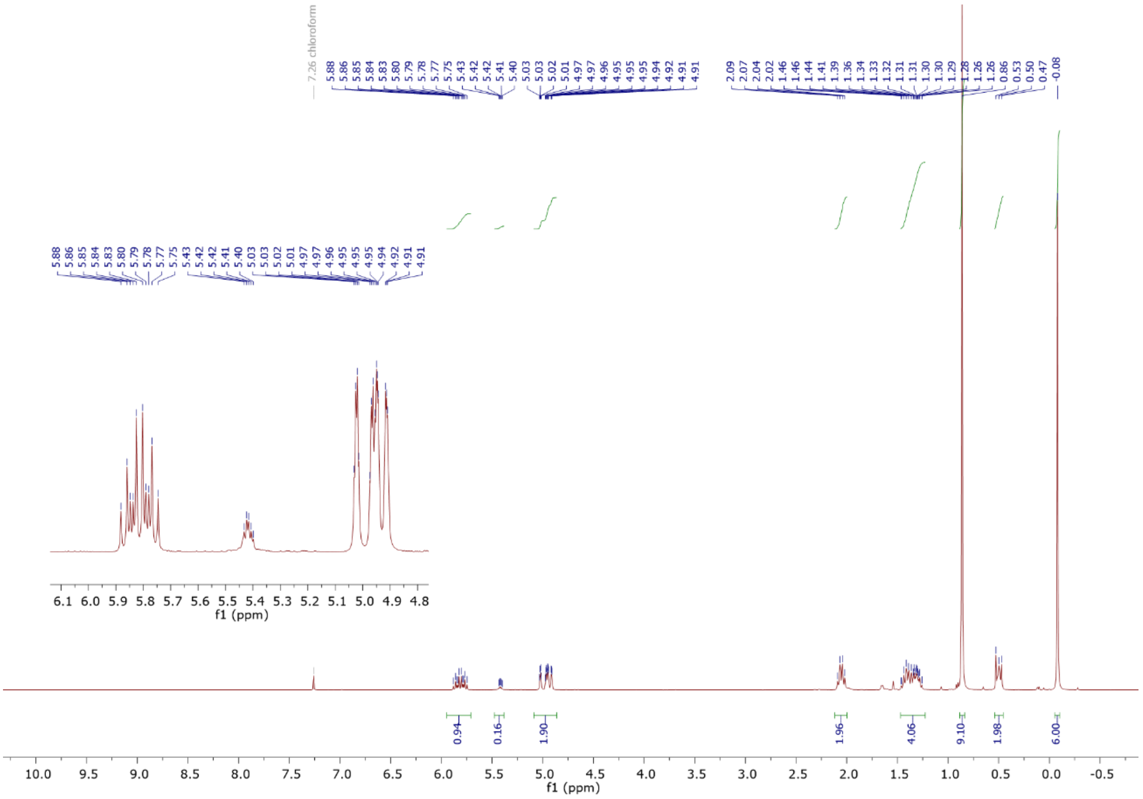

Figure S55. ^{1}H NMR spectrum of 4e

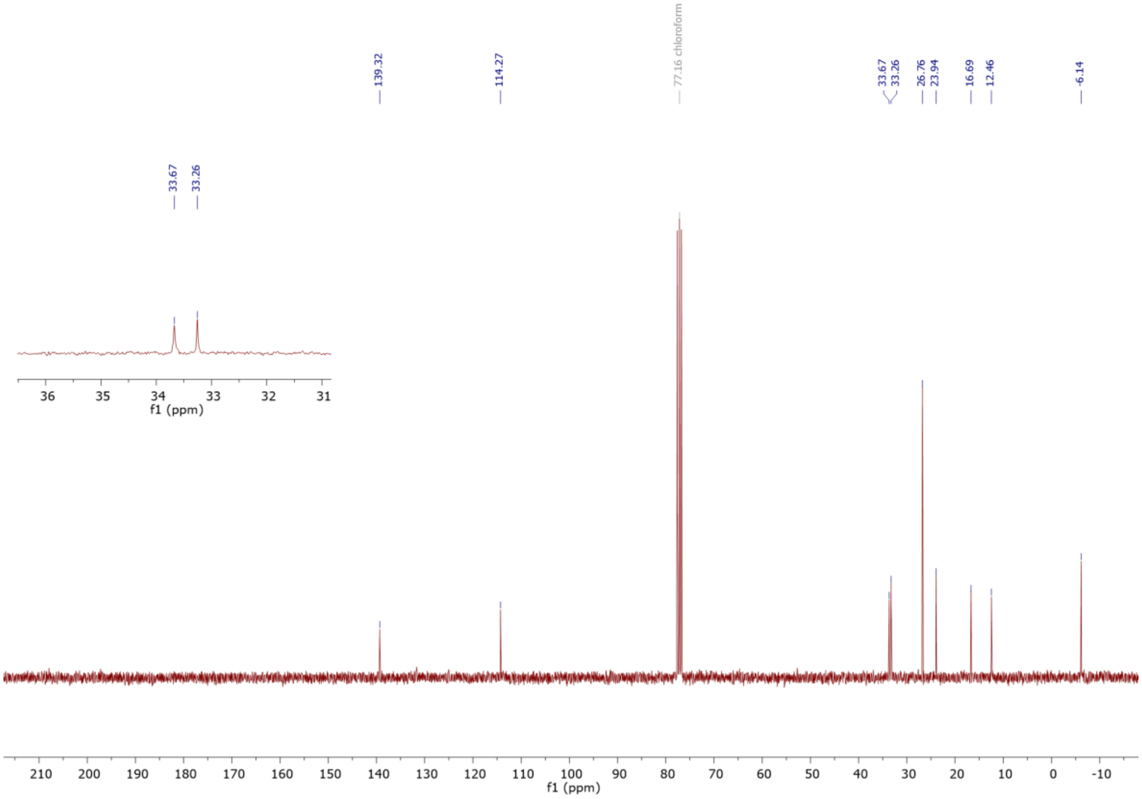

Figure S56. ^{13}C NMR spectrum of 4e

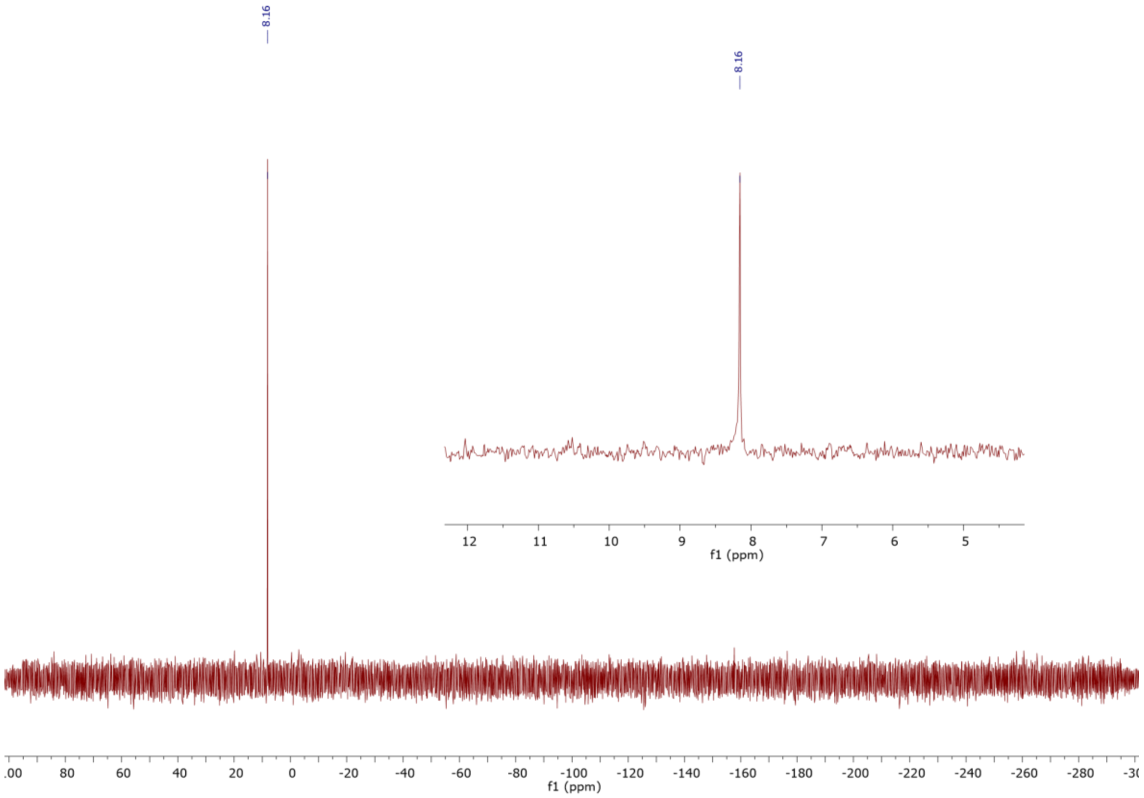

Figure S57. ^{29}Si NMR spectrum of 4e

2.20. Product 4f

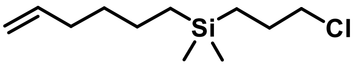

^{1}H NMR (300 MHz, $CDCl_3$) δ 5.80 CH_2=<u>CH</u>R (ddt, *J* = 16.9, 10.2, 6.7 Hz, 1H), 5.11 – 4.80 <u>CH_2</u>=CHR (m, 2H), 3.50 CH_2Cl (t, *J* = 7.0 Hz, 2H), 2.05 (q, *J* = 6.9 Hz, 2H), 1.82 – 1.69 (m, 2H), 1.45 – 1.25 (m, 4H), 0.64 – 0.55 $SiCH_2$ (m, 2H), 0.54 – 0.46 $SiCH_2$ (m, 2H), -0.02 $SiMe_2$ (s, 6H).
^{13}C NMR (101 MHz, $CDCl_3$) δ 139.06, 114.21, 48.07, 33.48, 32.80, 27.74, 23.28, 14.93, 12.96, -3.49.
^{29}Si NMR (79 MHz, $CDCl_3$) δ 2.78.
MS (EI, m/z): 137.1 (4.1), 134.9 (15.4), 118.8 (4.0), 112.9 (4.2), 94.8 (34.6), 93.9 (6.9), 92.8 (100), 80.9 (4.8), 72.9 (8.7), 58.9 (24.3)
Product contains 3% of disubstituted derivative

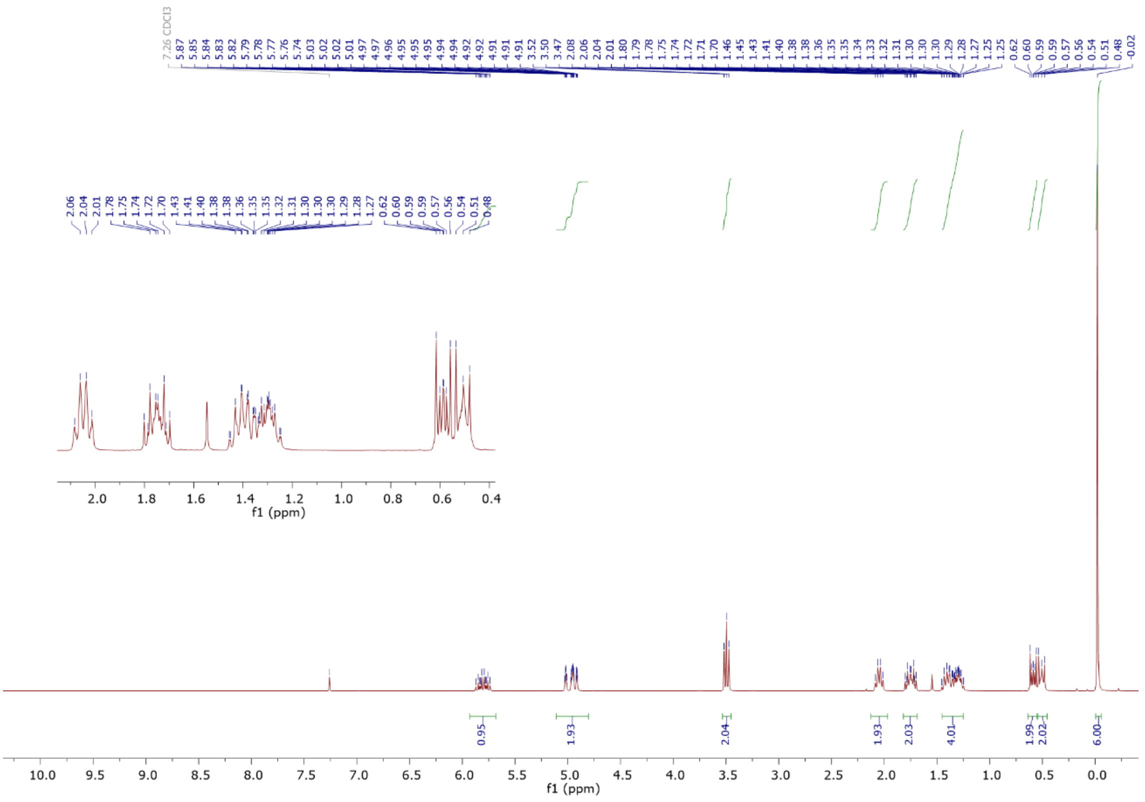

Figure S58. ^{1}H NMR spectrum of 4f

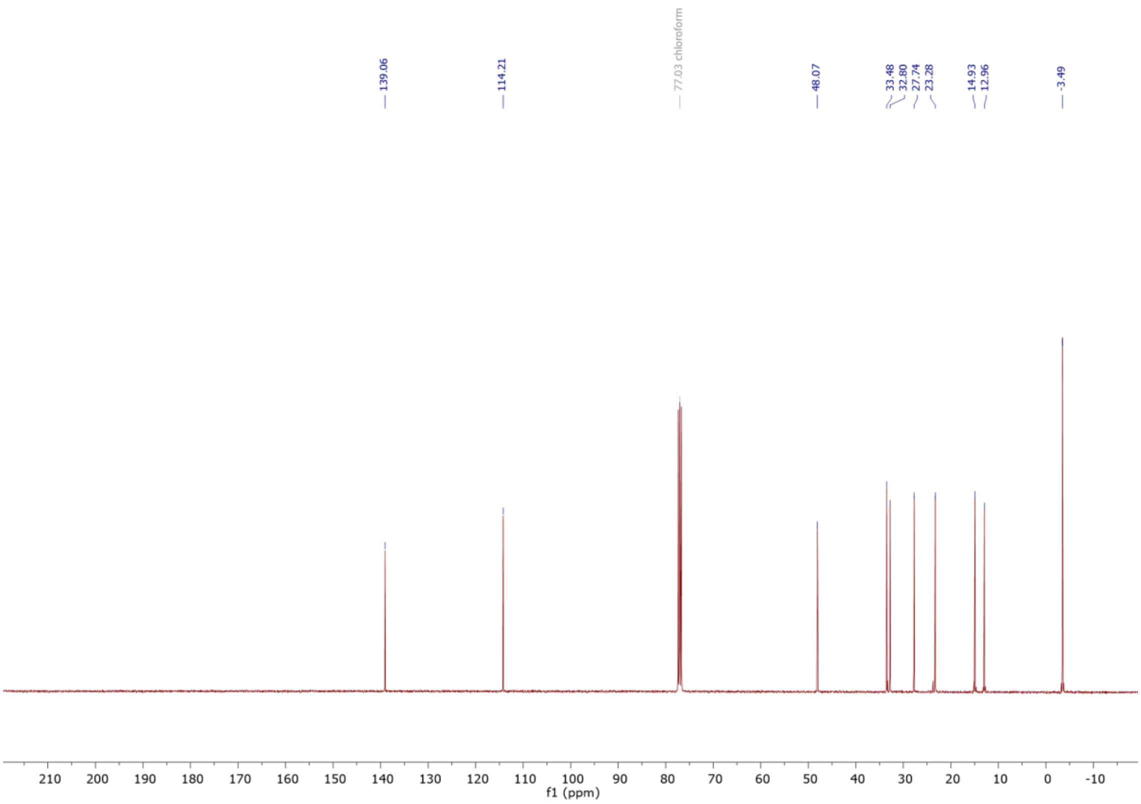

Figure S59. ^{13}C NMR spectrum of 4f

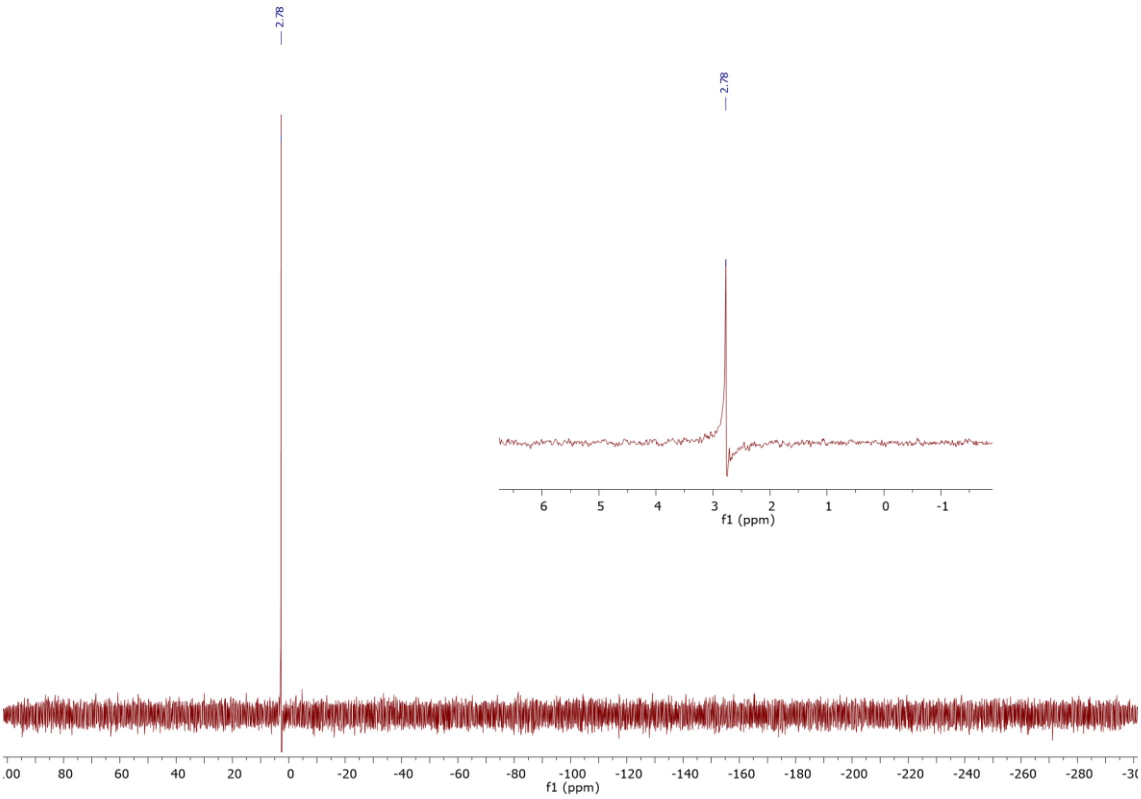

Figure S60. ^{29}Si NMR spectrum of 4f

2.21. Product 4g

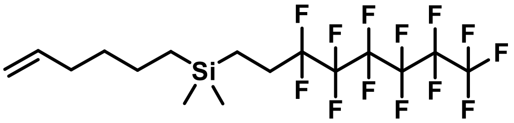

^{1}H NMR (300 MHz, $CDCl_3$) δ 5.80 CH_2=<u>CH</u>R (ddt, *J* = 16.9, 10.2, 6.7 Hz, 1H), 5.06 – 4.88 <u>CH_2</u>=CHR (m, 2H), 2.13 – 1.90 (m, 4H), 1.47 – 1.26 (m, 4H), 0.79 – 0.68 $SiMe_2$ (m, 2H), 0.59 – 0.49 $SiMe_2$ (m, 2H), 0.02 $SiMe_2$ (s, 6H).
^{13}C NMR (75 MHz, $CDCl_3$) δ 139.04, 114.47, 33.56, 32.82, 25.98, 23.23, 14.69, 4.54, -3.68.
^{29}Si NMR (79 MHz, $CDCl_3$) δ 3.76.
MS (EI, m/z): 308.9 (10.4), 288.9 (7.9), 262.9 (7.0), 244.9 (13.8), 238.9 (20.4), 194.9 (11.9), 138.9 (5.4), 116.9 (8.8), 112.9 (16.5), 102.9 (7.3), 98.9 (9.8), 88.9 (8.9), 84.9 (5.1), 80.9 (13.3), 78.2 (6.1), 76.9 (100), 73.0 (31.6)68.9 (10.3), 62.9 (7.8), 58.9 (48.1), 55.0 (14.4), 51.0 (14.4.

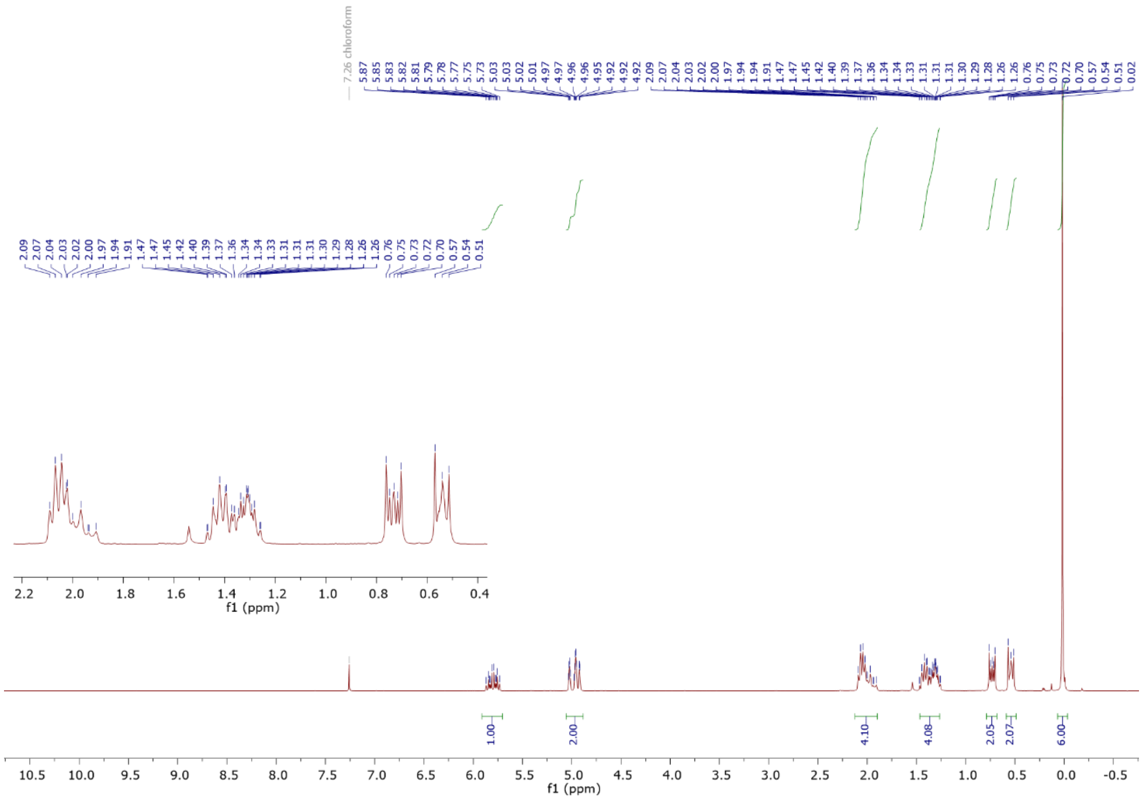

Figure S61. ^{1}H NMR spectrum of 4g

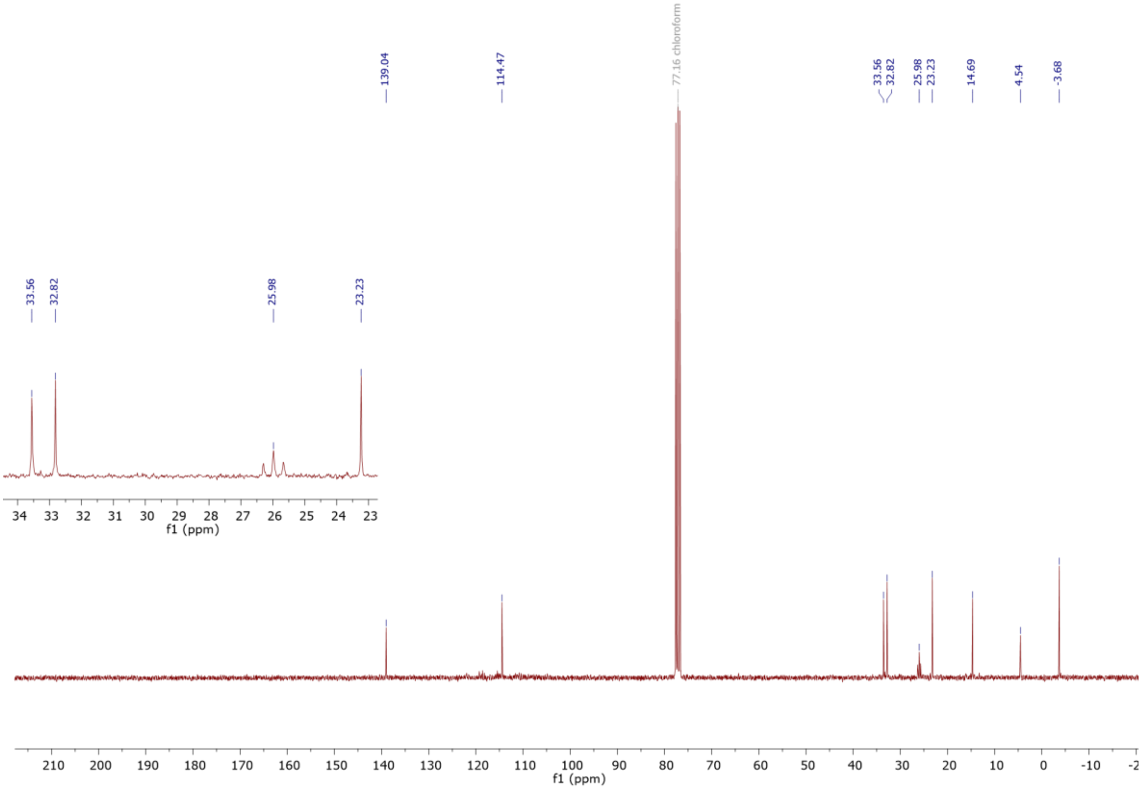

Figure S62. ^{13}C NMR spectrum of 4g

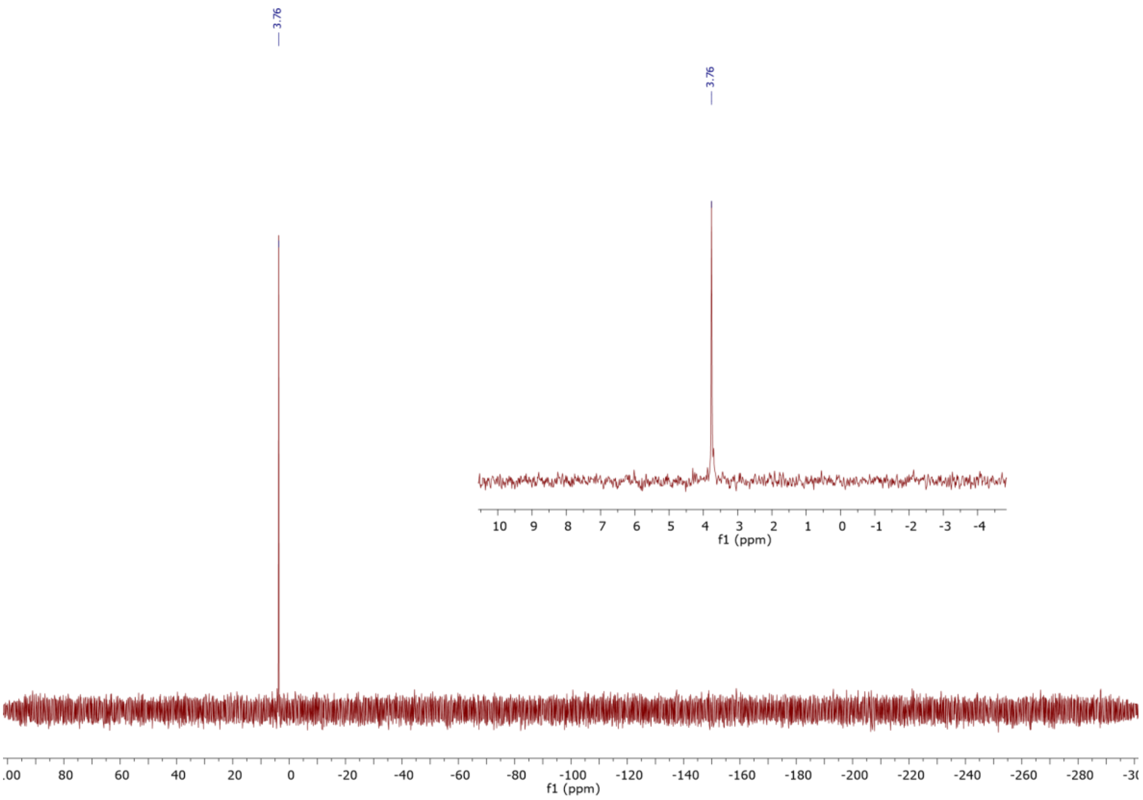

Figure S63. ^{29}Si NMR spectrum of 4g

2.22. Product 4h

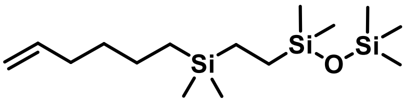

^{1}H NMR (300 MHz, $CDCl_3$) δ 5.81 CH_2=<u>CH</u>R (ddt, *J* = 16.9, 10.1, 6.7 Hz, 1H), 5.10 – 4.81 <u>CH_2</u>=CHR (m, 2H), 2.05 (q, *J* = 6.8 Hz, 2H), 1.46 – 1.28 (m, 4H), 0.56 – 0.46 $SiCH_2$ (m, 2H), 0.38 $SiCH_2$ (s, 4H), 0.03 $SiMe_2$ (s, 12H), -0.03 $SiMe_3$ (s, 9H).
^{13}C NMR (101 MHz, $CDCl_3$) δ 139.30, 114.27, 33.72, 32.78, 22.98, 18.42, 10.47, 8.22, 0.56, -0.21, -2.07.
^{29}Si NMR (79 MHz, $CDCl_3$) δ 8.12, 7.18, 3.07.
MS (EI, m/z): 316.1 M^+, 301.1 $[M-15]^+$, 233.0 (5.8), 215.0 (23.4), 146.9 (6.5), 144.9 (23.6), 141.0 (7.9), 135.0 (7.3), 134.1 (14.0), 132.9 (100), 116.9 (5.3), 72.9 (39.3), 58.9 (9.7)

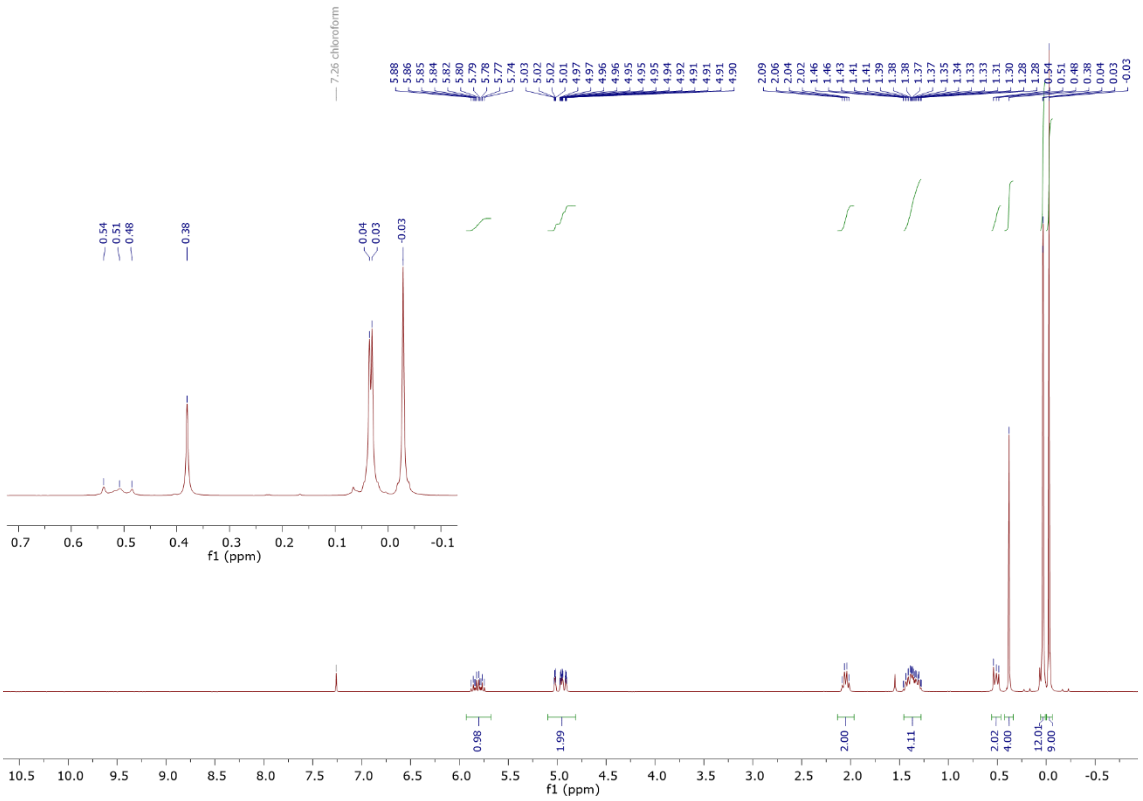

Figure S64. ^{1}H NMR spectrum of 4h

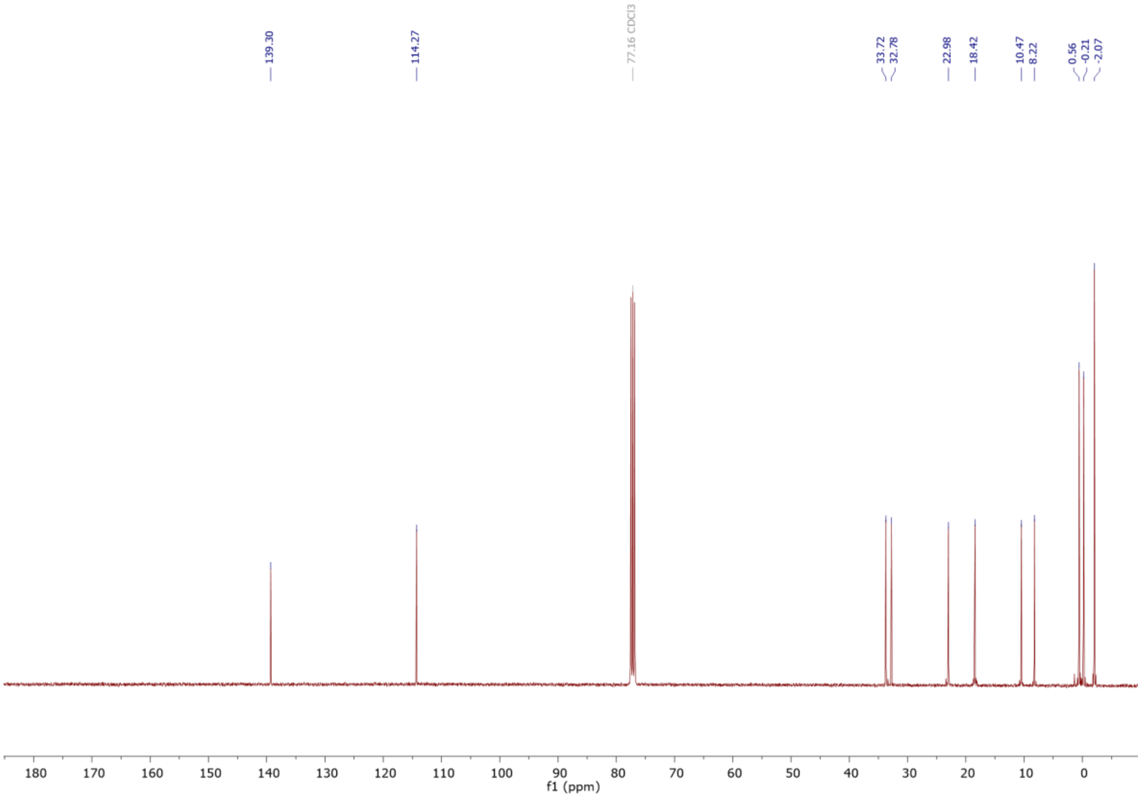

Figure S65. ^{13}C NMR spectrum of 4h

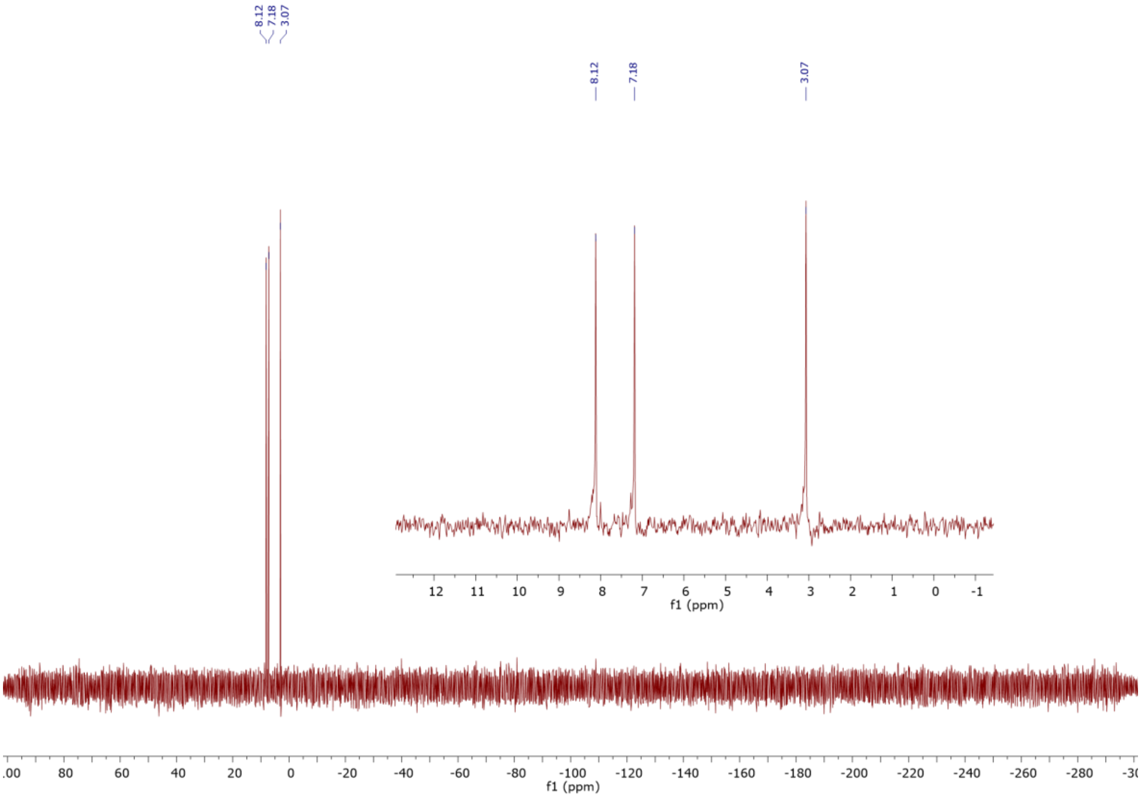

Figure S66. ^{29}Si NMR spectrum of 4h

2.23. Product 4i

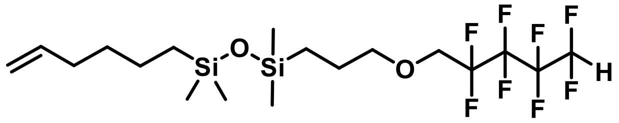

^{1}H NMR (300 MHz, $CDCl_3$) δ 6.35 – 5.63 CF_2H, CH_2=<u>CH</u>R (m, 2H), 5.05 – 4.88 <u>CH_2</u>=CHR (m, 2H), 3.92 (t, *J* = 14.0 Hz, 2H), 3.55 (t, *J* = 6.9 Hz, 2H), 2.05 (q, *J* = 6.9 Hz, 2H), 1.68 – 1.56 (m, 2H), 1.45 – 1.28 (m, 4H), 0.58 – 0.44 (m, 4H), 0.06 (s, 6H), 0.04 (s, 6H).
^{13}C NMR (101 MHz, $CDCl_3$) δ 139.26, 115.69, 114.27, 113.15, 110.32, 107.80, 105.28, 75.98, 67.64, 33.67, 32.72, 23.48, 22.92, 18.33, 14.12, 0.45, 0.38.
^{29}Si NMR (79 MHz, $CDCl_3$) δ 7.87, 7.23.
MS (EI, m/z): 473.1 $[M-15]^+$, 153.0 (7.5), 152.1 (13.5), 150.9 (100), 136.9 (13.5), 132.9 (32.5), 72.9 (6.5),

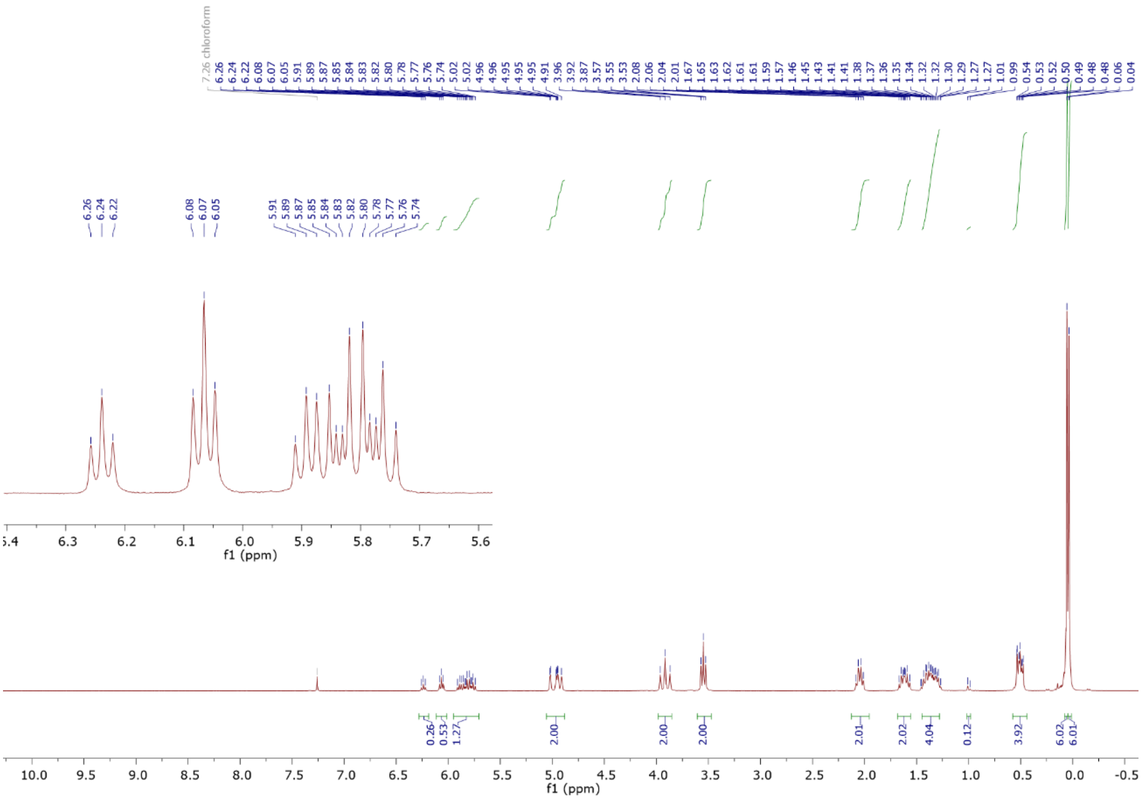

Figure S67. ^{1}H NMR spectrum of 4i

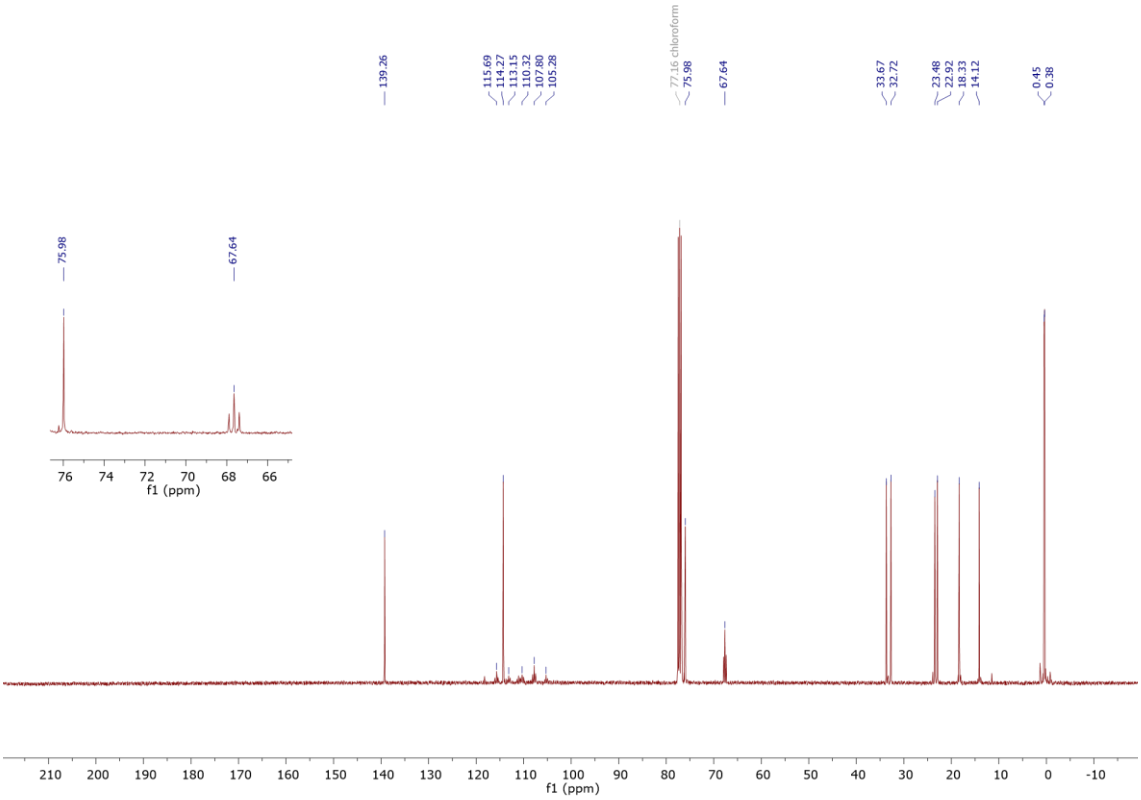

Figure S68. ^{13}C NMR spectrum of 4i

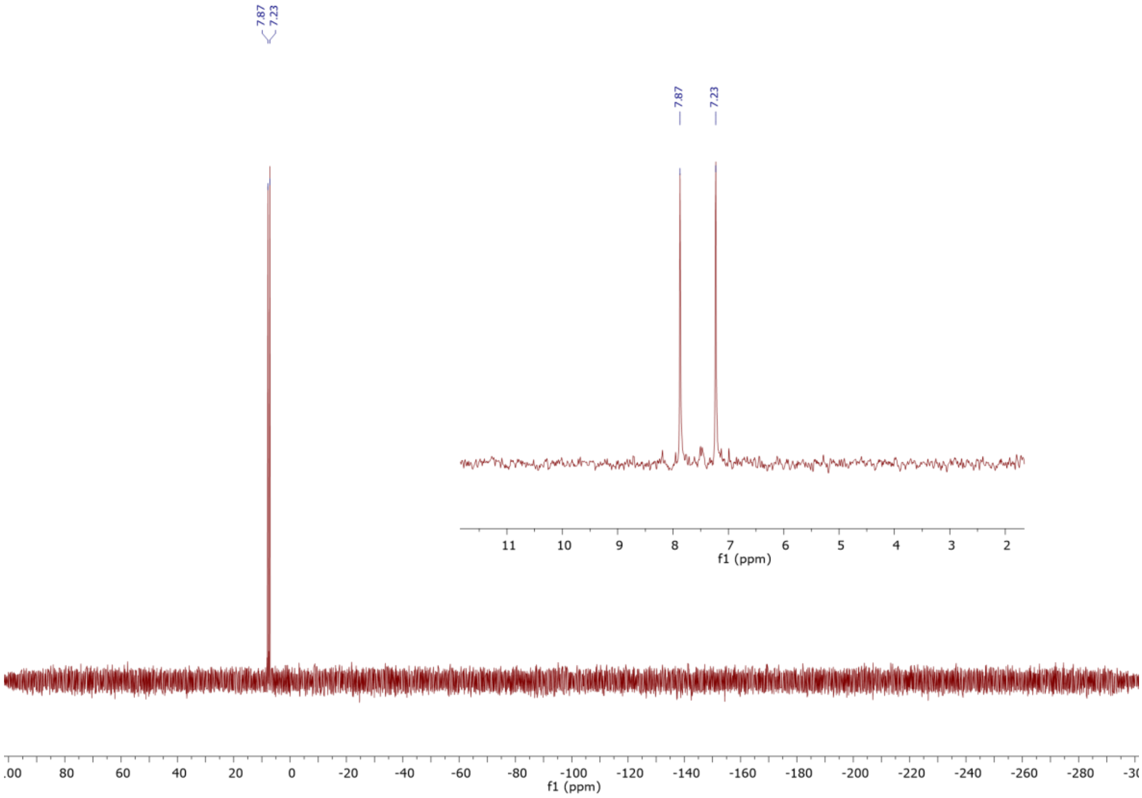

Figure S69. ^{29}Si NMR spectrum of 4i

2.24. Product 4j

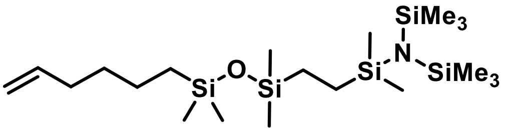

^{1}H NMR (300 MHz, $CDCl_3$) δ 5.81 CH_2=<u>CH</u>R (ddt, *J* = 16.9, 10.2, 6.7 Hz, 1H), 5.05 – 4.88 <u>CH_2</u>=CHR (m, 2H), 2.05 (q, *J* = 6.8 Hz, 2H), 1.45 – 1.29 (m, 4H), 0.55 – 0.46 $SiCH_2$ (m, 4H), 0.44 – 0.35 $SiCH_2$ (m, 2H), 0.18 $SiMe_3$ (s, 18H), 0.16 $SiMe_2$ (s, 6H), 0.04 $SiMe_2$ (s, 12H).
^{13}C NMR (75 MHz, $CDCl_3$) δ 139.30, 114.27, 33.72, 32.79, 22.99, 18.43, 12.14, 10.79, 5.74, 3.19, 0.57, -0.15.
^{29}Si NMR (79 MHz, $CDCl_3$) δ 8.11, 7.27, 5.07, 2.23.
MS (EI, m/z): 461.2 M^+, 220.1 (11.7), 219.1 (23.0), 218.0 (100), 216.0 (11.8), 202.0 (6.1), 132.9 (17.2), 129.9 (6.8), 72.9 (6.8), .

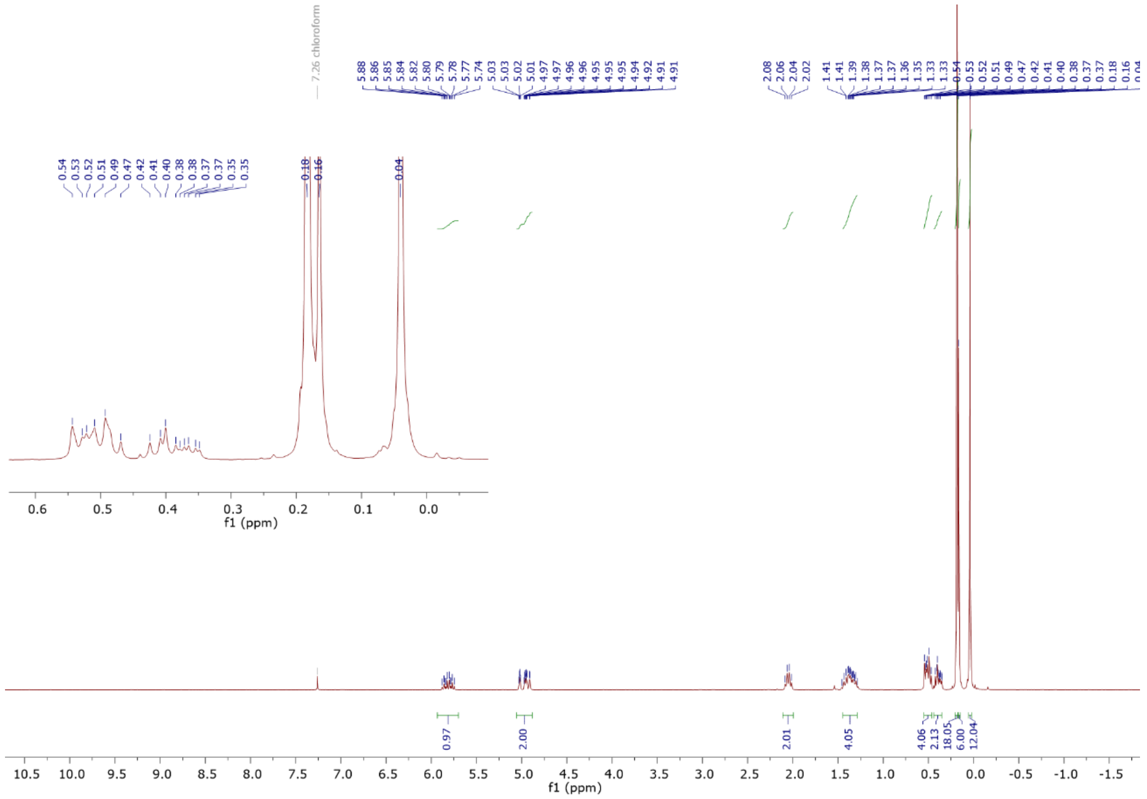

Figure S70. ^{1}H NMR spectrum of 4j

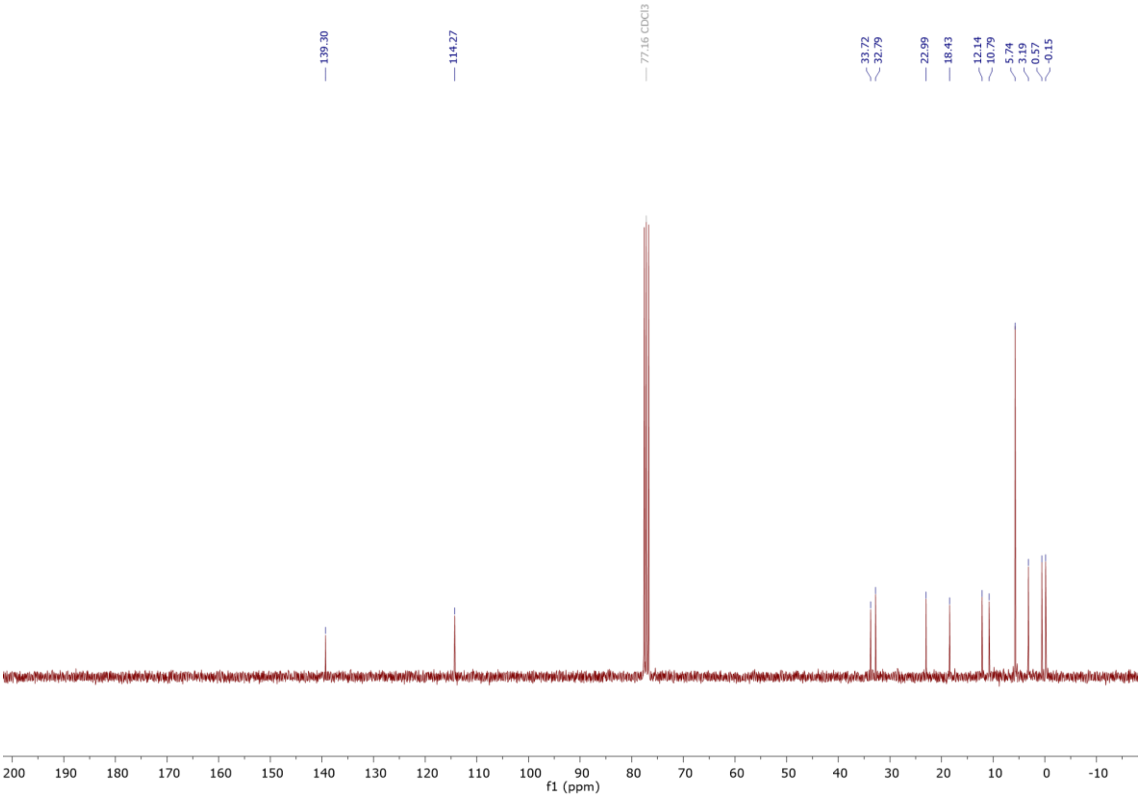

Figure S71. ^{13}C NMR spectrum of 4j

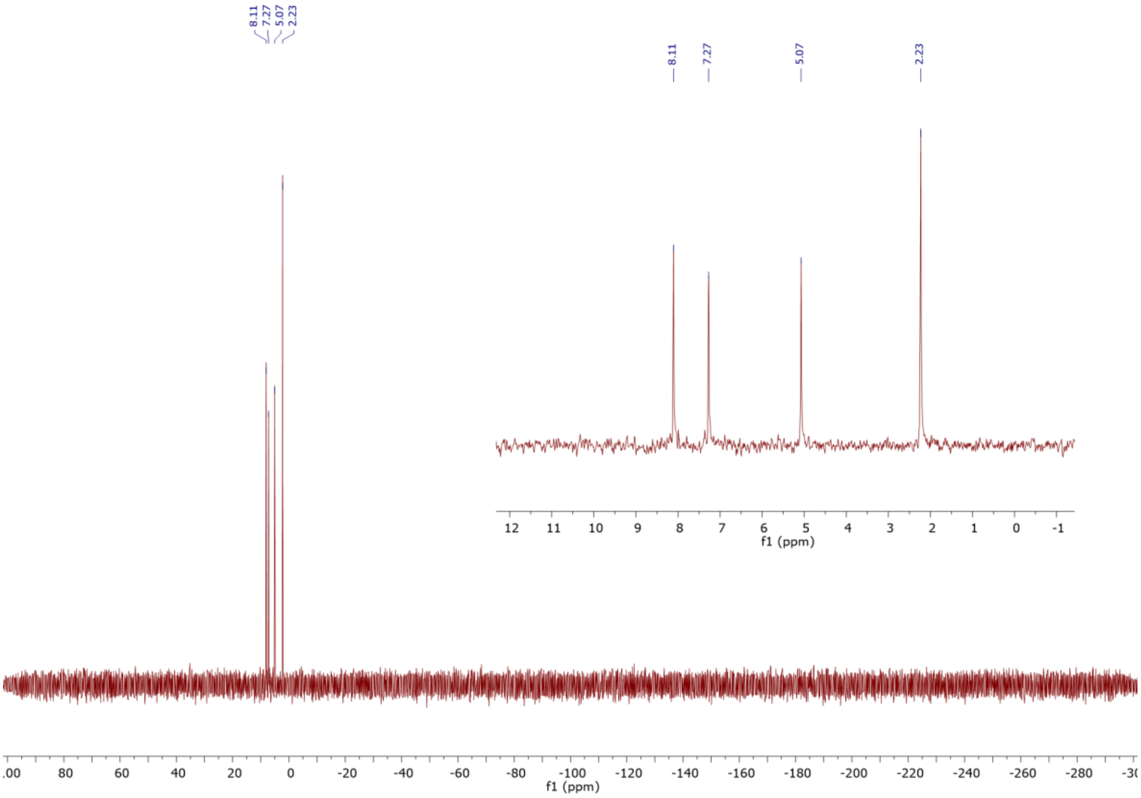

Figure S72. ^{29}Si NMR spectrum of 4j

2.25. Product 4k

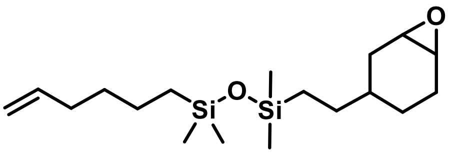

^{1}H NMR (300 MHz, $CDCl_3$) δ 5.81 CH_2=<u>CH</u>R (ddt, *J* = 16.9, 10.1, 6.7 Hz, 1H), 5.06 – 4.86 <u>CH_2</u>=CHR (m, 2H), 3.21 – 3.05 (m, 2H), 2.22 – 1.64 (m, 5H), 1.54 – 0.82 (m, 10H), 0.58 – 0.37 $SiCH_2$ (m, 4H), 0.02 $SiMe_2$ (s, 12H).
^{13}C NMR (75 MHz, $CDCl_3$) δ 139.25, 114.29, 53.44, 52.92, 52.17, 52.13, 35.54, 33.69, 32.73, 32.42, 31.70, 30.56, 30.33, 29.77, 26.91, 25.54, 24.21, 23.74, 22.93, 18.36, 15.40, 15.27, 0.53, 0.38.
^{29}Si NMR (79 MHz, $CDCl_3$) δ 7.50, 7.42, 7.40.
MS (EI, m/z): 325.0 $[M-15]^+$, 151.0 (7.2), 150.1 (13.4), 148.9 (100), 134.9 (16.2), 134.0 (9.7), 132.9 (67.7), 118.8 (6.0), 108.9 (7.6), 80.9 (12.1), 80.0 (5.9), 78.9 (6.7), 72.9 (8.3), 66.9 (24.2).

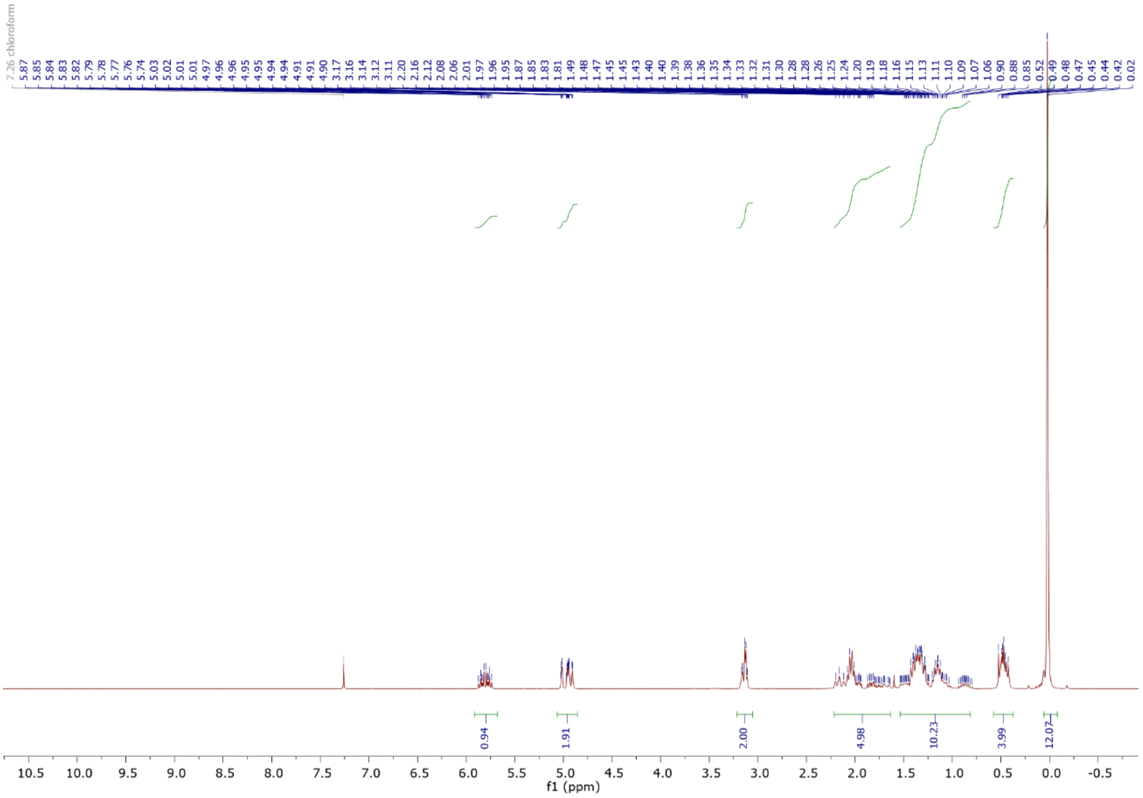

Figure S73. ^{1}H NMR spectrum of 4k

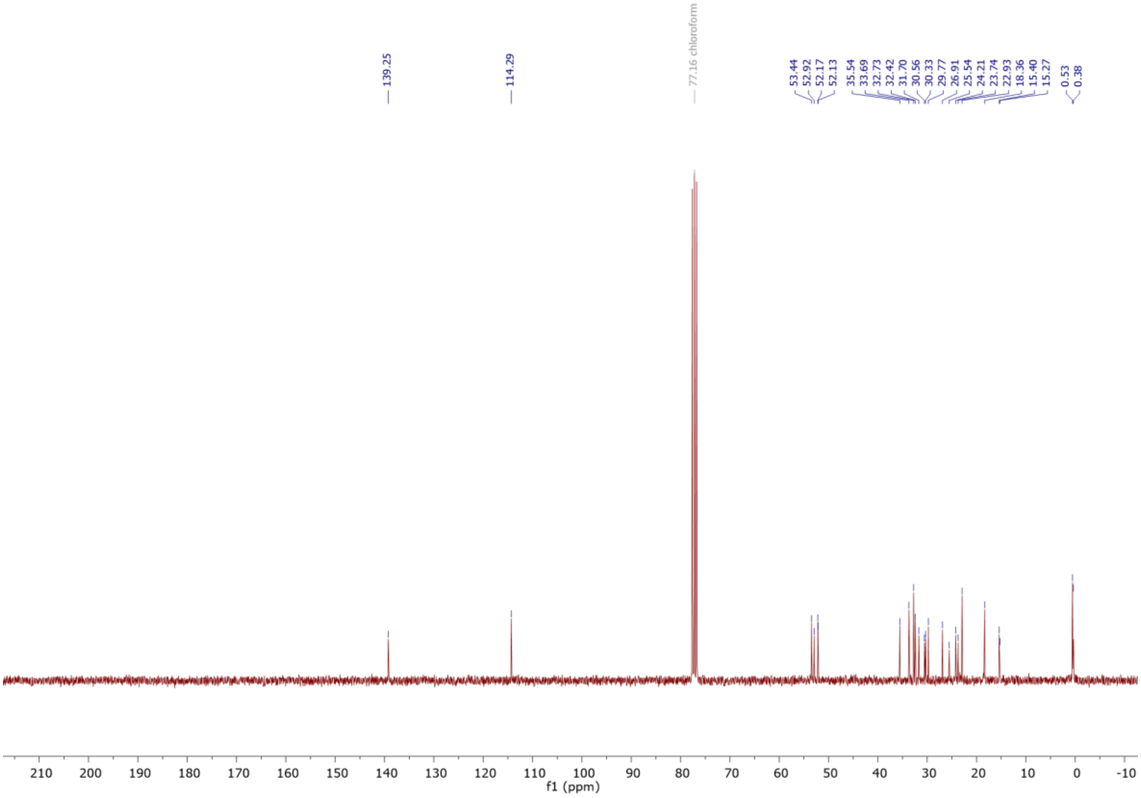

Figure S74. ^{13}C NMR spectrum of 4k

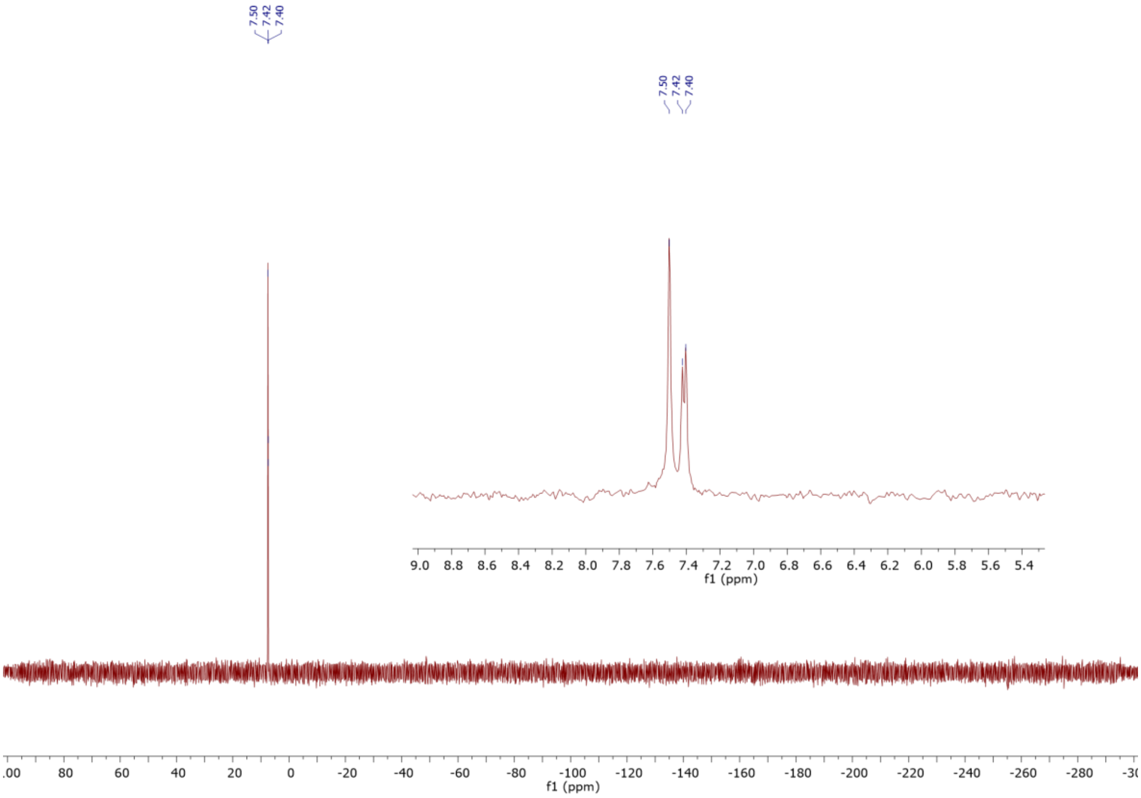

Figure S75. ^{29}Si NMR spectrum of 4k

2.26. Product 4l

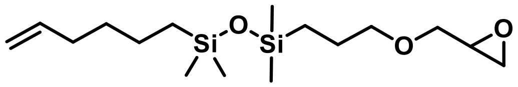

^{1}H NMR (300 MHz, $CDCl_3$) δ 5.80 CH_2=<u>CH</u>R (ddt, *J* = 16.9, 10.1, 6.7 Hz, 1H), 5.05 – 4.86 <u>CH_2</u>=CHR (m, 2H), 3.70 (dd, *J* = 11.5, 3.1 Hz, 1H), 3.54 – 3.31 (m, 3H), 3.20 – 3.08 (m, 1H), 2.85 – 2.75 (m, 1H), 2.61 (dd, *J* = 5.0, 2.7 Hz, 1H), 2.04 (q, *J* = 6.8 Hz, 2H), 1.66 – 1.54 (m, 2H), 1.45 – 1.26 (m, 4H), 0.57 – 0.43 $SiCH_2$ (m, 4H), 0.04 $SiMe_2$ (s, 12H).
^{13}C NMR (75 MHz, $CDCl_3$) δ 139.25, 114.28, 74.53, 71.57, 51.03, 44.52, 33.67, 32.71, 23.63, 22.91, 18.33, 14.37, 0.50, 0.42.
^{29}Si NMR (79 MHz, $CDCl_3$) δ 7.66, 7.38.
MS (EI, m/z): 315.1 $[M-15]^+$, 177.1 (6.4), 176.1 (12.0), 174.9 (76.7), 148.9 (15.1), 135.0 (11.1), 134.1 (13.6), 132.9 (100), 130.9 (8.4), 118.8 (5.1), 116.9 (5.1), 72.9 (7.4), 57.0 (5.4).

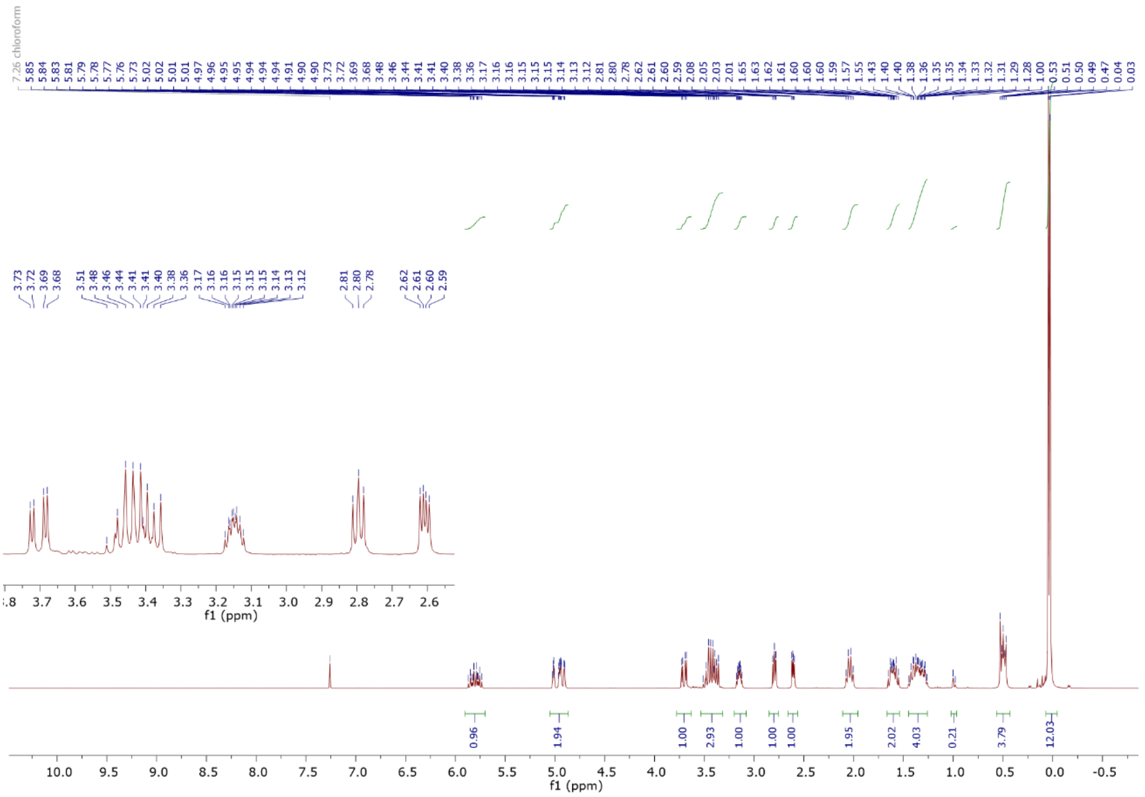

Figure S76. ^{1}H NMR spectrum of 4l

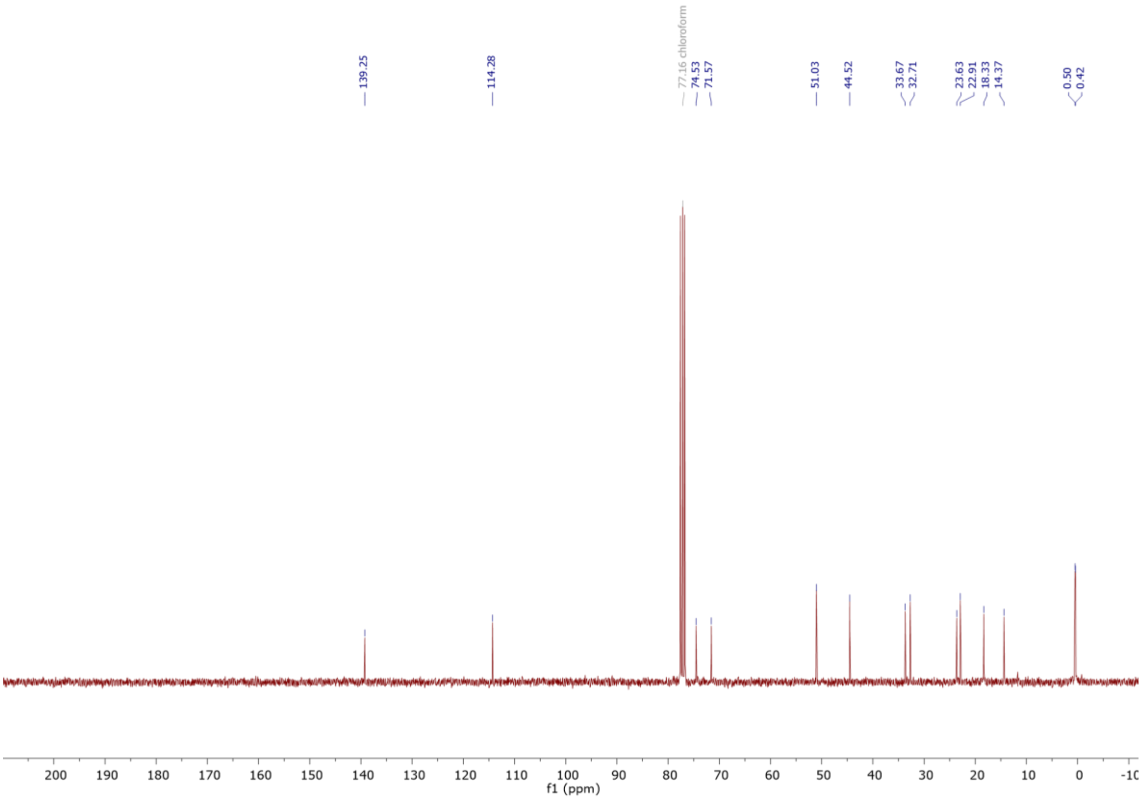

Figure S77. ^{13}C NMR spectrum of 4l

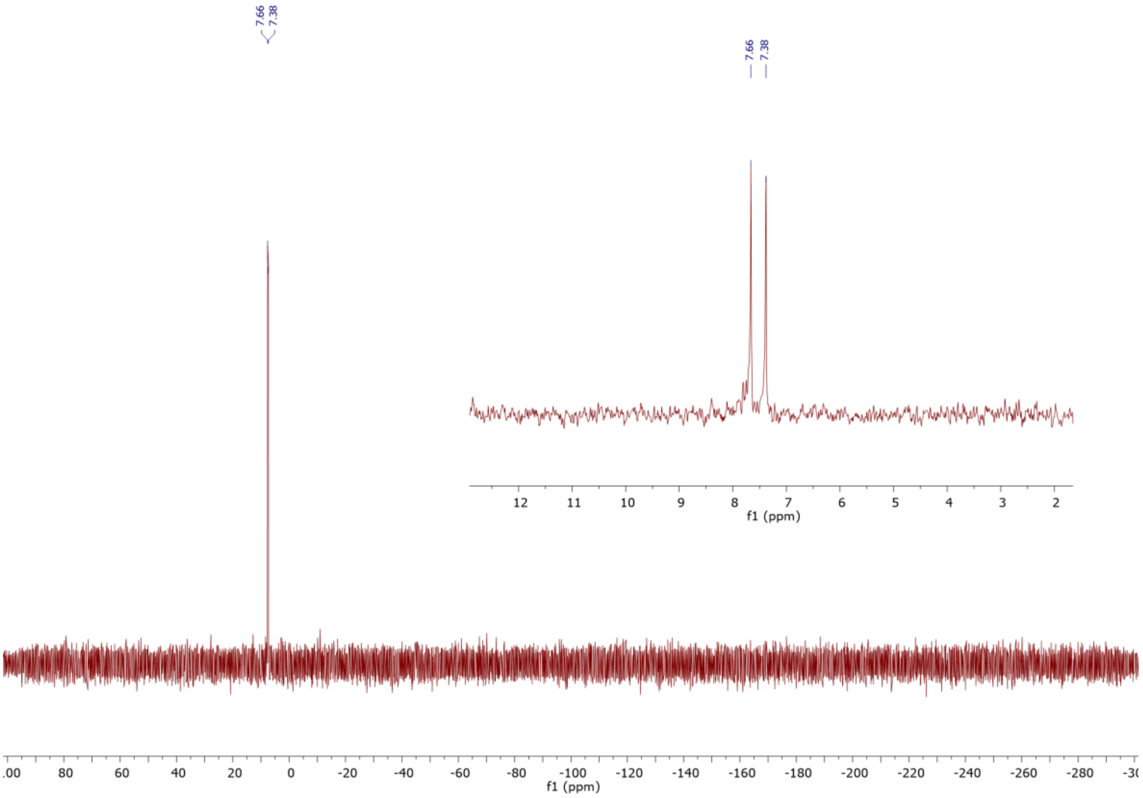

Figure S78. ^{29}Si NMR spectrum of 4l

2.27. Product 4m

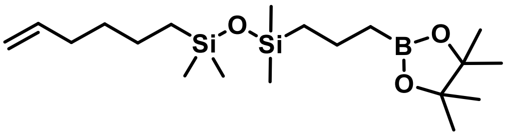

^{1}H NMR (300 MHz, $CDCl_3$) δ 5.81 CH_2=<u>CH</u>R (ddt, J = 16.9, 10.1, 6.7 Hz, 1H), 5.09 – 4.81 <u>CH_2</u>=CHR (m, 2H), 2.04 (q, J = 7.0 Hz, 2H), 1.58 – 1.27 (m, 6H), 1.24 (s, 12H), 0.82 BCH_2 (t, J = 7.6 Hz, 2H), 0.53 $SiCH_2$ (m, 4H), 0.02 $SiMe_2$ (s, 12H).
^{13}C NMR (75 MHz, $CDCl_3$) δ 139.32, 114.25, 82.94, 33.70, 32.74, 24.97, 22.94, 21.83, 18.36, 18.10, 0.56, 0.52.
^{29}Si NMR (79 MHz, $CDCl_3$) δ 7.20, 7.00.
MS (EI, m/z): 269.2 $[M-15]^+$, 203.2 (8.9), 202.2 (18.0), 201.0 (100), 199.8 (23.1), 186.9 (7.5), 175.0 (12.4), 158.9 (30.1), 144.9 (7.2), 134.1 (7.6), 132.9 (56.1), 116.8 (5.5), 83.0 (12.9), 72.9 (8.1),

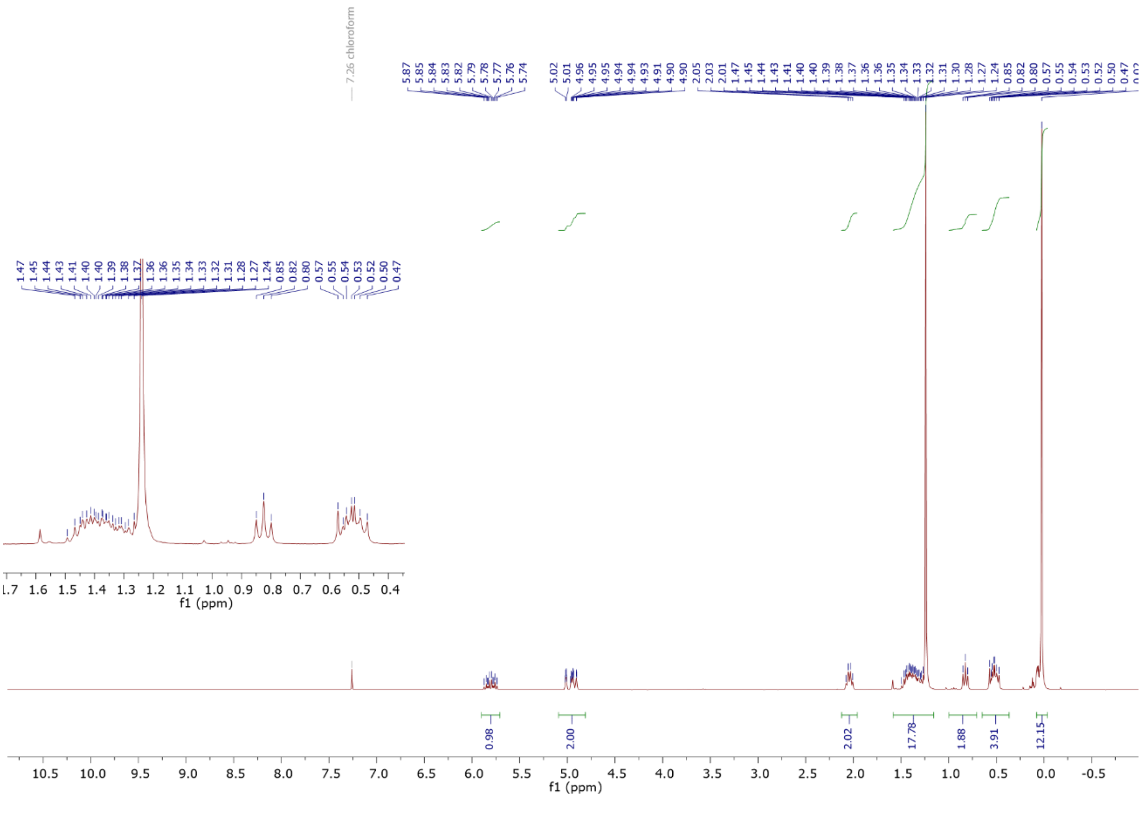

Figure S79. ^{1}H NMR spectrum of 4m

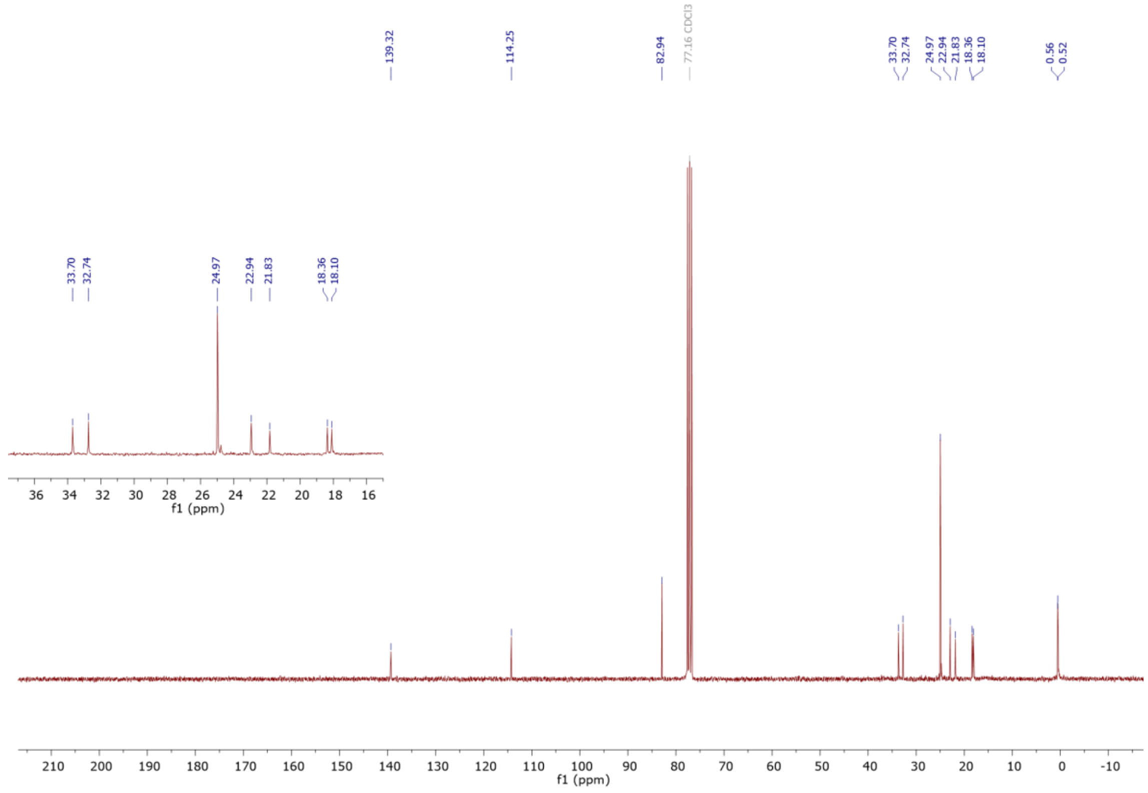

Figure S80. ^{13}C NMR spectrum of 4m

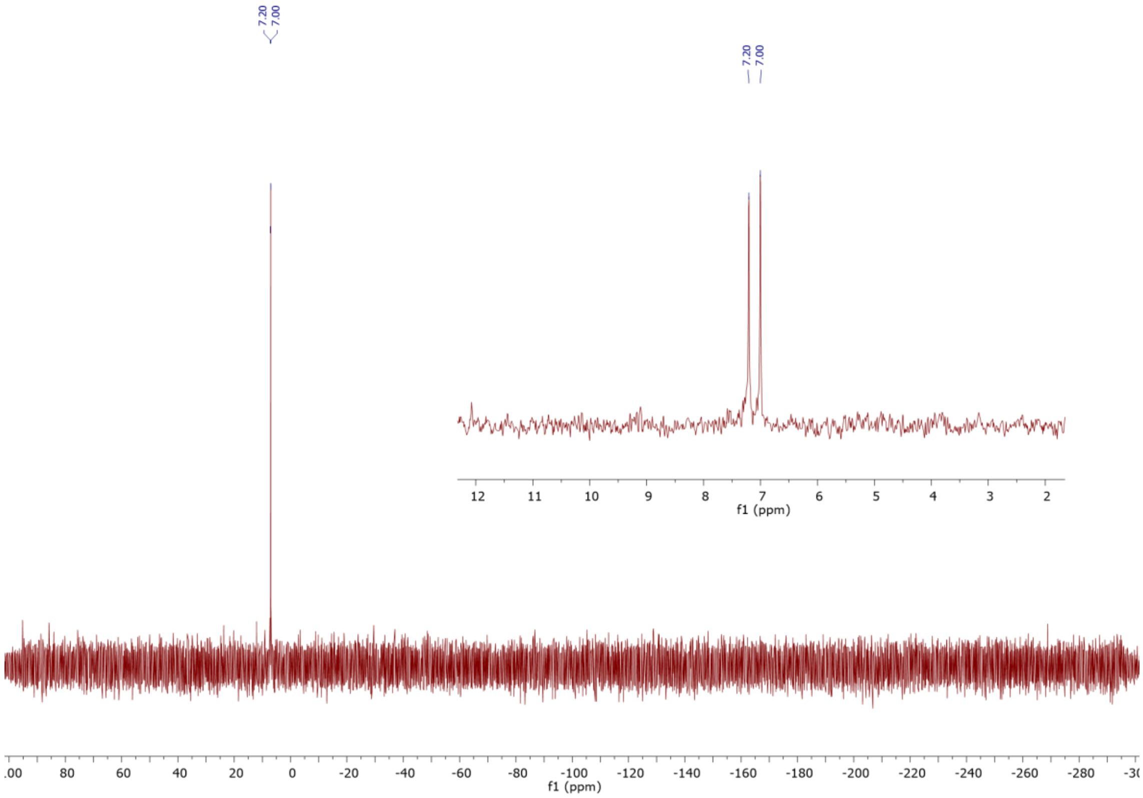

Figure S81. ^{29}Si NMR spectrum of 4m
